# Neurodevelopmental miR-135a-1-3p and miR-135b-3p promote long-distance axon regeneration after injury in the central visual pathway

**DOI:** 10.1016/j.omta.2026.201826

**Published:** 2026-07-30

**Authors:** Jacob H. Brady, Agnieszka Lukomska, Arnav J. Balaji, Clyde D’Souza, Ephraim F. Trakhtenberg

**Affiliations:** 1 Department of Neuroscience, University of Connecticut School of Medicine, 263 Farmington Ave, Farmington, CT 06030, USA

**Keywords:** axon regeneration, neuroprotection, optic nerve injury, optic neuropathy, retinal ganglion cell, neuron gene therapy, CNS repair, micro-RNAs, miR-135

## Abstract

Micro-RNAs (miRNAs) play roles in the mammalian central nervous system (CNS) development and disease but the functions of specific miRNA families’ members in defined neuronal types are poorly understood. Here, we characterized the miR-135 family members in the mouse CNS retinal ganglion cell (RGC) and cortical projection neurons development, and tested whether reverting expression toward embryonic levels for the identified developmentally regulated miR-135 family members may reactivate the intrinsic axon growth capacity following axonal injury in the adult CNS. We found that two-out-of-five miR-135 family members, miR-135a-1-3p and miR-135b-3p, were developmentally downregulated, with further downregulation after axonal injury, in neurons. Importantly, RGC-targeted mimics of miR-135a-1-3p and miR-135b-3p promoted neuroprotection and axon regeneration following optic nerve injury, with axons regrowing through a segment of the central visual pathway when co-treated with an axon growth-supporting small fibronectin-based recombinant protein. Finally, transcriptomic profiling of the treated RGCs showed that genes involved in neurodevelopment/neuroprotection were embedded in the gene networks affected by the treatments. Thus, miR-135a-1-3p and miR-135b-3p are developmentally downregulated in the mammalian CNS projection neurons and present options for translation toward CNS injury treatments.

## Graphical abstract

**Figure:**
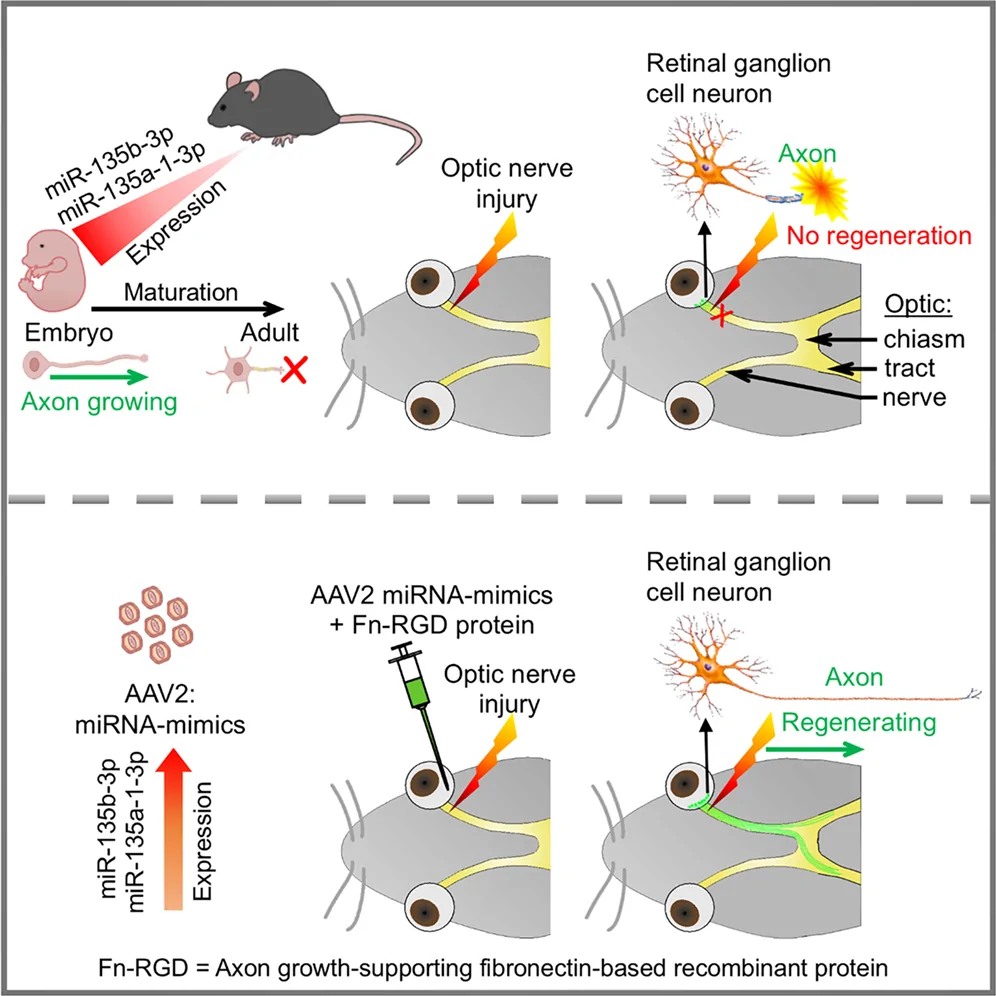

## Teaser

Functions of neuronal miRNAs are not fully understood and no clinical treatments exist for regenerating damaged CNS axons. To address these barriers, Trakhtenberg and colleagues identified a neurodevelopmental miRNA mechanism for translation toward CNS injury treatment, by showing that mimics of developmentally downregulated miR-135a-1-3p and miR-135b-3p regulate gene networks and axon regeneration.

## Introduction

The failure of central nervous system (CNS) projection neurons to regenerate axons over long distances after white matter injury to the brain, spinal cord, or optic nerve/tract results in permanent disabilities.^1^^,^^2^^,^^3^^,^^4^^,^^5^^,^^6^^,^^7^^,^^8^^,^^9^ Like other mammalian CNS projection neurons, retinal ganglion cells (RGCs) do not spontaneously regenerate axons injured in the optic nerve crush (ONC) model of traumatic optic neuropathy (TON).^10^^,^^11^^,^^12^ Some injured neurons at least partially revert their transcriptomes toward an embryonic cell state^13^^,^^14^^,^^15^ in a failed attempt to fully reactivate intrinsic axon growth mechanism (which was inactivated during maturation).^16^ However, several developmentally regulated mRNAs were found to control the developmental decline^17^^,^^18^ in intrinsic capacity of RGCs (and other CNS projection neurons^13^) to grow axons, and targeting these genes promoted axon regeneration after injury.^14^^,^^19^^,^^20^^,^^21^^,^^22^^,^^23^^,^^24^^,^^25^ MicroRNAs (miRNAs) were also found to regulate axon growth and regeneration.^26^^,^^27^^,^^28^^,^^29^^,^^30^^,^^31^^,^^32^^,^^33^^,^^34^^,^^35^^,^^36^^,^^37^^,^^38^^,^^39^ These advances highlight the need for studying the specific miRNA family members’ roles in axon growth of defined neuronal types, which we pursued in these studies. MiRNAs are annotated based on their sequences^40^ and could concurrently target multiple mRNAs in the same cell to regulate cellular processes.^40^^,^^41^ miRNA families include miRNAs’ 5p and 3p arms (i.e., two miRNAs derived from a single pre-miRNA precursor, where one arm could become dominant or both arms could regulate different mRNAs), as well as miRNAs expressed from different genomic loci with homologous seed regions but varying flanking sequences; each member may target different mRNAs, unless the sequences are identical, in which case they are termed “multicopy.”^42^^,^^43^^,^^44^ Our exploratory small-RNA-seq studies identified an association of the miR-135 family with RGC development (see data below). Because a multicopy member of this family (see below) targets the Klf4 transcription factor,^22^ a developmental suppressor of axon growth, to promote axon regeneration,^39^ we prioritized the previously unstudied and biologically distinct miR-135 family members (that do not target Klf4) for investigation in RGC and cortical neuron development and axon regeneration after ONC injury.

**Figure 1:**
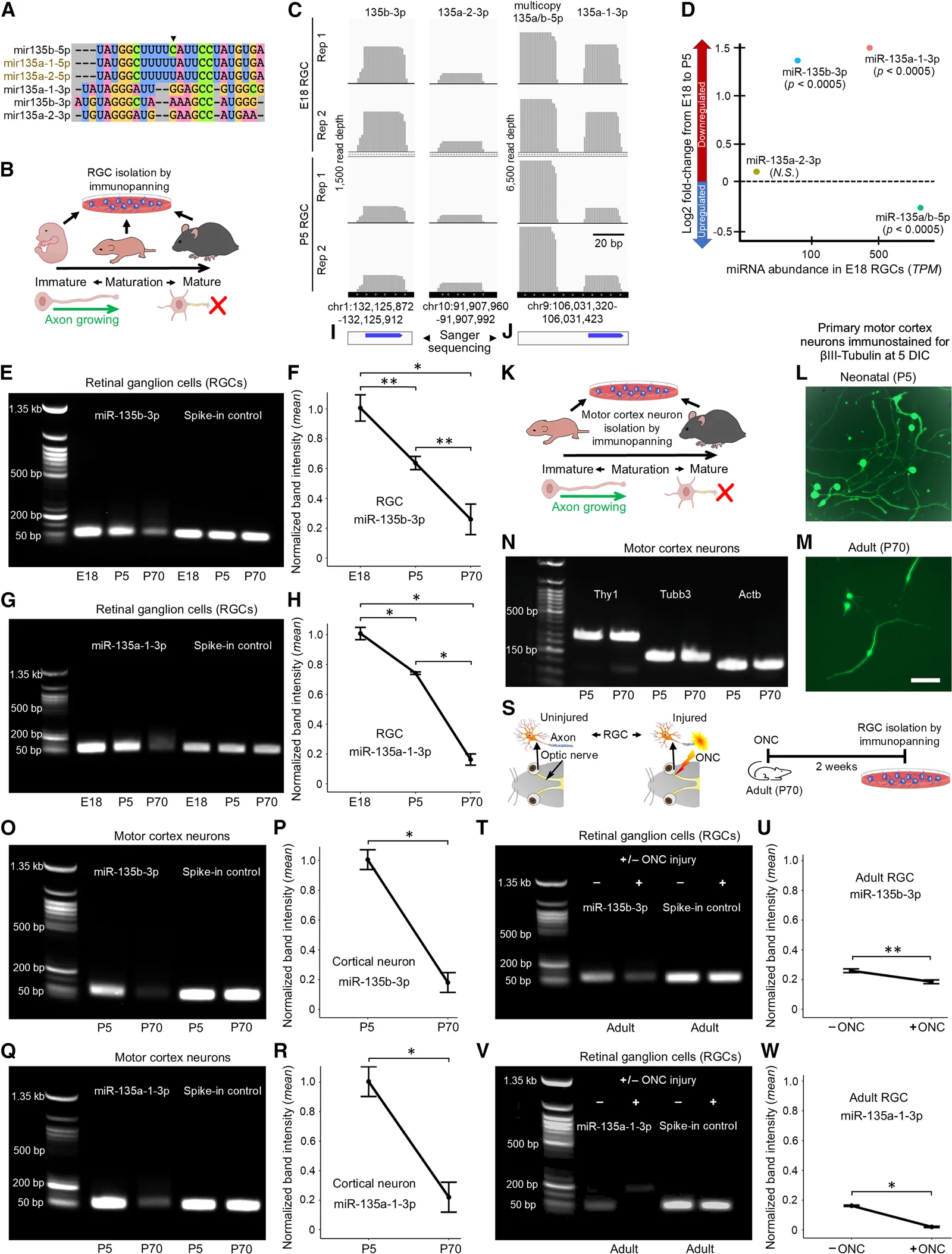
Characterization of miR-135 miRNA family members expression in CNS neurons during developmental maturation and after ONC injury (A) Multiple sequencing alignment (MSA, Clustal Omega) of miR-135 miRNA family members shows substantial differences in the sequences of 3p arms and “multicopy” 5p arms. The “multicopy” miR-135a-1-5p and miR-135a-2-5p are annotated in brown font; miR-135b-5p is near-identical to the “multicopy,” differing in only 1 bp (indicated by arrowhead). (B) Schematic of experimental design for small-RNA-seq and sqPCR analysis of the maturing RGCs isolated by Thy1-immunopanning after depletion of macrophages (see “methods”). (C) Visualization of normalized reads expression from RGC small-RNA-seq aligned to the miR135 loci in the mouse genome. From left to right: miR-135b-3p mapping to chr1; miR-135a-2-3p mapping to chr10; and miR135a/b-5p and miR-135a-1-3p mapping to chr9. miR135a-5p “multicopy” of miR-135a-1-5p and miR-135a-2-5p, visualized here with net normalized reads aligned to the miR-135a-1-5p chr9 locus (as it cannot be determined from which of the two loci it originated) along with miR-135b-5p reads which differ by only 1 bp (see “methods”). Peaks aligned to miR-135b-3p and miR-135-a-1-3p encoding DNA regions are substantially higher in the E18 relative to P5 RGCs. Alignments from two replicates for each RGC age are shown; visualizations are using IGV Viewer. (D) DESeq2 differential expression abundance plot, with abundances of miR-135 family members in E18 RGCs (on *x* axis) versus FC from E18 to P5 RGCs on (*y* axis), visualizing miRNAs’ abundance and developmental-regulation during RGC maturation. Statistical significance (indicated by ∗*p* < 0.001) of developmental regulation (E18 relative to P5 RGCs) determined using differential expression analysis by DESeq2, as marked; *N.S.*, not significant. Mean (SEM) shown; *n* = 2. Only miR-135b-3p and miR-135a-1-3p were downregulated significantly, while other miR-135 family members were either not developmentally regulated or modestly upregulated. TPM, transcripts per million, which is a common normalization method for expression in small-RNA-seq analysis (see “methods”). (E–H) Representative images of sqPCR analysis of miR-135b-3p and miR-135a-1-3p expression at E18, P5, and P70 (adult) stages of RGC development confirm that the sequences detected by small-RNA-seq are miR-135b-3p (E) and miR-135a-1-3p (G), which along with the adaptors match the expected ∼85 bp gel bands. Quantitation of miR-135b-3p and miR-135a-1-3p gel bands intensity in samples prepared from E18, P5, and P70 RGCs (as marked) shows decline in their levels during maturation. Mean (SEM) shown; *n* = 3 samples for each condition (each sample was prepped from different animals). Data analyzed using ANOVA, overall *F* = 20.7, *p* < 0.001 for miR-135b-3p (F) and overall *F* = 174.6, *p* < 0.001 for miR-135a-1-3p (H), with *p* values of pairwise comparisons determined by post hoc LSD and significant differences indicated by ∗*p* < 0.001 and ∗∗*p* < 0.05. (I and J) Sanger sequencing of sqPCR products purified from the gel bands corresponding to miR-135b-3p (I) and miR-135a-1-3p (J) aligned to their respective loci (visualized in C above). Each Sanger sequencing result aligned to only one site in the genome, and blasting against the mouse miRbase database confirmed specificity of the sequences (miR-135b-3p score 105, *e* value 0.002; miR135a-1-3p score 105, *e* value 0.004). (K–N) Schematic of experimental design for sqPCR analysis of the maturing motor cortex neurons isolated by Thy1-immunopanning after depletion of macrophages (see “methods”) (K). Primary neurons Thy1-immunopanned from P5 (L) and P70 adult (M) motor cortices cultured in defined medium (see “methods”) and immunostained for neuronal-marker βIII-Tubulin to visualize neurite growth at 5 days in culture (DIC); scale bars, 100 μm. PCR for neuronal marker genes Tubb3 and Thy1 (which is enriched in projection neurons), along with the housekeeping Actb gene, confirmed enrichment of projection neurons isolated from single-cell suspension of P5 and P70 motor cortices by immunopanning (N). (O–R) Representative images of sqPCR analysis of miR-135b-3p and miR-135a-1-3p expression at P5 and P70 (adult) stages of motor cortex neuron development confirm that miR-135b-3p (O) and miR-135a-1-3p (Q) miRNAs match the expected ∼85 bp (along with the adaptors) gel bands. Quantitation of miR-135b-3p and miR-135a-1-3p gel bands intensity in samples prepared from P5 and P70 motor cortex neurons (as marked) shows decline in their levels during maturation. Mean (SEM) shown; *n* = 3 samples for each condition (each sample was prepped from different animals). Data analyzed using *t* test two-tailed, with significant differences indicated by ∗*p* < 0.001 for miR-135b-3p (P) and miR-135a-1-3p (R). (S) Schematic of experimental design for sqPCR analysis of adult uninjured and ONC-injured RGCs. ONC injury performed in P70 (adult) mice, and animals sacrificed for isolation of RGCs by immunopanning (as described in B) at 2 weeks post-ONC. For control, RGCs were isolated the same way from age-matched uninjured mice (see “methods”). (T–W) Representative images of sqPCR analysis of miR-135b-3p and miR-135a-1-3p expression in uninjured and ONC-injured adult RGCs confirm that miR-135b-3p (T) and miR-135a-1-3p (V) miRNAs match the expected ∼85 bp (along with the adaptors) gel bands. Quantitation of miR-135b-3p and miR-135a-1-3p gel bands intensity in samples prepared from uninjured and injured RGCs (as marked) shows further post-injury decline in their levels. Mean (SEM) shown; *n* = 3 samples for each condition (each sample was prepped from different animals). Data analyzed using *t* test two-tailed, with significant differences indicated by ∗*p* < 0.001 for miR-135b-3p (U) and ∗∗*p* < 0.05 for miR-135a-1-3p (W).

The miR-135 family is comprised of five miRNA sequences which originate from Chr1, Chr9, and Chr10, as follows: the miR-135a′s 5p arms from Chr9 and Chr10 are the miR-135a-5p multicopy which is near-identical to miR-135b-5p from Chr1 that differs by only one base pair (bp), whereas the 3p arms differ from the 5p arms by almost 50%, with miR-135a-1-3p, miR-135a-2-3p, and miR-135b-3p originating from Chr9, Chr10, and Chr1, respectively (Figure 1A). Thus, because the miR-135 family 5p and 3p arms differ from each other, they target different mRNAs. The miR135a-5p multicopy, along with the nearly identical miR-135b-5p, modestly promote short-distance RGC axon regeneration up to approximately ∼1 mm past the ONC site, potentially through targeting Klf4 in mature RGCs.^39^ However, Klf4 is only transiently upregulated postnatally to irreversibly suppress RGCs’ embryonic intrinsic axon growth capacity and is then downregulated in fully mature RGCs.^22^ Thus, it is unclear how targeting Klf4 in already mature RGCs could be effective.^39^ It is also unknown whether other miR-135 family members, which are distinct biologics that target different genes, are involved in neuronal development and/or axon regeneration. Here, we addressed these questions by: analyzing expression of miR-135 family members in RGC and cortical neuron development, testing whether the previously unstudied miR-135 family members regulate axon regeneration, probing rationale for targeting Klf4 in already mature RGCs, and exploring gene networks affected by axon-regenerating miR-135 family members identified herein.

## Results

### Characterization of miR-135 family members’ expression patterns in RGC and cortical neurons

Alignment of miR-135 family members shows that, while miRNAs share common features (highlighted in orange and green), the near-identical (i.e., only 1 bp mismatch) 5p arm sequences differ by almost 50% from the 3p arm sequences (Figure 1A). Thus, these 5p and 3p arms’ miRNAs are distinct biologics that target different mRNAs (as detailed below). We used small-RNA-seq to profile miR-135 family members in RGCs purified (by Thy1-immunopanning after depletion of macrophages) from embryonic (E18) and postnatal (P5) developmental stages (see “methods”; Figure 1B). We selected E18 and P5 ages, because RGC’s developmental axon growth capacity is still at its peak in E18 and then declines shortly after birth and is substantially reduced by P5.^17^^,^^18^^,^^45^ Small-RNA-seq showed that, 5p arms’ miR135a/b-5p (multicopy +1 bp mismatching isomiRs) are modestly upregulated, while 3p arm miR-135a-2-3p is not significantly regulated, during RGC maturation (Figures 1C and 1D). Thus, despite the treatment with miR135a/b-5p mimics modestly promoting RGC axon regeneration after ONC,^39^ their endogenous expression patterns are not associated with positive regulation of developmental axon growth, which declines postnatally. However, the 3p arms, miR-135a-1-3p and miR-135b-3p, were downregulated substantially during RGC maturation (Figures 1C and 1D). We validated developmental regulation by a semi-quantitative PCR (sqPCR) method,^37^ which confirmed miR-135a-1-3p and miR-135b-3p downregulation through postnatal stage of maturation and showed further downregulation by adult age (Figures 1E–1H). The specificity of the miRNAs’ amplification in sqPCR was confirmed by genome-aligned Sanger sequencing, which matched the reads from small-RNA-seq (see “methods”; Figures 1I and 1J).

Then, we analyzed miR-135a-1-3p and miR-135b-3p expression in maturation of cortical neurons. Corticospinal motor neurons grow axons along the spinal cord through the first 2 weeks of postnatal development,^46^^,^^47^ which differs from the RGC’s developmental axon growth capacity, which peaks through E18 and declines shortly after birth.^18^ Thus, cortical neurons were isolated by immunopanning for Thy1, which is expressed on cortical and RGC projection neurons,^48^ from the primary motor cortex single-cell suspension of P5 and P70 mice (macrophages were depleted first, see “methods”; Figure 1K). We have previously validated the different neurite growth capacities of the isolated immature and adult RGCs in culture^21^^,^^49^ (that were consistent with prior reports^18^), and confirmed here neurite growth capacity of the isolated P5 cortical neurons relative to the adult cortical neurons which barely grow neurites (Figures 1L and1M). Isolation of P5 and P70 cortical neurons was validated using PCR for neuronal marker-genes Tubb3 (encodes βIII-Tubulin) and Thy1, and control housekeeping gene Actb (encodes β-actin), visualized by gel electrophoresis in samples from both ages (Figure 1N). Analysis by sqPCR showed that miR-135a-1-3p and miR-135b-3p were developmentally downregulated in the motor cortex neurons (Figures 1O–1R). We also tested whether miR-135a-1-3p and miR-135b-3p respond to axonal ONC injury (Figure 1S) and found that both are further reduced in the RGCs by 2 weeks post-ONC (Figures 1T–1W). These data suggest that developmental-downregulation and, potentially, roles of miR-135a-1-3p and miR-135b-3p may be conserved across CNS projection neurons.

### miR-135a-1-3p and miR-135b-3p mimics promote neuroprotection and axon regeneration after injury *in vivo*

**Figure 2:**
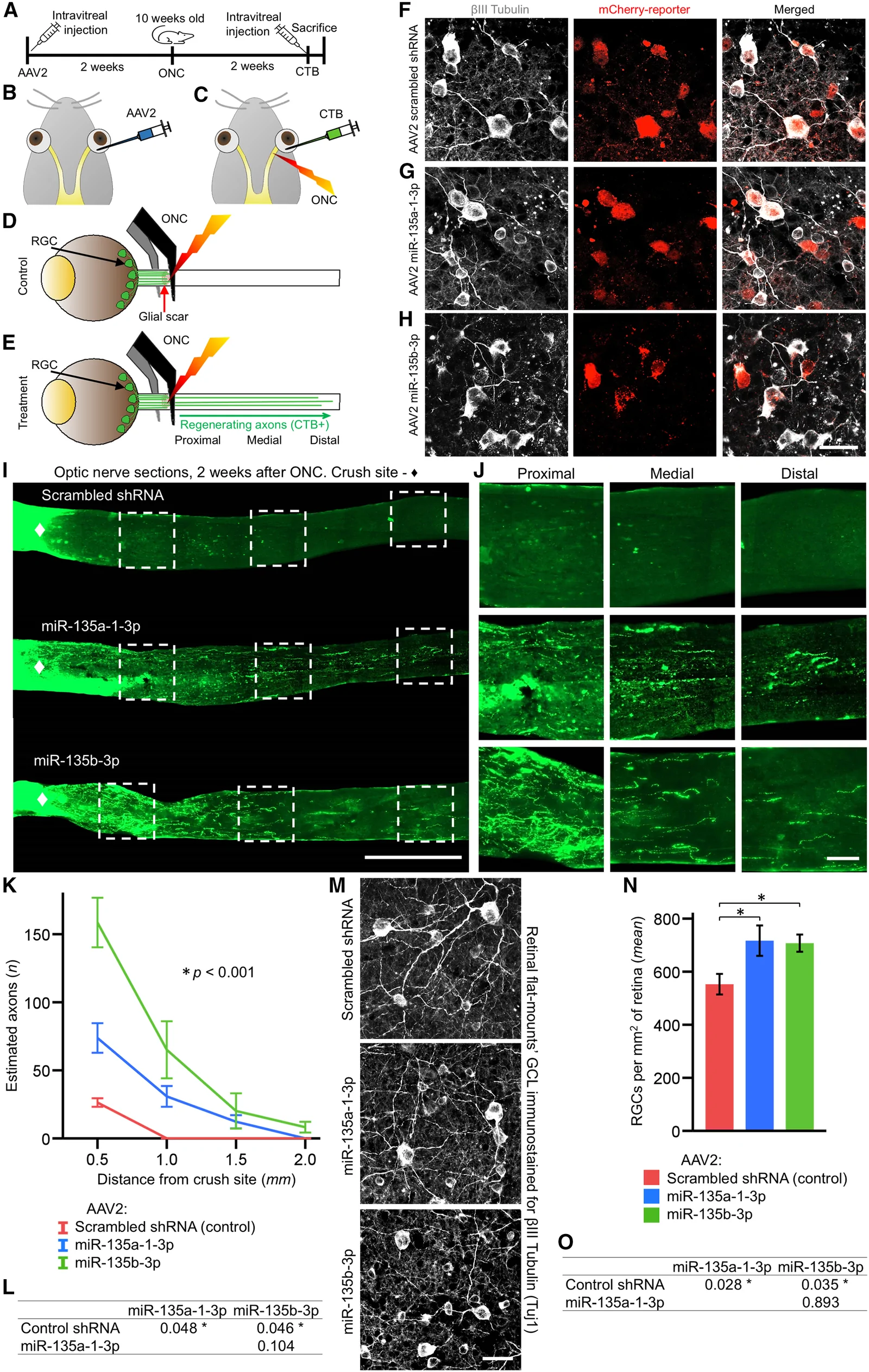
AAV2 miR-135a-1-3p and miR-135b-3p mimics promote RGC survival and axon regeneration after optic nerve injury (A–E) Experimental timeline (A) with schematic representations: AAV2 viral vectors injected intravitreally in 8-week-old mice (B), and 2 weeks later ONC performed (C). Animals sacrificed for histological analysis 2 weeks post-ONC. For axon regeneration studies (shown later in I–K), an axonal tracer CTB (conjugated to Alexa Fluor 488 dye) is injected intravitreally 1 day before sacrifice (C). Because axons are severed by the injury, CTB is not transported beyond the ONC site (D), unless axons regenerated in response to a treatment (E). (F–H) Confocal microscopy with 63× objective was used for determining co-localization of vector reporters and RGC marker in the same cells. High-magnification representative images of the immunostained retinal flatmounts show that: an mCherry reporter of transduction/expression localized to the βIII-Tubulin+ cells (βIII-Tubulin is a neuronal marker which labels the RGCs within the retinal GCL), and that transduced cells are detected at comparable proportions across conditions: scrambled shRNA-control (F), miR-135a-1-3p-mimic (G), and miR-135b-3p-mimic (H), as marked. Scale bars, 20 μm. (I and J) Representative images of the optic nerve longitudinal sections with CTB-labeled axons at 2 weeks post-ONC from the animals pre-treated with AAV2 expressing miR-135a-1-3p-mimic, miR-135b-3p-mimic, or scrambled-control shRNA, as marked (I). Insets: representative images of the optic nerve regions proximal and distal to the injury site are magnified for better visualization of the axons or their absence (J). The edges of the tissue were optically trimmed (i.e., cropped-out) due to artifactual autofluorescence that is common at tissue edges (see “methods”). Scale bars, 500 μm (main images), 50 μm (insets). (K and L) Quantitation of CTB-labeled regenerating axons at 2 weeks post-ONC, at increasing distances from the injury site, after pre-treatment with AAV2 expressing miR-135a-1-3p-mimic, miR-135b-3p-mimic, or scrambled-control shRNA, as marked (K). Data analyzed using repeated measures ANOVA with post hoc LSD, sphericity assumed, overall *F* = 26.4, *p* < 0.001; with *p* values of pairwise comparisons determined by post hoc LSD shown in the table, and significant differences (*p* < 0.05) indicated by an asterisk ∗ (L). Mean (SEM) shown; *n* = 4 cases per group. (M–O) Representative images of the retinal flatmounts immunostained for βIII-Tubulin at 2 weeks post-ONC, pre-treated with experimental and control AAV2 vectors, as marked. Scale bars, 20 μm (M). Quantitation of RGC (βIII-Tubulin+ cells) survival in retinal flatmounts’ GCL at 2 weeks post-ONC, from the treatment conditions as marked (N). Data analyzed using ANOVA, overall *F* = 4.94, *p* < 0.05, with *p* values of pairwise comparisons determined by post hoc LSD shown in the table, and significant differences (*p* < 0.05) indicated by an asterisk ∗ (O). Mean (SEM) shown, *n* = 4 cases per group.

To test whether reinstating miR-135a-1-3p and miR-135b-3p expression in injured adult RGCs toward embryonic (i.e., higher) levels affects neuroprotection and/or axon regeneration, we used AAV2 vectors expressing miR-135a-1-3p-mimic, miR-135b-3p-mimic, or scrambled-control shRNA, and co-expressing an mCherry reporter, for intravitreal targeting in RGCs. To confirm persistence of AAV2 expression in RGCs after injury, the viruses were injected intravitreally in adult mice, and 2 weeks later ONC was performed (experimental design shown in Figures 2A–2E). At 2 weeks post-ONC, the retinas were immunostained for βIII-Tubulin neuronal marker, which labels the RGCs within the retinal ganglion cell layer (GCL). Co-localization of an mCherry-reporter and βIII-Tubulin signals by confocal microscopy confirmed vector expression in RGCs, with transduction efficiency of ∼30% within a range of prior reports that also used intravitreal AAV2 for targeting RGCs^12^^,^^21^^,^^25^^,^^50^^,^^51^^,^^52^ (Figures 2F–2H). Then, we tested whether miR-135a-1-3p and/or miR-135b-3p mimics would promote RGC survival and axon regeneration after injury. We used an established assay in which the optic nerve is injured by crush to sever all RGC axons, and then RGC survival and axon regeneration are quantified at 2 weeks post-injury, using βIII-Tubulin immunostaining and anterograde axon labeling with cholera toxin subunit B (CTB) injected 1 day before sacrifice, respectively (Figure 2A).^12^^,^^25^^,^^50^^,^^51^^,^^52^^,^^53^ We found that by 2 weeks post-ONC, relative to only minor axonal sprouting past the injury site detected in control animals, miR-135a-1-3p and miR-135b-3p mimics promoted axon regeneration reaching approximately ∼2 mm past the injury site (Figures 2I–2L). Both treatments also promoted RGC survival compared to the control group (Figures 2M–2O).

**Figure 3:**
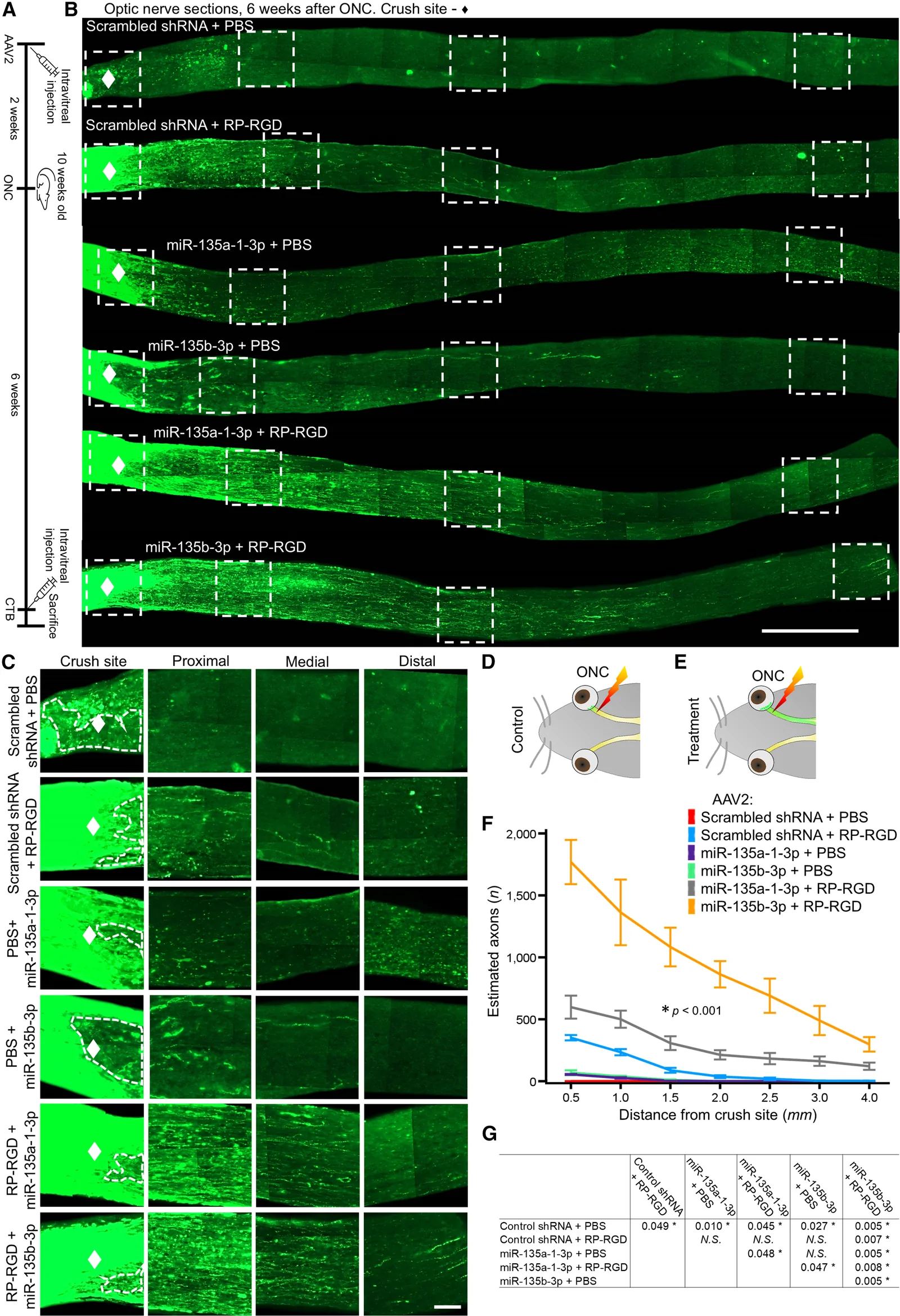
Co-treating with RP-RGD and AAV2 miR-135a-1-3p or miR-135b-3p mimics augments axon regeneration sustained over longer period (A) Experimental timeline: 8-week-old mice pre-treated with AAV2 expressing miR-135a-1-3p-mimic, miR-135b-3p-mimic, or scrambled-control shRNA, and co-treated with RP-RGD or vehicle. ONC injury performed 2 weeks later. Animals sacrificed for histological analysis 6 weeks post-ONC. Axonal tracer CTB injected intravitreally 1 day before sacrifice. (B–E) Representative images of the optic nerve longitudinal cryosections with CTB-labeled axons at 6 weeks post-ONC from the animals treated as marked (B). Insets: representative images of the optic nerve regions (outlined by dashed boxes in B) proximal and distal to the injury site magnified for better visualization of the axons or their absence, and of the ONC site with a larger retrogradely degenerating pre-crush area (indicated by the diminished CTB signal outlined by dashed line) in the control compared to treatment conditions (C). Schematic of the injured central visual pathway segment containing the optic nerve in the control condition (D), and a representation of axon regeneration through the full-length of the optic nerve achieved by the co-treatments, compared to control, at 6 weeks post-ONC (E). The edges of the tissue were optically trimmed (i.e., cropped-out) due to artifactual autofluorescence that is common at tissue edges (see “methods”). Scale bars, 500 μm (main images), 50 μm (insets). (F and G) Quantitation of CTB-labeled regenerating axons at 6 weeks after ONC, at increasing distances from the injury site along the optic nerve, from the animals treated as marked (F). Data analyzed using repeated measures ANOVA, sphericity assumed, overall *F* = 108.5, *p* < 0.001, with *p* values of pairwise comparisons determined by posthoc LSD shown in the table, and significant differences (*p* < 0.05) indicated by an asterisk ∗ (G). Mean (SEM) shown; *n* = 4 cases per group.

Next, we asked whether the positive effects of these miRNA gene therapies are sustained over a longer term, and whether their effectiveness could be enhanced by co-treating with a small recombinant protein containing fibronectin’s RGD-motif (RP-RGD), which we previously developed as an axon growth-supporting factor that also augments the efficacy of other treatments.^49^ AAV2 vectors were administered as above at 2 weeks pre-injury, and RP-RGD or vehicle were injected intravitreally immediately after ONC. RGC survival and axon regeneration were analyzed at 6 weeks post-ONC, which is a later time point we used for evaluating other axon regeneration treatments in prior studies^49^^,^^51^ (Figure 3A). We found no spontaneous axon repair or regeneration in the control condition. Moreover, diminished CTB signal in most of the pre-ONC area indicates retrograde axon degeneration from the injury site toward the retina, which became apparent by 6 weeks (compared to 2 weeks in Figures 2I and 2J) in the control condition^6^^,^^54^ (Figures 3B and 3C). As compared to control, significant axon regeneration was sustained (relative to regeneration at 2 weeks) in all the treatment conditions, without substantial retrograde degeneration in the pre-ONC area which occurred in control, at 6 weeks post-ONC (Figures 3B–3E). Remarkably, compared to individual treatments, in the experimental co-treatment conditions substantially more long axons regenerated (Figures 3F and 3G), with some axons regenerating through the entire optic nerve and optic chiasm and in half of the cases entering the optic tracts (Figures S1A**–**S1D). Furthermore, RGC survival was significantly higher in the individual treatments compared to the control condition, and to an even greater extent in the co-treatments, at 6 weeks post-ONC (Figures S1A**–**S1C). As most untreated RGCs die progressively after ONC injury,^6^^,^^54^ sustainment of RGC survival over a long term is important. Thus, co-treatment with RP-RGD and miR-135a-1-3p or miR-135b-3p promotes survival and long-distance axon regeneration of RGCs after ONC injury.

### miR-135a/b-5p and miR-135a/b-1-3p predicted to target different genes

To explore whether the 3p arms’ miR-135a-1-3p or miR-135b-3p targets overlap those of 5p arms’ miR-135a/b-5p, we used the bioinformatic tool miRanda, which predicts miRNAs’ gene targets^55^^,^^56^ (Figure 1A). For genes expressed in injured adult RGCs based on scRNA-seq, we identified mRNA transcript isoforms by bulk-mRNA-seq profiling of injured RGCs (see “methods”). We then analyzed for potential miRNA substrates within the 3′ UTRs of these transcripts, because 3′ UTRs are established to be preferentially targeted by miRNAs in general^41^ (the 3′ UTR sequences are in Table S1; see “methods”). The 5p and 3p arms’ miRNAs were predicted to target 3′ UTRs of different subsets of mRNA transcripts expressed in injured adult RGCs (shown rank-ordered by miRanda in Figures S2A**–**S2G). Because Klf4 peaks only transiently in postnatal RGCs to switch-off embryonic intrinsic axon growth capacity and then downregulates by adult age,^22^ targeting Klf4 in adult RGCs may promote axon growth^39^ only if Klf4 is re-upregulated after injury. Although scRNA-seq^57^ detected only modest Klf4 levels in the ONC-injured RGC subtypes (annotated as described previously^14^^,^^57^; Figure S2H), this is consistent with the prior report,^39^ as Klf4 was among the top predicted 5p arm miRNAs’ targets (Figures S2A, S2B, and S2E). However, Klf4 is not a predicted target of 3p arms’ miR-135b-3p or miR-135a-1-3p (Figures S2C**–**S2D, S2F and S2G). Thus, 5p and 3p arms miR-135 miRNAs are predicted to target different genes in the RGCs.

### miR-135a-1-3p and miR-135b-3p mimics affect gene networks in injured RGCs

miRNAs could co-target multiple mRNAs to regulate cellular processes through indirectly affected downstream gene networks.^40^^,^^41^ Thus, to build hypotheses regarding the underlying mechanisms, we utilized the gene annotation/ontology (GO) database for exploring the gene networks affected by the treatments.^58^^,^^59^ We used a pipeline we previously described^37^ to explore the differentially expressed genes (DEGs) by bulk-mRNA-seq of miR-135a-1-3p- and miR-135b-3p-mimic-treated RGCs, relative to scrambled-control shRNA-treated RGCs (experimental timeline in Figure S2I; the DEG list is in Table S2; see “methods”). Although the enriched GO terms (see “methods”) were generic, several genes previously associated with neurodevelopment and/or neuroprotection were in the gene networks: Dpysl2,^24^^,^^60^^,^^61^ Sox11,^62^^,^^63^^,^^64^ and Gal^65^^,^^66^ were upregulated in both treatments, whereas Kif5b^67^ was upregulated in the miR-135a-1-3p treatment while Anxa2^68^ and Myc^69^ were upregulated and Pten^53^ downregulated in miR-135b-3p treatment (Figures S2J and S2K). Thus, the affected gene networks embed downstream regulators of axon growth.

## Discussion

The roles of miRNAs in neuronal development and repair after CNS injury continue to emerge.^26^^,^^27^^,^^28^^,^^29^^,^^30^^,^^31^^,^^32^^,^^33^^,^^34^^,^^35^^,^^36^^,^^37^^,^^38^^,^^39^ Studying miRNAs in an arm-specific and neuronal type-specific manner *in vivo* is necessary for advancing the neuronal miRNAs research field. Here, we show that the miR-135 family 3p arms, miR-135a-1-3p and miR-135b-3p, are (1) developmentally downregulated in RGC and motor cortex neurons during maturation-associated loss of intrinsic axon growth capacity,^13^^,^^14^^,^^17^^,^^18^ (2) further downregulated in the RGCs after injury, and (3) targeting injured RGCs via AAV2 with their mimics promotes RGC survival and axon regeneration. Remarkably, axon regeneration promoted by miR-135a-1-3p and miR-135b-3p co-treated with a fibronectin-based small recombinant protein we recently developed^49^ enabled a subset of axons to regenerate through the full-length of the optic nerve, with some axons in half of the cases even traversing the optic chiasm and entering the optic tract by the 6-week post-injury endpoint we tested. Future studies would need to explore the full potential of these treatments by leveraging emerging improved AAV2-based vectors with higher transduction efficiency and using longer-term endpoints which, per prior reports, need to be 10–12-week post-ONC in order to allow for the regenerating axons to reach post-synaptic targets in the brain.^70^^,^^71^ Following determination of anatomical reinnervation of post-synaptic targets in the brain, restoration of at least simple visual functions could be tested through electrophysiological and behavioral assays.

miRNAs are a promising class of biologics for therapeutic applications, because of their small size and the ability to concurrently target multiple mRNAs, in the same cell,^40^^,^^41^^,^^42^ that may need to be co-regulated for coordinating complex biological processes such as axonal tissue regeneration. This is consistent with miR-135a-1-3p and miR-135b-3p mimics targeted to injured RGCs affecting GO-linked gene networks that embedded several downstream genes previously associated with neurodevelopment and/or neuroprotection. For example, Dpysl2,^24^^,^^60^^,^^61^ Sox11,^62^^,^^63^^,^^64^ Gal,^65^^,^^66^ Kif5b,^67^ Anxa2,^68^ and Myc^69^ were indirectly upregulated downstream of targets such as Pten,^53^ which were knocked-down by the miRNA mimics. It would be important for future studies to discern between the direct and indirect downstream mRNA targets, by screening the predicted DEGs for direct miRNA-mRNA interaction using reporter assays. However, it may not be possible to recapitulate the full phenotype by targeting a single mRNA, and most alternative approaches also co-target a combination of factors to promote long-distance axon regeneration. Therefore, future directions toward a deeper understanding of the miR-135a-1-3p and miR-135b-3p downstream mechanisms may need to study crosstalk between the cellular pathways regulated by a miRNA in order to determine their relative contributions to neuroprotection and axon regeneration. Future research will also need to determine which RGC subtypes respond better to the treatments, and whether these miRNA mimics are also neurotherapeutic in traumatic spinal cord and brain injury models, particularly if the miR-135 3p and 5p arms’ mimics are co-administered to achieve potentially stronger efficacy.

## Materials and Methods

Materials and methods are provided in the supplemental material along with the supplemental data legends.

## Data and code availability

• The plasmid for miR-135a-1-3p-mimic and miR-135b-3p-mimic AAV2 vectors will be shared for academic purposes. This study did not generate any other new unique reagents.
• RNA sequencing data generated for this study have been deposited in publicly accessible NCBI repository under Gene Expression Omnibus (GEO) accession numbers GSE337069 and under NCBI Sequence Read Archive (SRA) BioProject accession number PRJNA1483696. GEO accession numbers for the previously generated RNA sequencing data used in this study are provided in the supplemental information.
• This paper does not report original code.
• The supplemental data legends are provided in the supplemental information. Any additional information required to reanalyze the data reported in this paper is available from the lead contact upon request.

## Acknowledgments

This work was supported by grants from the 10.13039/100000002National Institutes of Health (10.13039/100000002NIH) NEI-R01-EY029739 and NEI-R01-EY038322, to E.F.T. We are grateful to the High Performance Computing Facility, University of Connecticut for bioinformatics support. We thank Ashiti Damania, William Theune, and Jian Xing (students, University of Connecticut School of Medicine) for technical assistance.

## Author contributions

J.H.B., A.L., A.J.B., and C.D. performed the experiments; E.F.T. designed the study and wrote the manuscript.

## Declaration of interests

The authors declare no competing interests.

## Supplemental information

Document S1. Figures S1 and S2 and supplemental references

Table S1. 3′ UTR sequences in FASTA format for transcripts isoforms determined, based on RNA-seq analysis, as expressed in ONC-injured RGCs Refer to the main text and methods for technical details

Table S2. List of differentially expressed genes (DEGs) with expression and fold-change (FC) values Log2 FC (log2FC) between the average expression in miR-135a-1-3p or miR-135b-3p mimic-treated injured RGCs and either scrambled-control shRNA-treated injured or uninjured RGCs is shown. Significantly (CuffDiff *q* value < 0.05) differentially expressed genes (DEGs) between scrambled-control shRNA-treated injured RGCs, that are also at least 2 log2FC relative to uninjured RGCs and with ≥5 FPKM in the higher-expressing condition, are shown with the columns annotated as indicated. Refer to the main text and methods for technical details

Document S2. Article plus supplemental information
