# Supplementary material for: Neurodevelopmental miR-135a-1-3p and miR-135b-3p promote long-distance axon regeneration after injury in the central visual pathway
Source: Mol Ther Adv. 2026 Jul 30;34(3):201826. doi: 10.1016/j.omta.2026.201826 (PMC13507755; doi:10.1016/j.omta.2026.201826)
Supplement: Supplementary file 1 [file mmc1.pdf]

OMTA, Volume *34*

Supplemental information

Neurodevelopmental miR-135a-1-3p and miR-135b-3p promote long-distance axon regeneration after injury in the central visual pathway

Jacob H. Brady, Agnieszka Lukomska, Arnav J. Balaji, Clyde D'Souza, and Ephraim F. Trakhtenberg

MATERIALS AND METHODS

Animal use, surgeries, intraocular injections. All animal studies were performed at the University of Connecticut Health Center with approval of the Institutional Animal Care and Use Committee and of the Institutional Biosafety Committee, and performed in accordance with the ARVO Statement for the Use of Animals in Ophthalmic and Visual Research. Mice were housed in the animal facility with a 12-h light/12-h dark cycle (lights on from 7:00 AM to 7:00 PM) and a maximum of five adult mice per cage. Food and water were available *ad libitum*. The study used wild-type 129S1/SvImJ mice (The Jackson Laboratory). Optic nerve surgeries and intravitreal injections were carried out on mice of both sexes, 8-12 weeks of age (average body weight 20-26 g) under general anesthesia, as described previously[1-5]. The viruses included AAV2 vectors expressing miR-135a-1-3p-mimic (sequence: 5′-TATAGGGATTGGAGCCGTGGCG-3′), miR-135b-3p-mimic (sequence: 5′-ATGTAGGGCTAAAAGCCATGGG-3′), and scrambled control (sequence: 5'-TCGAGGGCGACTTAACCTTAGG-3') shRNAs (titers ~1 × 10^{13} GC/mL; VectorBuilder, Inc.). All AAV2 vectors co-expressed an mCherry reporter of transduction. Viruses (2 µl per eye) were injected intravitreally, avoiding injury to the lens, in 8-week-old mice, which were randomly assigned to experimental or control conditions, 2 weeks prior to ONC surgery. It is standard protocol to inject AAV2 two weeks prior to ONC, because this lead time allows sufficient time for transduction and expression of the shRNAs in RGCs at the time of ONC[6] (the timings of treatment and injury are shown in the experimental timeline figures). Transduction efficiency was approximately 30%, which is within a range of prior reports that also used intravitreal AAV2 to target the RGCs[2-4,6-8]. The RP-RGD was manufactured as a custom service by GenScript (Piscataway, NJ), as we previously described[9,10]. Immediately after ONC surgery, silicon oil-based emulsion containing RP-RGD (0.2 mg/ml in PBS) or control PBS were injected intravitreally (while allowing equivalent volume to leak-out upon needle withdrawal after 3 µl contents was slowly released into the eye), avoiding injury to the lens. Emulsion using silicon oil (Silikon 1000, 8065601187, Alcon) was prepared following the methods we previously described[9,10]. Cholera toxin subunit B (CTB) conjugated to Alexa Fluor 488 dye (C34775, ThermoFisher Scientific) was injected (1% in 3 µl PBS) intravitreally one day prior to sacrifice, at 2 or 6 weeks after ONC, in order to visualize the regenerating axons or their absence (although 2 µl of AlexaFluor-conjugated CTB solution is sufficient for labeling axons in the optic nerve, we added 1 µl extra in order to retain strong fluorescent signal intensity even at long distances through the optic chiasm and tract). Investigators performing the surgeries and injections were masked to the group identity until the end of the experiment.

Tissue processing and immunostaining. Standard histological procedures were used, as described previously[1-4,11]. Briefly, anesthetized mice were transcardially perfused with isotonic saline followed by 4% paraformaldehyde (PFA) at 2 or 6 weeks after ONC, the eyes, optic nerves, and optic chiasms were dissected and postfixed 2 hrs. The retinas were dissected-out from the eyes into PBS in 24-well plate at 4 °C. The optic nerves and chiasms were transferred to 30% sucrose overnight at 4 °C. Then, optic nerves and chiasms were embedded in OCT Tissue Tek Medium (Sakura Finetek) and frozen. Optic nerves and chiasms were cryosectioned longitudinally or horizontally, respectively, at 14 µm, and then mounted for imaging on coated glass slides. For analyzing RGC survival, resected (into PBS at 4 °C) free-floating retinas were immunostained in 24-well plate wells and, after making 4 symmetrical slits, flat-mounted on coated glass slides for imaging. For analyzing RGC transduction, the flattened retinas were embedded in OCT Tissue Tek Medium (Sakura Finetek), frozen, and cryosectioned at 14 µm horizontally (capturing the ganglion cell layer of the whole retina), and then immunostained and mounted on coated glass slides for imaging. For immunostaining, the tissues were blocked with appropriate sera, incubated overnight at 4 °C with primary antibody against βIII-Tubulin (1:500; rabbit polyclonal, Abcam, Ab18207), washed 3 times, incubated with appropriate fluorescent dye-conjugated secondary antibody (1:500; IgG H+L; Alexa Fluor, Thermo Fisher Scientific) overnight at 4 °C, washed 3 times again, and mounted for imaging.

Imaging and quantifications of RGC survival/axon regeneration. To visualize the regenerating axons or their absence after treating with the AAV2 vectors expressing miR-135a-1-3p-mimic, miR-135b-3p-mimic, or scrambled control, axonal tracer (Alexa Fluor 488-conjugated CTB 1% in 3 µl PBS) was intravitreally injected one day before animals were euthanized 2 or 6 weeks following ONC. Longitudinal sections of the optic nerve were examined for possible axon sparing[3]. No spared axons were found in control, and no evidence of axon sparing was found in experimental conditions (e.g., at 2 weeks after injury, no axons were found at the most distal from the injury region of the optic nerve). Regenerated axons (defined as continuous fibers, which are absent in controls and are discernible from background puncta and artefactual structures) were counted

manually using a fluorescent microscope (40x/1.2 C-Apochromat W; AxioObserver.Z1, Zeiss) in 4 longitudinal sections per optic nerve at 0.5 mm intervals at increasing distances from the injury site (identified by the abrupt disruption of the densely packed axons near the optic nerve head, as marked by a rhombus in Figures), and these values were used to estimate the total number of regenerating axons per nerve, as described[1-4]. For representative images, serial fields of view along the longitudinal optic nerve or through horizontal optic chiasm tissue cryosections were imaged as above; *z*-stacks with 5 planes at 0.5 µm intervals were deconvoluted, merged, and stitched (ZEN software, Zeiss). The edges of the tissue were optically trimmed (i.e., cropped-out) due to artefactual autofluorescence that is common at tissue edges. Then, processed images of 2 tissue sections from the same optic nerve were superimposed over each other and merged using Photoshop CS6 (Adobe), shown as representative images. Only 1 tissue section was used for horizontal optic chiasm representative images, because differences in size of optic chiasm's horizontal sections make it difficult to superimpose them in Adobe Photoshop CS6. RGC survival was quantified in retinal flatmounts' GCL as described[1-4,11], by immunostaining with an antibody to βIII-Tubulin (neuronal marker), taking advantage of the βIII-tubulin's selective expression in RGCs within the retina's GCL. ImageJ software Cell Counter Plugin was used to count βIII-Tubulin positive cells from images acquired (using a fluorescent microscope, 20x LD; Zeiss, AxioObserver.Z1, Zeiss) at 1-2 mm from the optic nerve head in four directions of the GCL, then averaged to estimate overall RGC survival per mm^2. For visualization of transduction, retinal regions randomly sampled from the horizontally cryosectioned flattened retinas' GCL immunostained for βIII-Tubulin were imaged, along with the mCherry reporter, using a confocal microscope (63x Oil; LSM800, Zeiss). Investigators performing the surgeries and quantifications were masked to the group identity by another researcher until the end of the experiment.

Primary neurons isolation, semi-quantitative PCR (sqPCR), and Sanger sequencing. Retinas were resected from both sexes of 5 mice of the respective ages: Embryonic day 18 (E18), postnatal day 5 (P5), or 10-12-week adult. RGCs were isolated from retinal single cell suspension by Thy1 immunopanning (CD90, MCA02R, Serotec) after depletion of macrophages (anti-mouse macrophage antibody, AIA31240, Accurate Chemical) and washing off the nonadherent cells, as previously described[7,8,12-14], with the following modifications for adult tissue: digestion in papain decreased to 10 minutes, amount of papain increased by 25%, only one macrophage depletion plate used for 20 minutes, amacrine cell depletion plate (VC1.1, C6680-100TST, Sigma) was used for 20 minutes, and Thy1 panning dish shaken more stringently, also as we previously described[8,15,16]. Motor cortex projection neurons were isolated from single cell suspension of resected motor cortex gray matter of P5 or 10-week-old mice of both sexes by Thy1 immunopanning after depletion of macrophages, as above. RNA was isolated from pelleted cells using the Quick-RNA Microprep Kit (ZR1050, Zymo Research). RNA concentration was measured with NanoPhotometer NP80 (Implen), and 100 ng of total RNA from each sample was used as input for library preparations according to the manufacturer's instructions, as follows. Equivalent concentrations and quantities of total RNA were used as input for mRNA cDNA library using iScript cDNA synthesis kit (Bio-Rad) or for small-RNA cDNA library using NEBNext Multiplex Small RNA Sample prep kit (E7330S, NEB). Spike-in control was used in preparation of small-RNA cDNA library for miRNA sqPCR following methods we described previously[8], as follows: 5.5 µl of total RNA (100 ng total RNA) was mixed with 0.5 µl of *ath-miR775* miRNA mimic (5'-UUCGAUGUCUAGCAGUGCCA-3'; at 0.625 ng/µl) concentration after 1:800 dilution from stock solution (10620310, custom RNA with phosphate 5' modification, ThermoFisher Scientific). Spike-in with *ath-miR775* miRNA mimic was used for normalization of expression across library replicates and timepoints/conditions for comparative analysis. All subsequent steps of small-RNA library prep were performed according to the manufacturer protocol (E7330S, NEB), through purification of the cDNA library using the Monarch PCR and DNA Cleanup Kit (T1030S, NEB). Libraries were eluted in 30 µl of nuclease-free water. For sqPCR, 2 µl of library was used as input in a 50 µl PCR reaction using Q5 Polymerase (M0491S, NEB). All samples were prepared using a single PCR master mix (buffer, water, universal reverse primer, dNTPs and polymerase). The universal reverse primer (5'-CAAGCAGAAGACGGCATACGAGA-3') is specific to the 3' adaptor from the NEB small-RNA library kit (E7330S), as we previously described and validated[8]. Then, 2 µl of template along with 2.5 µl of miRNA-specific primer (either for a target miR-135a-1-3p 5'-TATAGGGATTGGAGCCGTGG-3', or miR-135b-3p 5'-ATGTAGGGCTAAAAGCCATGGG-3') or spike-in control (*ath*-miR-775) were added to each sample, and PCR was run for 34 cycles on a BioRad T100 Thermal Cycler. After PCR, samples were electrophoresed on a 1.2% agarose TAE gel, along with DNA ladder. SYBR Safe DNA Gel Stain (S33102, Thermo Fisher Scientific) was used for visualization of bands in the gels, which were imaged using the BioRad Universal Hood II, and images were exported in TIFF format. Gel band intensities in the images were quantified using ImageJ software. Three biological replicates per condition were used. Intensity of the gel

band, matching the expected size of the amplified target miRNA with adaptors, was normalized to spike-in control band intensity, and then normalized by sum across replicates. Gel bands matching the expected size of the amplified target miRNA with adaptor were excised, sqPCR products were purified using the Zymoclean Gel DNA Recovery Kit (D4001, Zymo Research), and the purified products were Sanger-sequenced (QuintaraBio, Farmington, CT). The identified sequences were aligned to the genome (mm39) and blasted against the mouse miRbase database. For PCR of mRNA cDNA library, 1 µl of each sample was used as a template for 32-cycles PCR reaction using iQ SYBR Green Supermix with the iTaq DNA polymerase (Bio-Rad), performed on the iCycler iQ5 (Bio-Rad) with primers for the following genes: Tubb3 (forward: 5'-TAGACCCCAGCGGCAACTAT-3'; reverse: 5'-GTTCCAGGTTCCAAGTCCACC-3')[17]; Thy1 (forward: 5'-CTTGCAGGTGTCCCGAGGGC-3'; reverse: 5'-ATGGGATTCGCGCCCGAGAC-3')[18]; and Actb housekeeping control for which cDNA template was diluted 100x (forward: 5'-TGACGTTGACATCCGTAAAG-3'; reverse: 5'-GAGGAGCAATGATCTTGATCT-3')[19]. Standard no-template and no-RT controls were used. Samples (10 µl of RT-PCR products) were electrophoresed on a 1.5% agarose gel with TAE, along with 50 bp DNA ladder (NE3236S, NEB). SYBR Safe DNA Gel Stain (S33102, Thermo Fisher Scientific) was used for visualization of bands in the gels. Gel images were acquired using the BioRad Universal Hood II and exported in tiff format.

Primary neurons culture. P5 and P70 motor cortex neurons isolated by Thy1 immunopanning (as described in the previous section) were cultured in 48-well tissue culture plates (Falcon), coated with poly-D-lysine (10 mg/mL) and fibronectin (20 µg/mL; 1030FN01M, R&D Systems), in a defined medium which we described previously for RGC culture[9], with the exception that FGF2 (0.1 µg/mL; BT-FGFB, R&D Systems) was added, as adult neurons survive poorly in culture. At 5 DIC, cells were fixed for immunostaining with an antibody to βIII-Tubulin (1:500; mouse IgG2a, MMS-435P, BioLegend) and appropriate fluorescent dye-conjugated secondary antibodies (1:500; AlexaFluor, Thermo Fisher Scientific), and then imaged with a fluorescent microscope (20x LD; Zeiss, AxioObserver.Z1, Zeiss), using the methods we described previously[9].

RGC bulk-RNA-seq and scRNA-seq. All bulk small-RNA-seq and mRNA-seq samples from different age RGCs were purified by immunopanning for Thy1 (after immunopanning depletion of macrophages and amacrine cells, as described above). For bulk-mRNA-seq of adult RGCs transduced with AAV2 (~30% transduction efficiency) expressing miR-135a-1-3p-mimic, miR-135b-3p-mimic, or scramble-control shRNA, RGCs were Thy1-immunopanned from single cell retinal suspension (from 10 post-ONC retinas per condition) and FACS'ed for mCherry+ cells, as we described previously[8,16]. Approximately, 5,000 RGCs (per condition) were collected by FACS and RNA was isolated immediately using the Quick-RNA Microprep Kit (ZR1050, Zymo Research). Total RNA with RNA Integrity Number (RIN) ≥ 9 (by Bioanalyzer 2100 using the Nano 6000 kit, Agilent) was extracted using Direct-zol RNA MiniPrep kit (R2050, Zymo Research). For mRNA-seq, cDNA libraries were prepared using polyA-selected RNA (TruSeq RNA Library Prep Kit, Illumina), paired reads sequenced 100 bp from each end on HiSeq 2000 Sequencer (Illumina), passed QC filters, mapped to the mm39 genome and transcriptome by Hisat2, and analyzed by the Cufflinks package as we previously described[8,14,20], with the following modifications: Transcript abundance was determined by Cuffquant based on the mm39 genome and differential expression analyzed by CuffDiff using default parameters. For small-RNA-seq, approximately 200,000 of Thy1-immunopanned RGCs were collected (from each condition) and RNA was isolated immediately for preparing cDNA libraries using adaptors that ligate to the endogenously modified 5' and 3' ends of processed small-RNAs, thereby enriching for small-RNAs such as miRNAs, while excluding degraded RNA fragments, precursors, and other RNA species (TruSeq Small RNA Kit, Illumina). Small-RNA cDNA library products in the range of 140-160 base pairs (including 5' and 3' adapters and an insert corresponding to a small-RNA) were sequenced as paired reads 100 bp from each end on HiSeq 2000 Sequencer (Illumina), passed QC filters, and mapped to the mm39 genome and transcriptome, using Bowtie with default parameters. The resulting binary alignment map (BAM) files were filtered to retain only the full-length (i.e., limited by an miRNA size) reads with 100% alignment and then randomly subsampled, using Samtools, to a similar number of total reads in each sample for comparative analysis. The filtered reads' FASTQ were then realigned (with 1 mismatch allowed, multimapping disabled, and end-to-end Bowtie parameters) to a miRbase miRNA transcripts reference customized to remove the miR-135a-5p (in order for its respective reads to align to the miR-135b-5p with 1 mismatch allowed for the combined miR-135a/b-5p net expression) and quantified/analyzed using salmon packages[21] (mapping based mode) and DESeq2[22]. For alignments visualizations using IGV viewer (Broad Institute)[23] in Fig. 1C, the filtered reads were also realigned (with 1 mismatch allowed, multimapping enabled, and end-to-end Bowtie parameters) to a mm39 genome reference. For miR-135a/b-5p net reads alignment visualization, the miR-135a-1-5p locus shown in Fig.

1C also includes miR-135a-2-5p and miR-135b-5p reads that aligned to it. MiRNAs' TPM values (from the salmon quant.sf) for replicates were averaged in each age/condition, and shown on *x*-axis of the DESeq2 differential abundance plot in Fig. 1D. Previously generated by us and others RGC scRNA-seq datasets used in this study are available through the NCBI Gene Expression Omnibus (GEO) accession numbers provided in the Data Availability section below. Datasets were merged and normalized using Seurat v5.2.0[24,25]. RGC clusters assignments (in Figure S2H) were as described previously[16,26], and bubble plot (in Figure S2H) indicating RGC subtypes' normalized gene expression and proportion of RGCs per subtype expressing the gene was generated using the R package ggplot2[27].

miRNA targets prediction. miRNA target prediction analysis was performed on genes expressed in adult injured RGCs scRNA-seq dataset[26] at normalized expression (*NE*) of at least 0.5 *NE* (this minimal *NE* threshold was set in order to filter-out genes expressed at near-noise levels as they are unlikely to present axon regeneration-suppressing targets). The selected injured RGC genes were then cross-referenced to the dataset of transcript-specific bulk-mRNA-seq-profiled ONC-injured RGCs (which we generated as described below, GEO accession # GSE329865) to identify respective transcript isoforms that are expressed at least 0.5 *FPKM* in each of the 2 replicates. miRNA target prediction analysis was performed on 3' UTR sequences of these transcripts. The 3' UTRs of the selected annotated RGC transcripts were extracted using a custom Bash script along with the modules BEDtools 2.29.0, Bedops 2.4.35, and Gffread 0.12.7. Then, extracted 3' UTRs (**Table S1**) and fasta files of the miRNA sequences were used as inputs for miRanda 3.3a with default parameters[28,29]. The output from miRanda was parsed using Perl to obtain a table of predicted targets and their associated miRanda target prediction scores (Figure S2A-G).

Gene annotation/ontology enrichment analysis was performed on genes differentially expressed (Cuffdiff *q*-value < 0.05) in bulk-mRNA-seq-profiled injured RGCs after either miR-135a-1-3p-mimic or miR-135b-3p-mimic treatment relative to scrambled-control shRNA treatment, that were further filtered based on at least 2 log2FC regulation relative to scrambled-control shRNA-treated uninjured RGCs and the higher expression condition expressing the gene at least 5 FPKM. Due to potential contamination with non-RGC retinal cell-types, only genes established to be expressed (at least 0.01 *NE*) in any RGC state (e.g., embryonic, postnatal, adult, injured, or regenerative, based on our and others' prior scRNA-seq studies[14,30-32]) were retained for analysis. Gene annotation/ontology enrichment analysis was performed using the GO:BP terms database and the R packages *gprofiler2*, *enrichplot*, and *clusterProfiler*[33-37]. All genes expressed in adult uninjured RGCs (FPKM ≥ 0.1) based on the dataset we generated previously[8] were used as background. DEG-associated GO:BP terms were identified using *gprofiler2*'s gost function with the g:SCS method for multiple testing correction and a significance threshold of $p < 0.05$. For each GO term, fold-enrichment was calculated as the proportion of query genes associated with the GO term divided by the proportion of background genes associated with the GO term. GO terms with log2 fold-enrichment > 0.8 were retained, and semantically redundant terms were removed using the simplify function ('cutoff = 0.8'). Genes comprising significantly enriched annotation/ontology terms were analyzed by a gene network plot using the *enrichplot* R package cnetplot function[35,37].

Experimental design and statistical analyses. All tissue processing, quantification, and data analysis were done masked throughout the study. Sample sizes were based on accepted standards in the literature and our prior experiences. Sample size (*n*) represents total number of biological replicates in each condition. All experiments included appropriate controls. No cases were excluded in our data analysis, although a few animals that developed a cataract in the injured eye were excluded from the study, and their tissues were not processed. The data are presented as means (SEM) and was analyzed (as specified in the respective Figure legends), where appropriate, by independent samples *t*-test 2-tailed, or one-way ANOVA with or without Repeated Measures, and a posthoc LSD test (SPSS), as indicated. All differences were considered significant at $p < 0.05$.

Data availability. RGC bulk small-RNA-seq processed raw reads and data for miRNA 135 family miRNA loci analyzed in this study are available through the NCBI Sequence Read Archive (SRA) under accession # PRJNA1483696. Bulk-mRNA-seq processed raw reads and data for injured adult RGCs treated with miR-135a-1-3p-mimic or miR-135b-3p-mimic are available through the NCBI GEO under accession # GSE337069 and for treated with scramble-control shRNA under accession # GSE329865[38]. The adult uninjured RGC bulk-mRNA-seq dataset used in this study, which we previously generated, is available through the NCBI GEO under accession # GSE252517[8]. RGC scRNA-seq datasets used in this study, which were previously generated by us and others, are available through the NCBI GEO accession # GSE122466[30], GSE325128[32], GSE115404[14], GSE137400[26], and GSE202155[31].

Horizontal cryosections of the optic chiasm with adjoint optic nerves/tracts attached, at 6 wks post-ONC

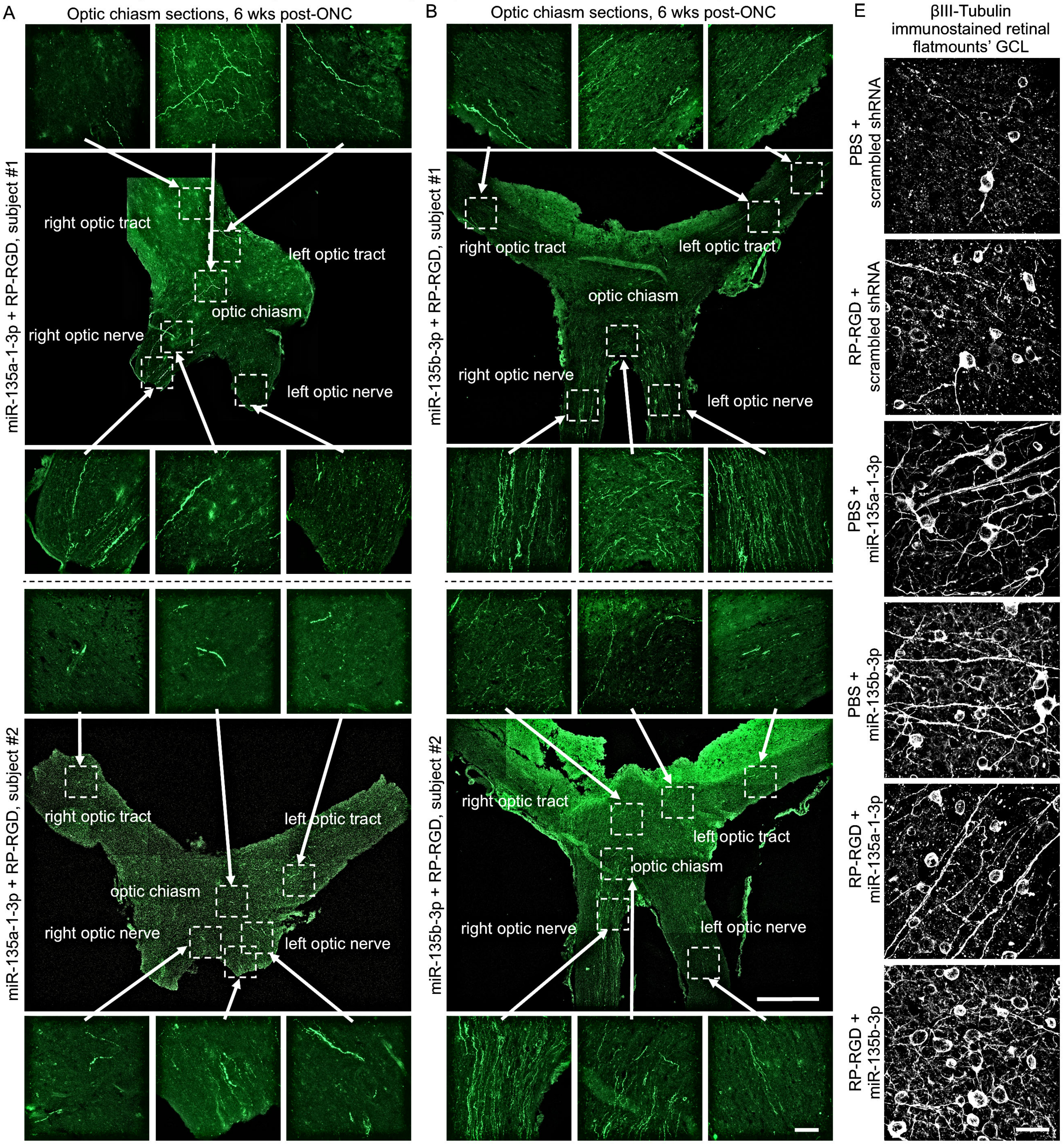

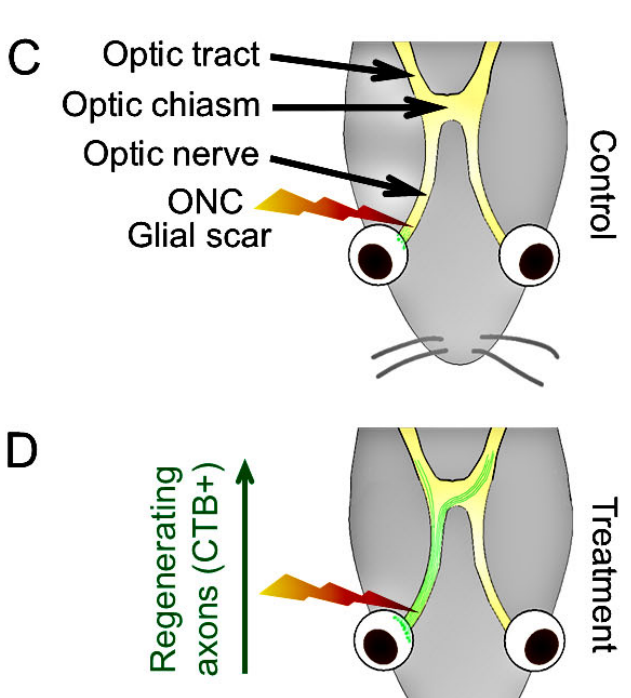

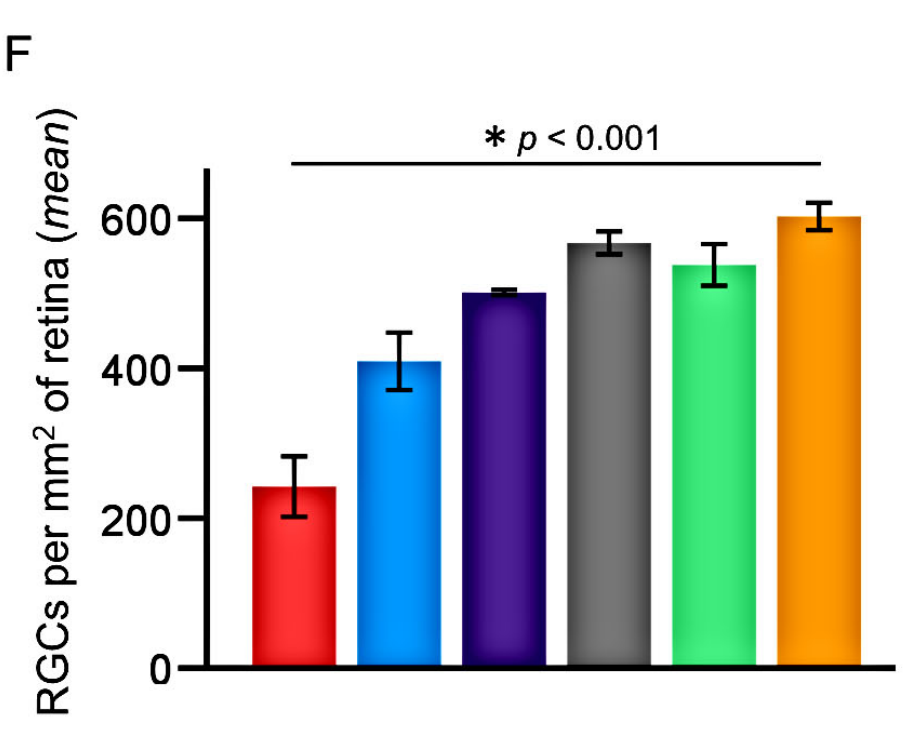

G

	Control shRNA + RP-RGD	miR-135a-1-3p + PBS	miR-135a-1-3p + RP-RGD	miR-135b-3p + PBS	miR-135b-3p + RP-RGD
Control shRNA + PBS	0.001 *	0.001 *	0.001 *	0.001 *	0.001 *
Control shRNA + RP-RGD		*N.S.*	0.002 *	0.010 *	0.001 *
miR-135a-1-3p + PBS			*N.S.*	*N.S.*	0.054 *
miR-135a-1-3p + RP-RGD				*N.S.*	*N.S.*
miR-135b-3p + PBS					*N.S.*

FIGURE S1

Figure S1. Axons regenerating in response to AAV2 miR-135a-1-3p or miR-135b-3p mimics co-treated with RP-RGD can reach the optic tract by 6 weeks post-injury.

(**A-B**) Experimental timeline shown in Fig. 3A. Representative images of horizontal cryosections of the optic chiasm with adjoint optic nerves/tracts segments attached, as marked, from the animals bilaterally injured and treated as marked. Images from two different animals with most regenerating axons detected by 6 weeks post-ONC are shown for co-treatments with RP-RGD and AAV2 miR-135a-1-3p (*A*) or miR-135b-3p (*B*). Insets: Representative CTB-labeled axons detected in segments of the central visual pathway (outlined by dashed boxes in *A-B*), as marked. There were no axons detected in the control condition (not shown), consistent with that no regenerating axons were detected even in the preceding segments of the central visual pathway, as absence of axons is apparent in the optic nerves from control conditions shown in Fig. 3. Scale bars, 500 μm (main panels), 20 μm (insets).

(**C-D**) To assist in the interpretation of the histological images, a schematic of failed axon regeneration in control shows the central visual pathway segments through the optic tract, along with ONC site, as marked (*C*), and a representation of axon regeneration reaching the optic tract achieved by the co-treatments at 6 weeks post-ONC is shown for comparison to control (*D*).

(**E-G**) Representative images of the retinal flatmounts' GCL immunostained for an RGC marker βIII-Tubulin at 6 weeks post-ONC, from the conditions pre-treated with AAV2 expressing miR-135a-1-3p-mimic, miR-135b-3p-mimic, or scrambled-control shRNA, and co-treated with RP-RGD or vehicle, as marked. Scale bar, 20 μm (*E*). Quantitation of RGC (βIII-Tubulin+ cells) survival in retinal flatmounts at 6 weeks post-ONC, from the treatment conditions as marked (*F*). Data analyzed using ANOVA, overall $F = 19.95$, $p < 0.001$, with *p*-values of pairwise comparisons determined by posthoc LSD shown in the table, and significant differences ($p < 0.05$) indicated by an asterisk * (*G*). Mean (SEM) shown; $n = 4$ cases per group.

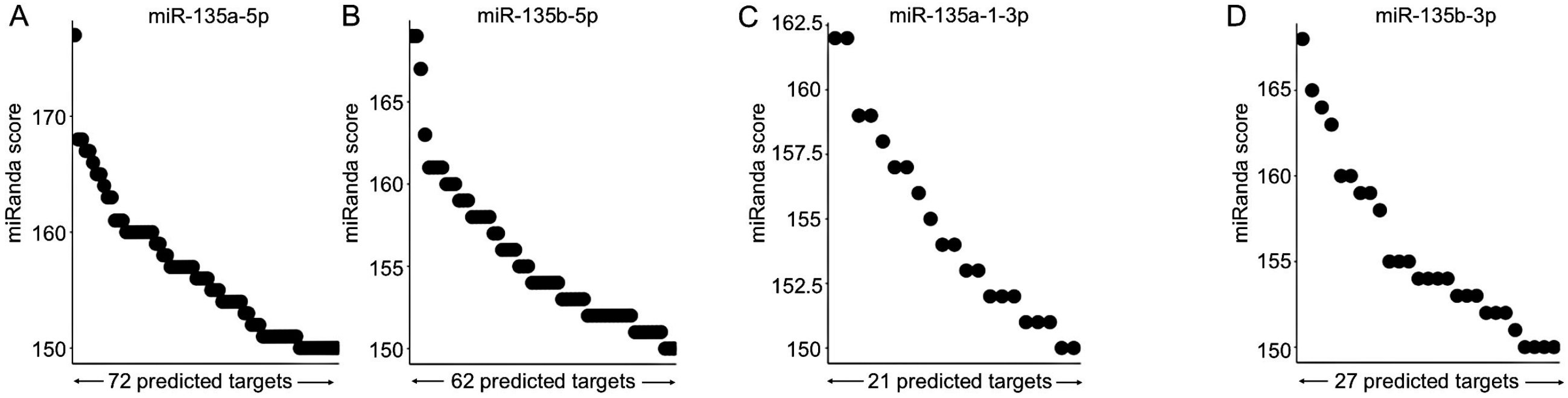

E

Gene	Transcript ID	miR-135a-5p Score	miR-135b-5p Score
Pbx1	ENSMUST00000176540	177	169
Zfp385b	ENSMUST00000111830	168	160
Eif4g2	ENSMUST00000162415	167	159
Kitl	ENSMUST00000105283	167	167
Hdac9	ENSMUST00000209902	166	158
Anks1b	ENSMUST00000182356	165	169
Robo2	ENSMUST00000117785	165	157
Klf4	ENSMUST00000107619	164	156
Rora	ENSMUST00000034766	163	155
Zwint	ENSMUST00000105431	163	155
Cpne8	ENSMUST00000088649	161	161
Rps20	ENSMUST00000138502	161	161
Trpm3	ENSMUST00000237357	161	161
Akap6	ENSMUST00000219786	160	152
Ank3	ENSMUST00000182665	160	152
Hcn1	ENSMUST00000006991	160	152
Itm2b	ENSMUST00000022704	160	160
Pde10a	ENSMUST00000115717	160	152
App	ENSMUST00000227723	159	163
Srgap1	ENSMUST00000020322	159	151
Snrpn	ENSMUST00000059305	158	158
Epha5	ENSMUST00000053733	157	150
Tnik	ENSMUST00000159236	157	161
Ywhae	ENSMUST00000067664	157	157
Gpr158	ENSMUST00000055946	156	152
Rpl5	ENSMUST00000082223	156	156
Strbp	ENSMUST00000028279	156	152
Fam171b	ENSMUST00000051454	155	151
Nrxn3	ENSMUST00000110130	155	159
Ext1	ENSMUST00000077273	154	154
H3f3a	ENSMUST00000161308	154	154
Mmp16	ENSMUST00000029881	154	158
Pde4b	ENSMUST00000106907	154	154
Nrg1	ENSMUST00000207470	152	156
Ppfia2	ENSMUST00000217854	152	156
B3galt1	ENSMUST00000112346	151	151
Zfand5	ENSMUST00000237651	151	155
Tcf25	ENSMUST00000211932	150	154

F

Gene	Transcript ID	miR-135a-1-3p Score
Kcnma1	ENSMUST00000145596	162
Zbtb20	ENSMUST00000114694	162
Dcc	ENSMUST00000114943	159
Pbx1	ENSMUST00000176540	159
Msi2	ENSMUST00000107909	158
Map2	ENSMUST00000024639	157
Eif4g2	ENSMUST00000162415	156
Pcdh11x	ENSMUST00000113358	155
Gpr158	ENSMUST00000055946	154
Inpp4b	ENSMUST00000172031	154
Tmtc2	ENSMUST00000061506	153
Il1rapl1	ENSMUST00000113966	153
Ywhaq	ENSMUST00000135088	152
Pcdh15	ENSMUST00000191854	152
Tafa1	ENSMUST00000075080	151
Kcnh7	ENSMUST00000075052	151
Robo2	ENSMUST00000117785	150
Dscam	ENSMUST00000056102	150

G

Gene	Transcript ID	miR-135b-3p Score
Fgf14	ENSMUST00000095529	168
Gabrg3	ENSMUST00000068911	165
Pten	ENSMUST00000249247	164
Zbtb20	ENSMUST00000114694	163
Celf2	ENSMUST00000170438	160
Eif4g2	ENSMUST00000162415	159
Kcnma1	ENSMUST00000145596	159
Tenm4	ENSMUST00000107166	158
Anks1b	ENSMUST00000182907	155
Gpx4	ENSMUST00000105372	155
Cdh12	ENSMUST00000075132	154
Camk2d	ENSMUST00000200171	154
Khdrbs2	ENSMUST00000188257	153
Neto1	ENSMUST00000058829	153
Atp5g1	ENSMUST00000090541	153
Magi1	ENSMUST00000089317	152
Map1b	ENSMUST00000064762	151
Zfand5	ENSMUST00000237651	150
Nnat	ENSMUST00000153739	150

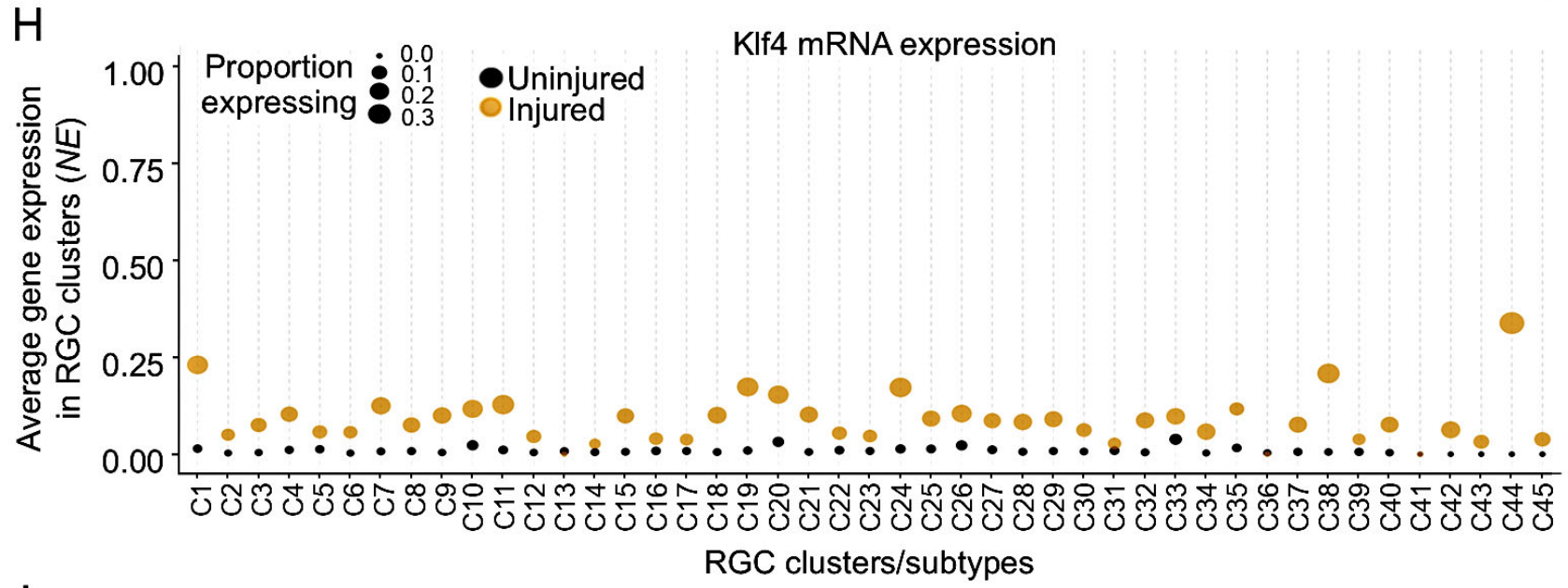

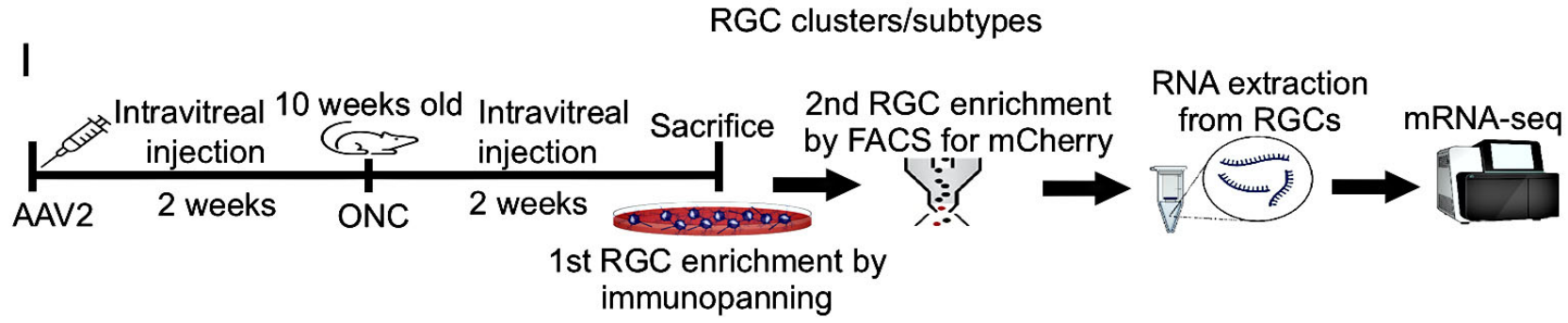

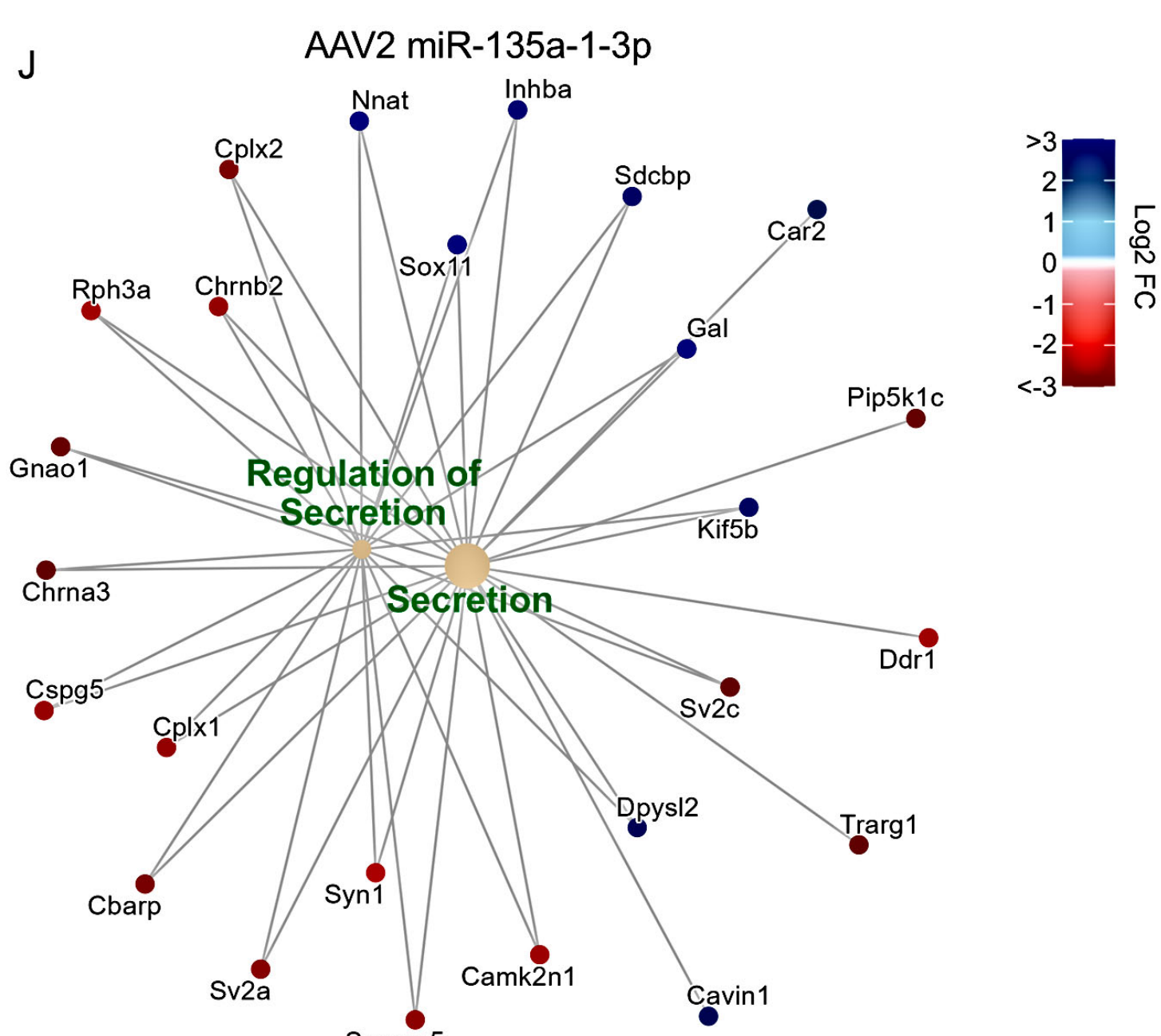

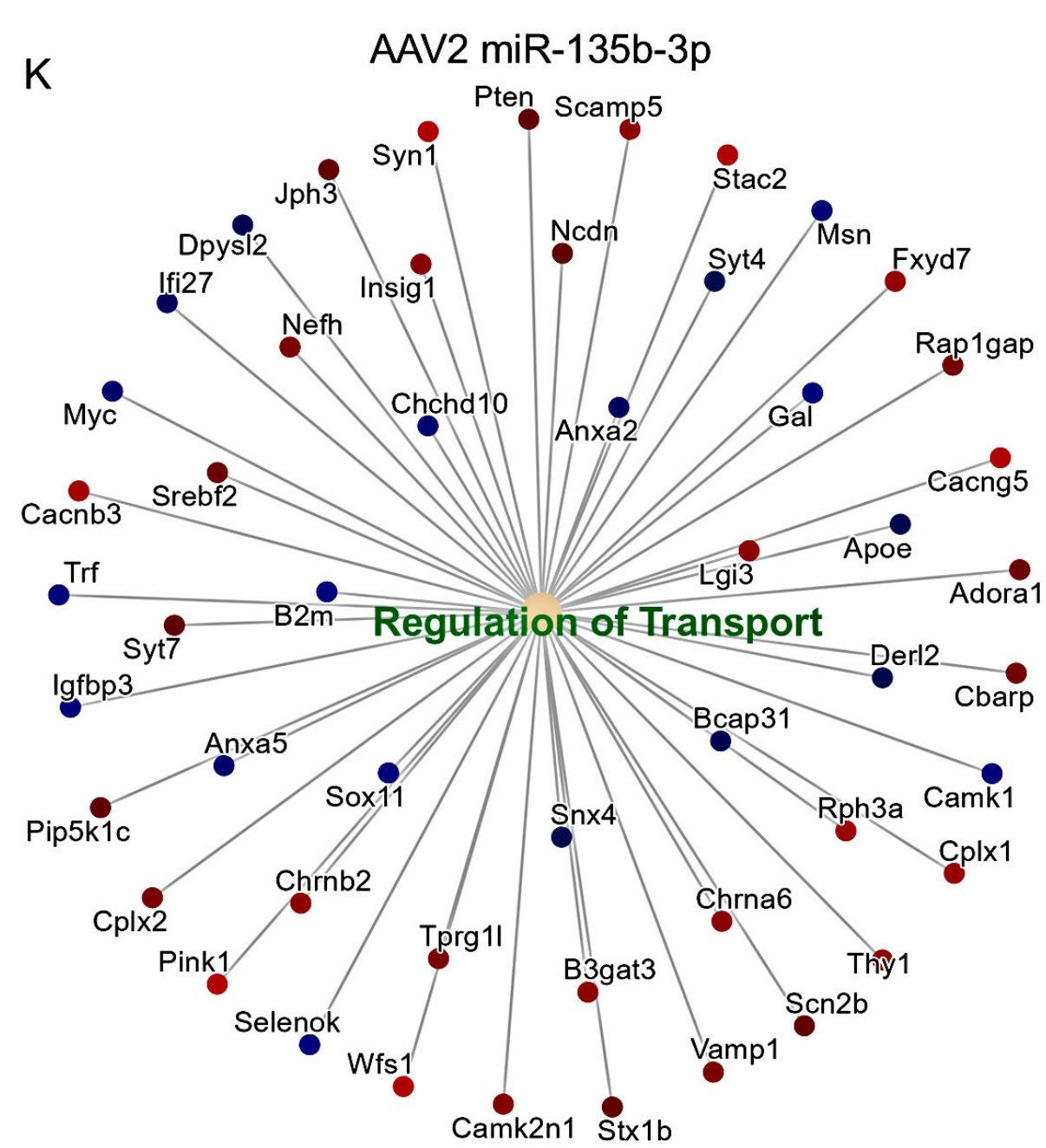

FIGURE S2

Figure S2. miR-135 family miRNAs' predicted targets and genes networks affected by miR-135a-1-3p and miR-135b-3p mimics in injured RGCs.

(**A-D**) Plot of mRNAs (*n*) expressed in injured RGCs vs. respective miRNA's predicted miRanda targeting score, shown for miR-135a-5p (*A*), miR-135b-5p (*B*), miR-135a-1-3p (*C*), and miR-135b-3p (*D*), ordered by prediction score (with higher score indicating higher targeting probability); see Methods.

(**E-G**) The top predicted (score ≥ 150) target genes, along with transcript IDs (one per gene with the highest score shown) and miRanda scores, are shown for the miR-135 family members, as marked. Majority of miR-135a-5p and the differing by 1 bp miR-135b-5p targets are shared, with miR-135a-5p targeting 81% of genes targeted by miR-135b-5p and miR-135b-5p targeting 70% of genes targeted by miR-135a-5p (*E*). Targets shared between the 3p miRNAs are highlighted in blue (*F-G*), with a minor overlapping subset also highlighted for 5p miRNAs. Klf4, highlighted in red, is a predicted target of miR-135a/b-5p, but not of miR-135b-3p or miR-135a-1-3p (see Methods).

(**H**) Bubble plot of Klf4 expression in scRNA-seq RGC datasets[26] suggest only modest (bordering noise) Klf4 levels in the injured (2-wks post-ONC) RGC subtypes. Bubble size scalebar indicates the cluster proportion of RGCs with Klf4 detected at *NE* > 0. *NE* = normalized expression.

(**I**) Experimental timeline: 8-week-old mice were pre-treated with AAV2 expressing miR-135a-1-3p-mimic, miR-135b-3p-mimic, or scrambled-control shRNA, and co-expressing mCherry reporter. ONC injury was performed 2 weeks later, and mCherry+ RGCs were isolated 2 weeks post-ONC for bulk-mRNA-seq analysis. A different, uninjured group was processed similarly (see Methods).

(**J-K**) GO-based gene-concept network plots of the biological processes' terms significantly enriched in the differentially expressed genes in miR-135a-1-3p (*J*) and miR-135b-3p (*K*) treated injured RGCs relative to scrambled-control shRNA-treated injured and uninjured RGCs (see Methods). GO terms are shown as brown circles (i.e., nodes), with circle size (per scale on the side) indicating the number of genes within that node. The color-coded scale bar indicates each gene's log2 fold-change (FC) in expression between the miR-135a-1-3p or miR-135b-3p and scrambled-control shRNA treated injured RGCs.

SUPPLEMENTAL DATA LEGENDS

Table S1. 3’ UTR sequences in FASTA format for transcripts isoforms determined, based on RNA-seq analysis, as expressed in ONC-injured RGCs. Refer to the main text and methods for technical details.

Table S2. List of differentially expressed genes (DEGs) with expression and fold-change (FC) values. Log2 FC (log2FC) between the average expression in miR-135a-1-3p or miR-135b-3p mimic-treated injured RGCs and either scrambled-control shRNA-treated injured or uninjured RGCs is shown. Significantly (CuffDiff *q*-value < 0.05) differentially expressed genes (DEGs) between scrambled-control shRNA-treated injured RGCs, that are also at least 2 log2FC relative to uninjured RGCs and with ≥ 5 FPKM in the higher-expressing condition, are shown with the columns annotated as indicated. Refer to the main text and methods for technical details.

SUPPLEMENTAL REFERENCES

1. de Lima, S., Koriyama, Y., Kurimoto, T., Oliveira, J.T., Yin, Y., Li, Y., Gilbert, H.Y., Fagiolini, M., Martinez, A.M., and Benowitz, L. (2012). Full-length axon regeneration in the adult mouse optic nerve and partial recovery of simple visual behaviors. Proc Natl Acad Sci U S A *109*, 9149-9154. 10.1073/pnas.1119449109.
2. Trakhtenberg, E.F., Li, Y., Feng, Q., Tso, J., Rosenberg, P.A., Goldberg, J.L., and Benowitz, L.I. (2018). Zinc chelation and Klf9 knockdown cooperatively promote axon regeneration after optic nerve injury. Exp Neurol *300*, 22-29. 10.1016/j.expneurol.2017.10.025.
3. Kim, J., Sajid, M.S., and Trakhtenberg, E.F. (2018). The extent of extra-axonal tissue damage determines the levels of CSPG upregulation and the success of experimental axon regeneration in the CNS. Sci Rep *8*, 9839. 10.1038/s41598-018-28209-z.
4. Lukomska, A., Kim, J., Rheaume, B.A., Xing, J., Hoyt, A., Lecky, E., Steidl, T., and Trakhtenberg, E.F. (2021). Developmentally upregulated Transcriptional Elongation Factor A like 3 suppresses axon regeneration after optic nerve injury. Neurosci Lett, 136260. 10.1016/j.neulet.2021.136260.
5. Lukomska, A., Frost, M.P., Theune, W.C., Xing, J., Gupta, M., and Trakhtenberg, E.F. (2024). Nfe2l3 promotes neuroprotection and long-distance axon regeneration after injury in vivo. Exp Neurol, 114741. 10.1016/j.expneurol.2024.114741.
6. Yungher, B.J., Luo, X., Salgueiro, Y., Blackmore, M.G., and Park, K.K. (2015). Viral vector-based improvement of optic nerve regeneration: characterization of individual axons' growth patterns and synaptogenesis in a visual target. Gene Ther *22*, 811-821. 10.1038/gt.2015.51.
7. Trakhtenberg, E.F., Wang, Y., Morkin, M.I., Fernandez, S.G., Mlacker, G.M., Shechter, J.M., Liu, X., Patel, K.H., Lapins, A., Yang, S., Dombrowski, S.M., et al. (2014). Regulating Set-β's Subcellular Localization Toggles Its Function between Inhibiting and Promoting Axon Growth and Regeneration. J Neurosci *34*, 7361-7374. 10.1523/JNEUROSCI.3658-13.2014.
8. Lukomska, A., Theune, W.C., Frost, M.P., Xing, J., Kearney, A., and Trakhtenberg, E.F. (2024). Upregulation of developmentally-downregulated miR-1247-5p promotes neuroprotection and axon regeneration in vivo. Neurosci Lett *823*, 137662. 10.1016/j.neulet.2024.137662.
9. Lukomska, A., Rheaume, B.A., Frost, M.P., Theune, W.C., Xing, J., Damania, A., and Trakhtenberg, E.F. (2024). Augmenting fibronectin levels in injured adult CNS promotes axon regeneration in vivo. Exp Neurol *379*, 114877. 10.1016/j.expneurol.2024.114877.
10. Lukomska, A., Frost, M.P., Brady, J., Kearney, A., Balaji, A.J., D'Souza, C., and Trakhtenberg, E.F. (2026). Experimental upregulation of Lancl1 promotes axon regeneration after optic nerve injury in vivo. Exp Neurol *402*, 115789. 10.1016/j.expneurol.2026.115789.
11. Kurimoto, T., Yin, Y., Omura, K., Gilbert, H.Y., Kim, D., Cen, L.P., Moko, L., Kügler, S., and Benowitz, L.I. (2010). Long-distance axon regeneration in the mature optic nerve: contributions of oncomodulin, cAMP, and pten gene deletion. J Neurosci *30*, 15654-15663. 10.1523/JNEUROSCI.4340-10.2010.
12. Meyer-Franke, A., Kaplan, M.R., Pfrieger, F.W., and Barres, B.A. (1995). Characterization of the signaling interactions that promote the survival and growth of developing retinal ganglion cells in culture. Neuron *15*, 805-819.
13. Trakhtenberg, E.F., Pita-Thomas, W., Fernandez, S.G., Patel, K.H., Venugopalan, P., Shechter, J.M., Morkin, M.I., Galvao, J., Liu, X., Dombrowski, S.M., and Goldberg, J.L. (2016). Serotonin receptor 2C regulates neurite growth and is necessary for normal retinal processing of visual information. Dev Neurobiol. 10.1002/dneu.22391.
14. Rheaume, B.A., Jereen, A., Bolisetty, M., Sajid, M.S., Yang, Y., Renna, K., Sun, L., Robson, P., and Trakhtenberg, E.F. (2018). Single cell transcriptome profiling of retinal ganglion cells identifies cellular subtypes. Nat Commun *9*, 2759. 10.1038/s41467-018-05134-3.

15. Xing, J., Lukomska, A., Rheaume, B.A., Kim, J., Sajid, M.S., Damania, A., and Trakhtenberg, E.F. (2023). Post-injury born oligodendrocytes incorporate into the glial scar and contribute to the inhibition of axon regeneration. Development. 10.1242/dev.201311.

16. Rheaume, B.A., Xing, J., Lukomska, A., Theune, W.C., Damania, A., Sjogren, G., and Trakhtenberg, E.F. (2023). Pten inhibition dedifferentiates long-distance axon-regenerating intrinsically photosensitive retinal ganglion cells and upregulates mitochondria-associated Dynlt1a and Lars2. Development *150*. 10.1242/dev.201644.

17. Matsuda, T., Irie, T., Katsurabayashi, S., Hayashi, Y., Nagai, T., Hamazaki, N., Adefuin, A.M.D., Miura, F., Ito, T., Kimura, H., Shirahige, K., et al. (2019). Pioneer Factor NeuroD1 Rearranges Transcriptional and Epigenetic Profiles to Execute Microglia-Neuron Conversion. Neuron *101*, 472-485.e477. 10.1016/j.neuron.2018.12.010.

18. Li, Y., Schlamp, C.L., and Nickells, R.W. (1999). Experimental induction of retinal ganglion cell death in adult mice. Invest Ophthalmol Vis Sci *40*, 1004-1008.

19. Ho, K.H., and Patrizi, A. (2021). Assessment of common housekeeping genes as reference for gene expression studies using RT-qPCR in mouse choroid plexus. Sci Rep *11*, 3278. 10.1038/s41598-021-82800-5.

20. Trakhtenberg, E.F., Pho, N., Holton, K.M., Chittenden, T.W., Goldberg, J.L., and Dong, L. (2016). Cell types differ in global coordination of splicing and proportion of highly expressed genes. Sci Rep *6*, 32249. 10.1038/srep32249.

21. Patro, R., Duggal, G., Love, M.I., Irizarry, R.A., and Kingsford, C. (2017). Salmon provides fast and bias-aware quantification of transcript expression. Nat Methods *14*, 417-419. 10.1038/nmeth.4197.

22. Love, M.I., Huber, W., and Anders, S. (2014). Moderated estimation of fold change and dispersion for RNA-seq data with DESeq2. Genome Biol *15*, 550. 10.1186/s13059-014-0550-8.

23. Robinson, J.T., Thorvaldsdóttir, H., Winckler, W., Guttman, M., Lander, E.S., Getz, G., and Mesirov, J.P. (2011). Integrative genomics viewer. Nat Biotechnol *29*, 24-26. 10.1038/nbt.1754.

24. Stuart, T., Butler, A., Hoffman, P., Hafemeister, C., Papalexi, E., Mauck, W.M., Hao, Y., Stoeckius, M., Smibert, P., and Satija, R. (2019). Comprehensive Integration of Single-Cell Data. Cell *177*, 1888-1902.e1821. 10.1016/j.cell.2019.05.031.

25. Hao, Y., Hao, S., Andersen-Nissen, E., Mauck, W.M., Zheng, S., Butler, A., Lee, M.J., Wilk, A.J., Darby, C., Zager, M., Hoffman, P., et al. (2021). Integrated analysis of multimodal single-cell data. Cell *184*, 3573-3587.e3529. 10.1016/j.cell.2021.04.048.

26. Tran, N.M., Shekhar, K., Whitney, I.E., Jacobi, A., Benhar, I., Hong, G., Yan, W., Adiconis, X., Arnold, M.E., Lee, J.M., Levin, J.Z., et al. (2019). Single-Cell Profiles of Retinal Ganglion Cells Differing in Resilience to Injury Reveal Neuroprotective Genes. Neuron *104*, 1039-1055.e1012. 10.1016/j.neuron.2019.11.006.

27. Wickham, H. (2009). ggplot2: Elegant Graphics for Data Analysis (New York, NY: Springer).

28. Enright, A.J., John, B., Gaul, U., Tuschl, T., Sander, C., and Marks, D.S. (2003). MicroRNA targets in Drosophila. Genome Biol *5*, R1. 10.1186/gb-2003-5-1-r1.

29. John, B., Enright, A.J., Aravin, A., Tuschl, T., Sander, C., and Marks, D.S. (2004). Human MicroRNA targets. PLoS Biol *2*, e363. 10.1371/journal.pbio.0020363.

30. Lo Giudice, Q., Leleu, M., La Manno, G., and Fabre, P.J. (2019). Single-cell transcriptional logic of cell-fate specification and axon guidance in early-born retinal neurons. Development *146*. 10.1242/dev.178103.

31. Jacobi, A., Tran, N.M., Yan, W., Benhar, I., Tian, F., Schaffer, R., He, Z., and Sanes, J.R. (2022). Overlapping transcriptional programs promote survival and axonal regeneration of injured retinal ganglion cells. Neuron *110*, 2625-2645.e2627. 10.1016/j.neuron.2022.06.002.

32. Kearney, A., Lukomska, A., Brady, J., Damania, A., Gupta, M., and Trakhtenberg, E.F. (2026). Vacuolar ATPase subunit Atp6v0c transgene promotes neuroprotection and long-distance axon regeneration in injured retinal ganglion neurons. Mol Ther Nucleic Acids *37*, 102922. 10.1016/j.omtn.2026.102922.

33. Ashburner, M., Ball, C.A., Blake, J.A., Botstein, D., Butler, H., Cherry, J.M., Davis, A.P., Dolinski, K., Dwight, S.S., Eppig, J.T., Harris, M.A., et al. (2000). Gene ontology: tool for the unification of biology. The Gene Ontology Consortium. Nat Genet *25*, 25-29. 10.1038/75556.

34. Aleksander, S.A., Balhoff, J., Carbon, S., Cherry, J.M., Drabkin, H.J., Ebert, D., Feuermann, M., Gaudet, P., Harris, N.L., Hill, D.P., Lee, R., et al. (2023). The Gene Ontology knowledgebase in 2023. Genetics *224*. 10.1093/genetics/iyad031.

35. Yu, G. (2025). enrichplot: Visualization of Functional Enrichment Result. Bioconductor *v.1.28.4*. 10.18129/B9.bioc.enrichplot.

36. Kolberg, L., Raudvere, U., Kuzmin, I., Vilo, J., and Peterson, H. (2020). gprofiler2 -- an R package for gene list functional enrichment analysis and namespace conversion toolset g:Profiler. F1000Res *9*. 10.12688/f1000research.24956.2.

37. Wu, T., Hu, E., Xu, S., Chen, M., Guo, P., Dai, Z., Feng, T., Zhou, L., Tang, W., Zhan, L., Fu, X., et al. (2021). clusterProfiler 4.0: A universal enrichment tool for interpreting omics data. Innovation (Camb) *2*, 100141. 10.1016/j.xinn.2021.100141.

38. D'souza, C., Lukomska, A., Balaji, A.J., Brady, J., and Trakhtenberg, E.F. (2026). Targeting neurodevelopmental miR132-3p promotes neuroprotection and axon regeneration after optic nerve injury in mice. Brain Res *1889*, 150450. 10.1016/j.brainres.2026.150450.
