# Supplementary material for: Neurodevelopmental miR-135a-1-3p and miR-135b-3p promote long-distance axon regeneration after injury in the central visual pathway
Source: Mol Ther Adv. 2026 Jul 30;34(3):201826. doi: 10.1016/j.omta.2026.201826 (PMC13507755; doi:10.1016/j.omta.2026.201826)
Supplement: Supplementary file 2 [file mmc4.pdf]

Molecular Therapy
Advances
Brief report

Neurodevelopmental miR-135a-1-3p and miR-135b-3p promote long-distance axon regeneration after injury in the central visual pathway

Jacob H. Brady,[1,2] Agnieszka Lukomska,[1,2] Arnav J. Balaji,[1] Clyde D'Souza,[1] and Ephraim F. Trakhtenberg[1]

[1]Department of Neuroscience, University of Connecticut School of Medicine, 263 Farmington Ave, Farmington, CT 06030, USA

Micro-RNAs (miRNAs) play roles in the mammalian central nervous system (CNS) development and disease but the functions of specific miRNA families' members in defined neuronal types are poorly understood. Here, we characterized the miR-135 family members in the mouse CNS retinal ganglion cell (RGC) and cortical projection neurons development, and tested whether reverting expression toward embryonic levels for the identified developmentally regulated miR-135 family members may reactivate the intrinsic axon growth capacity following axonal injury in the adult CNS. We found that two-out-of-five miR-135 family members, miR-135a-1-3p and miR-135b-3p, were developmentally downregulated, with further downregulation after axonal injury, in neurons. Importantly, RGC-targeted mimics of miR-135a-1-3p and miR-135b-3p promoted neuroprotection and axon regeneration following optic nerve injury, with axons regrowing through a segment of the central visual pathway when co-treated with an axon growth-supporting small fibronectin-based recombinant protein. Finally, transcriptomic profiling of the treated RGCs showed that genes involved in neurodevelopment/neuroprotection were embedded in the gene networks affected by the treatments. Thus, miR-135a-1-3p and miR-135b-3p are developmentally downregulated in the mammalian CNS projection neurons and present options for translation toward CNS injury treatments.

INTRODUCTION

The failure of central nervous system (CNS) projection neurons to regenerate axons over long distances after white matter injury to the brain, spinal cord, or optic nerve/tract results in permanent disabilities.[1–9] Like other mammalian CNS projection neurons, retinal ganglion cells (RGCs) do not spontaneously regenerate axons injured in the optic nerve crush (ONC) model of traumatic optic neuropathy (TON).[10–12] Some injured neurons at least partially revert their transcriptomes toward an embryonic cell state[13–15] in a failed attempt to fully reactivate intrinsic axon growth mechanism (which was inactivated during maturation).[16] However, several developmentally regulated mRNAs were found to control the developmental decline[17,18] in intrinsic capacity of RGCs (and other CNS projection neurons[13]) to grow axons, and targeting these genes promoted axon regeneration after injury.[14,19–25] MicroRNAs (miRNAs) were also found to regulate axon growth and regeneration.[26–39] These advances highlight the need for studying the specific miRNA family members' roles in axon growth of defined neuronal types, which we pursued in these studies. MiRNAs are annotated based on their sequences[40] and could concurrently target multiple mRNAs in the same cell to regulate cellular processes.[40,41] miRNA families include miRNAs' 5p and 3p arms (i.e., two miRNAs derived from a single pre-miRNA precursor, where one arm could become dominant or both arms could regulate different mRNAs), as well as miRNAs expressed from different genomic loci with homologous seed regions but varying flanking sequences; each member may target different mRNAs, unless the sequences are identical, in which case they are termed "multicopy."[42–44] Our exploratory small-RNA-seq studies identified an association of the miR-135 family with RGC development (see data below). Because a multicopy member of this family (see below) targets the Klf4 transcription factor,[22] a developmental suppressor of axon growth, to promote axon regeneration,[39] we prioritized the previously unstudied and biologically distinct miR-135 family members (that do not target Klf4) for investigation in RGC and cortical neuron development and axon regeneration after ONC injury.

The miR-135 family is comprised of five miRNA sequences which originate from Chr1, Chr9, and Chr10, as follows: the miR-135a′s 5p arms from Chr9 and Chr10 are the miR-135a-5p multicopy which is near-identical to miR-135b-5p from Chr1 that differs by only one base pair (bp), whereas the 3p arms differ from the 5p arms by almost 50%, with miR-135a-1-3p, miR-135a-2-3p, and miR-135b-3p originating from Chr9, Chr10, and Chr1, respectively (Figure 1A). Thus, because the miR-135 family 5p and 3p arms differ from each other, they target different mRNAs. The miR135a-5p multicopy, along with the nearly identical miR-135b-5p, modestly promote short-distance RGC axon regeneration up to approximately

Received 5 April 2026; accepted 28 July 2026;
https://doi.org/10.1016/j.omta.2026.201826.

[2]These authors contributed equally

Correspondence: Ephraim F. Trakhtenberg, Department of Neuroscience, University of Connecticut School of Medicine, 263 Farmington Ave, Farmington, CT 06030, USA.
E-mail: trakhtenberg@uchc.edu

Published by Elsevier Inc. on behalf of The American Society of Gene and Cell Therapy.
This is an open access article under the CC BY license (http://creativecommons.org/licenses/by/4.0/).

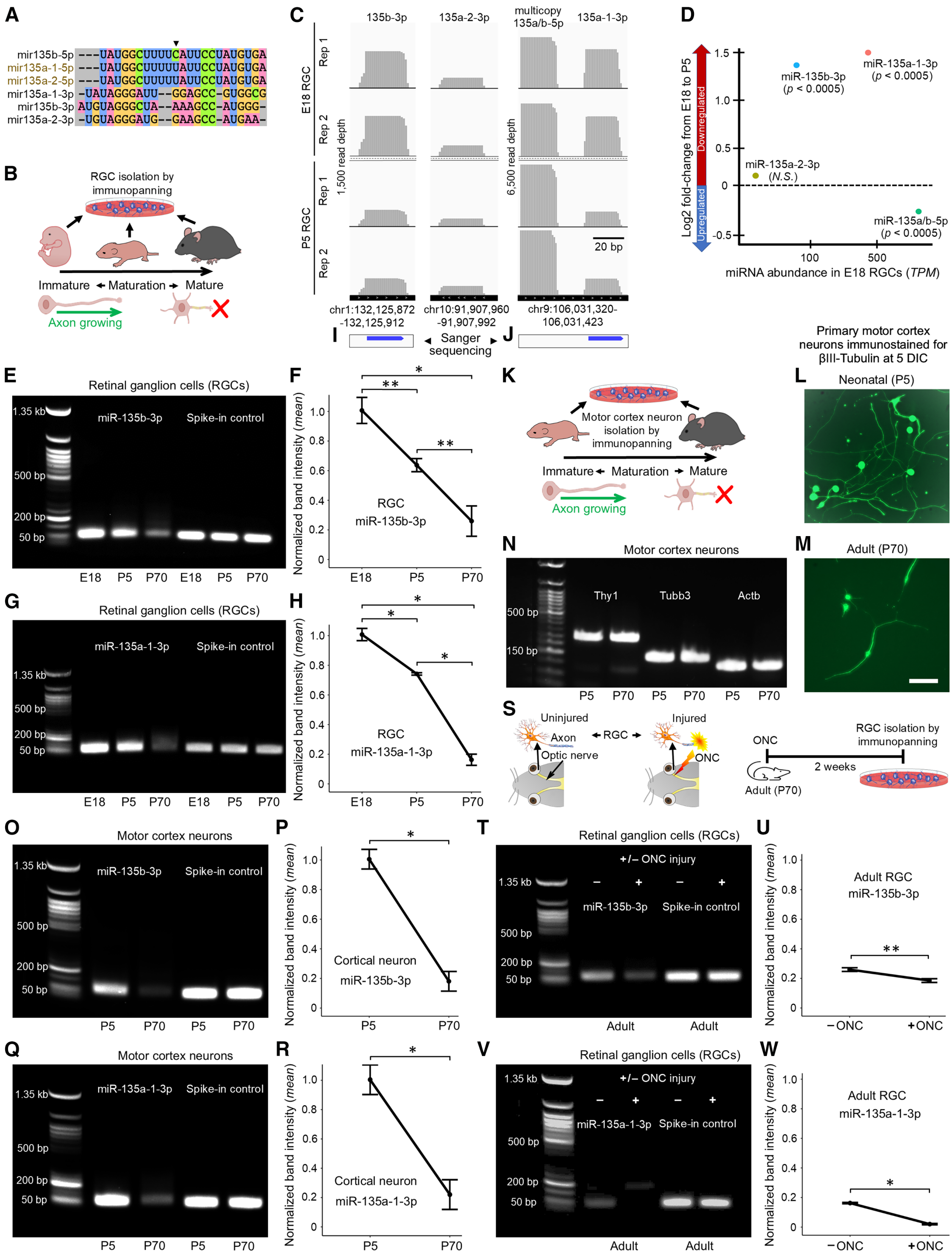

Figure 1. Characterization of miR-135 miRNA family members expression in CNS neurons during developmental maturation and after ONC injury
(A) Multiple sequencing alignment (MSA, Clustal Omega) of miR-135 miRNA family members shows substantial differences in the sequences of 3p arms and "multicopy" 5p arms. The "multicopy" miR-135a-1-5p and miR-135a-2-5p are annotated in brown font; miR-135b-5p is near-identical to the "multicopy," differing in only 1 bp (indicated by arrowhead). (B) Schematic of experimental design for small-RNA-seq and sqPCR analysis of the maturing RGCs isolated by Thy1-immunopanning after depletion of macrophages (see "methods"). (C) Visualization of normalized reads expression from RGC small-RNA-seq aligned to the miR135 loci in the mouse genome. From left to right:

(legend continued on next page)

~1 mm past the ONC site, potentially through targeting Klf4 in mature RGCs.[39] However, Klf4 is only transiently upregulated postnatally to irreversibly suppress RGCs' embryonic intrinsic axon growth capacity and is then downregulated in fully mature RGCs.[22] Thus, it is unclear how targeting Klf4 in already mature RGCs could be effective.[39] It is also unknown whether other miR-135 family members, which are distinct biologics that target different genes, are involved in neuronal development and/or axon regeneration. Here, we addressed these questions by: analyzing expression of miR-135 family members in RGC and cortical neuron development, testing whether the previously unstudied miR-135 family members regulate axon regeneration, probing rationale for targeting Klf4 in already mature RGCs, and exploring gene networks affected by axon-regenerating miR-135 family members identified herein.

RESULTS

Characterization of miR-135 family members' expression patterns in RGC and cortical neurons

Alignment of miR-135 family members shows that, while miRNAs share common features (highlighted in orange and green), the near-identical (i.e., only 1 bp mismatch) 5p arm sequences differ by almost 50% from the 3p arm sequences (Figure 1A). Thus, these 5p and 3p arms' miRNAs are distinct biologics that target different mRNAs (as detailed below). We used small-RNA-seq to profile miR-135 family members in RGCs purified (by Thy1-immunopanning after depletion of macrophages) from embryonic (E18) and postnatal (P5) developmental stages (see "methods"; Figure 1B). We selected E18 and P5 ages, because RGC's developmental axon growth capacity is still at its peak in E18 and then declines shortly after birth and is substantially reduced by P5.[17,18,45] Small-RNA-seq showed that, 5p arms' miR135a/b-5p (multicopy +1 bp mismatching isomiRs) are modestly upregulated, while 3p arm miR-135a-2-3p is not significantly regulated, during RGC maturation (Figures 1C and 1D). Thus, despite the treatment with miR135a/b-5p mimics modestly promoting RGC axon regeneration after ONC,[39] their endogenous expression patterns are not associated with positive regulation of developmental axon growth, which declines postnatally. However, the 3p arms, miR-135a-1-3p and miR-135b-3p, were downregulated substantially during RGC maturation (Figures 1C and 1D). We validated developmental regulation by a semi-quantitative PCR (sqPCR) method,[37] which confirmed miR-135a-1-3p and miR-135b-3p downregulation through postnatal stage of maturation and showed further downregulation by adult age (Figures 1E–1H). The specificity of the miRNAs' amplification in sqPCR was confirmed by genome-aligned Sanger sequencing, which matched the reads from small-RNA-seq (see "methods"; Figures 1I and 1J).

Then, we analyzed miR-135a-1-3p and miR-135b-3p expression in maturation of cortical neurons. Corticospinal motor neurons grow

miR-135b-3p mapping to chr1; miR-135a-2-3p mapping to chr10; and miR135a/b-5p and miR-135a-1-3p mapping to chr9. miR135a-5p "multicopy" of miR-135a-1-5p and miR-135a-2-5p, visualized here with net normalized reads aligned to the miR-135a-1-5p chr9 locus (as it cannot be determined from which of the two loci it originated) along with miR-135b-5p reads which differ by only 1 bp (see "methods"). Peaks aligned to miR-135b-3p and miR-135-a-1-3p encoding DNA regions are substantially higher in the E18 relative to P5 RGCs. Alignments from two replicates for each RGC age are shown; visualizations are using IGV Viewer. (D) DESeq2 differential expression abundance plot, with abundances of miR-135 family members in E18 RGCs (on *x* axis) versus FC from E18 to P5 RGCs on (*y* axis), visualizing miRNAs' abundance and developmental-regulation during RGC maturation. Statistical significance (indicated by $*p < 0.001$) of developmental regulation (E18 relative to P5 RGCs) determined using differential expression analysis by DESeq2, as marked; *N.S.*, not significant. Mean (SEM) shown; $n = 2$. Only miR-135b-3p and miR-135a-1-3p were downregulated significantly, while other miR-135 family members were either not developmentally regulated or modestly upregulated. TPM, transcripts per million, which is a common normalization method for expression in small-RNA-seq analysis (see "methods"). (E–H) Representative images of sqPCR analysis of miR-135b-3p and miR-135a-1-3p expression at E18, P5, and P70 (adult) stages of RGC development confirm that the sequences detected by small-RNA-seq are miR-135b-3p (E) and miR-135a-1-3p (G), which along with the adaptors match the expected ~85 bp gel bands. Quantitation of miR-135b-3p and miR-135a-1-3p gel bands intensity in samples prepared from E18, P5, and P70 RGCs (as marked) shows decline in their levels during maturation. Mean (SEM) shown; $n = 3$ samples for each condition (each sample was prepped from different animals). Data analyzed using ANOVA, overall $F = 20.7$, $p < 0.001$ for miR-135b-3p (F) and overall $F = 174.6$, $p < 0.001$ for miR-135a-1-3p (H), with *p* values of pairwise comparisons determined by post hoc LSD and significant differences indicated by $*p < 0.001$ and $**p < 0.05$. (I and J) Sanger sequencing of sqPCR products purified from the gel bands corresponding to miR-135b-3p (I) and miR-135a-1-3p (J) aligned to their respective loci (visualized in C above). Each Sanger sequencing result aligned to only one site in the genome, and blasting against the mouse miRbase database confirmed specificity of the sequences (miR-135b-3p score 105, *e* value 0.002; miR135a-1-3p score 105, *e* value 0.004). (K–N) Schematic of experimental design for sqPCR analysis of the maturing motor cortex neurons isolated by Thy1-immunopanning after depletion of macrophages (see "methods") (K). Primary neurons Thy1-immunopanned from P5 (L) and P70 adult (M) motor cortices cultured in defined medium (see "methods") and immunostained for neuronal-marker βIII-Tubulin to visualize neurite growth at 5 days in culture (DIC); scale bars, 100 μm. PCR for neuronal marker genes Tubb3 and Thy1 (which is enriched in projection neurons), along with the housekeeping Actb gene, confirmed enrichment of projection neurons isolated from single-cell suspension of P5 and P70 motor cortices by immunopanning (N). (O–R) Representative images of sqPCR analysis of miR-135b-3p and miR-135a-1-3p expression at P5 and P70 (adult) stages of motor cortex neuron development confirm that miR-135b-3p (O) and miR-135a-1-3p (Q) miRNAs match the expected ~85 bp (along with the adaptors) gel bands. Quantitation of miR-135b-3p and miR-135a-1-3p gel bands intensity in samples prepared from P5 and P70 motor cortex neurons (as marked) shows decline in their levels during maturation. Mean (SEM) shown; $n = 3$ samples for each condition (each sample was prepped from different animals). Data analyzed using *t* test two-tailed, with significant differences indicated by $*p < 0.001$ for miR-135b-3p (P) and miR-135a-1-3p (R). (S) Schematic of experimental design for sqPCR analysis of adult uninjured and ONC-injured RGCs. ONC injury performed in P70 (adult) mice, and animals sacrificed for isolation of RGCs by immunopanning (as described in B) at 2 weeks post-ONC. For control, RGCs were isolated the same way from age-matched uninjured mice (see "methods"). (T–W) Representative images of sqPCR analysis of miR-135b-3p and miR-135a-1-3p expression in uninjured and ONC-injured adult RGCs confirm that miR-135b-3p (T) and miR-135a-1-3p (V) miRNAs match the expected ~85 bp (along with the adaptors) gel bands. Quantitation of miR-135b-3p and miR-135a-1-3p gel bands intensity in samples prepared from uninjured and injured RGCs (as marked) shows further post-injury decline in their levels. Mean (SEM) shown; $n = 3$ samples for each condition (each sample was prepped from different animals). Data analyzed using *t* test two-tailed, with significant differences indicated by $*p < 0.001$ for miR-135b-3p (U) and $**p < 0.05$ for miR-135a-1-3p (W).

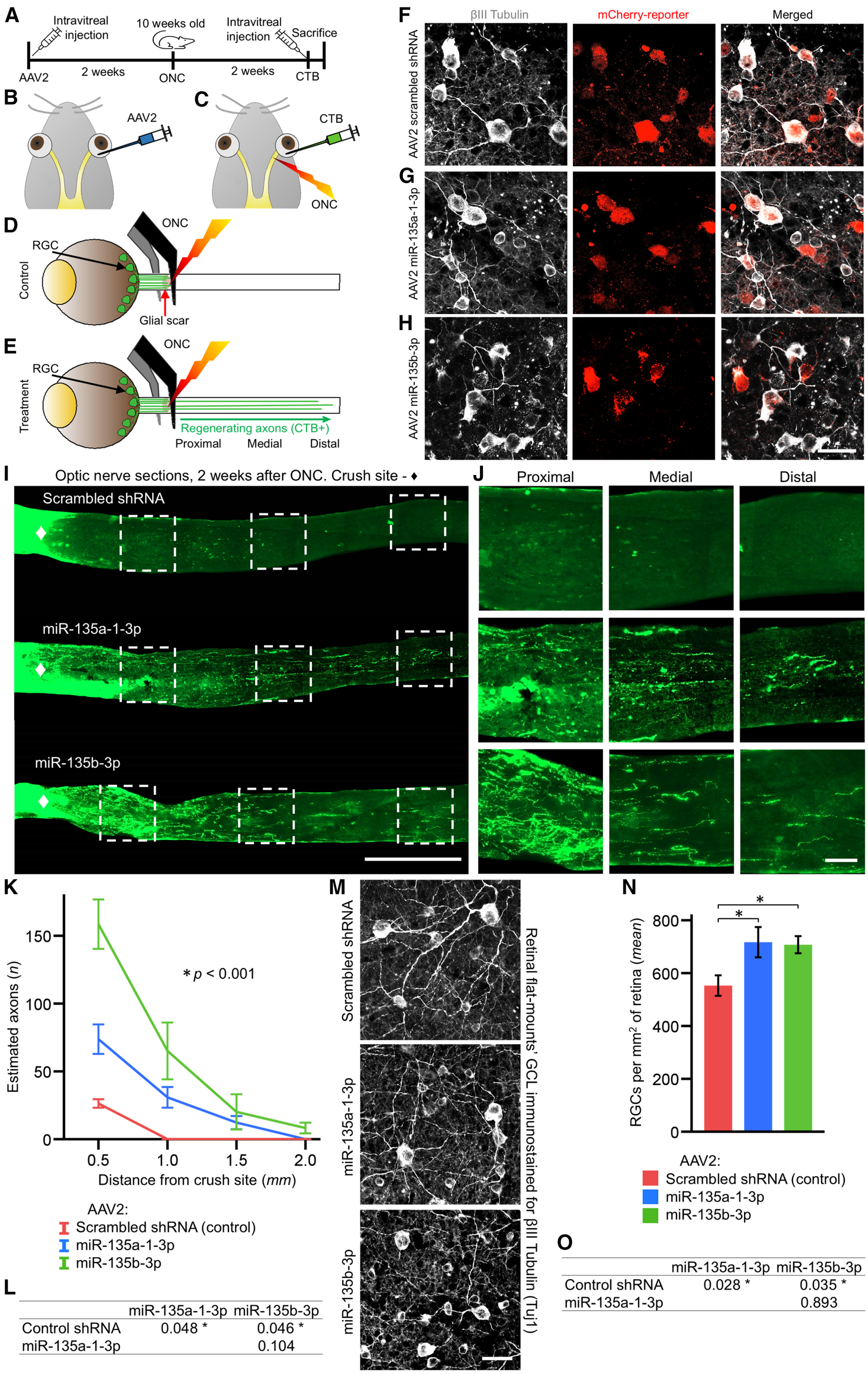

L

	miR-135a-1-3p	miR-135b-3p
Control shRNA	0.048 *	0.046 *
miR-135a-1-3p		0.104

O

	miR-135a-1-3p	miR-135b-3p
Control shRNA	0.028 *	0.035 *
miR-135a-1-3p		0.893

(legend on next page)

axons along the spinal cord through the first 2 weeks of postnatal development,[46,47] which differs from the RGC's developmental axon growth capacity, which peaks through E18 and declines shortly after birth.[18] Thus, cortical neurons were isolated by immunopanning for Thy1, which is expressed on cortical and RGC projection neurons,[48] from the primary motor cortex single-cell suspension of P5 and P70 mice (macrophages were depleted first, see "methods"; Figure 1K). We have previously validated the different neurite growth capacities of the isolated immature and adult RGCs in culture[21,49] (that were consistent with prior reports[18]), and confirmed here neurite growth capacity of the isolated P5 cortical neurons relative to the adult cortical neurons which barely grow neurites (Figures 1L and1M). Isolation of P5 and P70 cortical neurons was validated using PCR for neuronal marker-genes Tubb3 (encodes βIII-Tubulin) and Thy1, and control housekeeping gene Actb (encodes β-actin), visualized by gel electrophoresis in samples from both ages (Figure 1N). Analysis by sqPCR showed that miR-135a-1-3p and miR-135b-3p were developmentally downregulated in the motor cortex neurons (Figures 1O–1R). We also tested whether miR-135a-1-3p and miR-135b-3p respond to axonal ONC injury (Figure 1S) and found that both are further reduced in the RGCs by 2 weeks post-ONC (Figures 1T–1W). These data suggest that developmental-downregulation and, potentially, roles of miR-135a-1-3p and miR-135b-3p may be conserved across CNS projection neurons.

miR-135a-1-3p and miR-135b-3p mimics promote neuroprotection and axon regeneration after injury *in vivo*

To test whether reinstating miR-135a-1-3p and miR-135b-3p expression in injured adult RGCs toward embryonic (i.e., higher) levels affects neuroprotection and/or axon regeneration, we used AAV2 vectors expressing miR-135a-1-3p-mimic, miR-135b-3p-mimic, or scrambled-control shRNA, and co-expressing an mCherry reporter, for intravitreal targeting in RGCs. To confirm persistence of AAV2 expression in RGCs after injury, the viruses were injected intravitreally in adult mice, and 2 weeks later ONC was performed (experimental design shown in Figures 2A–2E). At 2 weeks post-ONC, the retinas were immunostained for βIII-Tubulin neuronal marker, which labels the RGCs within the retinal ganglion cell layer (GCL). Co-localization of an mCherry-reporter and βIII-Tubulin signals by confocal microscopy confirmed vector expression in RGCs, with transduction efficiency of ∼30% within a range of prior reports that also used intravitreal AAV2 for targeting RGCs[12,21,25,50–52] (Figures 2F–2H). Then, we tested whether miR-135a-1-3p and/or miR-135b-3p mimics would promote RGC survival and axon regeneration after injury. We used an established assay in which the optic nerve is injured by crush to sever all RGC axons, and then RGC survival and axon regeneration are quantified at 2 weeks post-injury, using βIII-Tubulin immunostaining and anterograde axon labeling with cholera toxin subunit B (CTB) injected 1 day before sacrifice, respectively (Figure 2A).[12,25,50–53] We found that by 2 weeks post-ONC, relative to only minor axonal sprouting past the injury site detected in control animals, miR-135a-1-3p and miR-135b-3p mimics promoted axon regeneration reaching approximately ∼2 mm past the injury site (Figures 2I–2L). Both treatments also promoted RGC survival compared to the control group (Figures 2M–2O).

Next, we asked whether the positive effects of these miRNA gene therapies are sustained over a longer term, and whether their effectiveness could be enhanced by co-treating with a small recombinant protein containing fibronectin's RGD-motif (RP-RGD), which we previously developed as an axon growth-supporting factor that also augments the efficacy of other treatments.[49] AAV2 vectors were administered as above at 2 weeks pre-injury, and RP-RGD or vehicle were injected intravitreally immediately after ONC. RGC survival and axon regeneration were analyzed at 6 weeks post-ONC, which is a later time point we used for evaluating other axon regeneration treatments in prior studies[49,51] (Figure 3A). We found no spontaneous axon repair or regeneration in the control condition. Moreover, diminished CTB signal in most of the pre-ONC area indicates retrograde axon degeneration from the injury site toward

Figure 2. AAV2 miR-135a-1-3p and miR-135b-3p mimics promote RGC survival and axon regeneration after optic nerve injury

(A–E) Experimental timeline (A) with schematic representations: AAV2 viral vectors injected intravitreally in 8-week-old mice (B), and 2 weeks later ONC performed (C). Animals sacrificed for histological analysis 2 weeks post-ONC. For axon regeneration studies (shown later in I–K), an axonal tracer CTB (conjugated to Alexa Fluor 488 dye) is injected intravitreally 1 day before sacrifice (C). Because axons are severed by the injury, CTB is not transported beyond the ONC site (D), unless axons regenerated in response to a treatment (E). (F–H) Confocal microscopy with 63× objective was used for determining co-localization of vector reporters and RGC marker in the same cells. High-magnification representative images of the immunostained retinal flatmounts show that: an mCherry reporter of transduction/expression localized to the βIII-Tubulin+ cells (βIII-Tubulin is a neuronal marker which labels the RGCs within the retinal GCL), and that transduced cells are detected at comparable proportions across conditions: scrambled shRNA-control (F), miR-135a-1-3p-mimic (G), and miR-135b-3p-mimic (H), as marked. Scale bars, 20 μm. (I and J) Representative images of the optic nerve longitudinal sections with CTB-labeled axons at 2 weeks post-ONC from the animals pre-treated with AAV2 expressing miR-135a-1-3p-mimic, miR-135b-3p-mimic, or scrambled-control shRNA, as marked (I). Insets: representative images of the optic nerve regions proximal and distal to the injury site are magnified for better visualization of the axons or their absence (J). The edges of the tissue were optically trimmed (i.e., cropped-out) due to artifactual autofluorescence that is common at tissue edges (see "methods"). Scale bars, 500 μm (main images), 50 μm (insets). (K and L) Quantitation of CTB-labeled regenerating axons at 2 weeks post-ONC, at increasing distances from the injury site, after pre-treatment with AAV2 expressing miR-135a-1-3p-mimic, miR-135b-3p-mimic, or scrambled-control shRNA, as marked (K). Data analyzed using repeated measures ANOVA with post hoc LSD, sphericity assumed, overall $F = 26.4$, $p < 0.001$; with p values of pairwise comparisons determined by post hoc LSD shown in the table, and significant differences ($p < 0.05$) indicated by an asterisk * (L). Mean (SEM) shown; $n = 4$ cases per group. (M–O) Representative images of the retinal flatmounts immunostained for βIII-Tubulin at 2 weeks post-ONC, pre-treated with experimental and control AAV2 vectors, as marked. Scale bars, 20 μm (M). Quantitation of RGC (βIII-Tubulin+ cells) survival in retinal flatmounts' GCL at 2 weeks post-ONC, from the treatment conditions as marked (N). Data analyzed using ANOVA, overall $F = 4.94$, $p < 0.05$, with p values of pairwise comparisons determined by post hoc LSD shown in the table, and significant differences ($p < 0.05$) indicated by an asterisk * (O). Mean (SEM) shown, $n = 4$ cases per group.

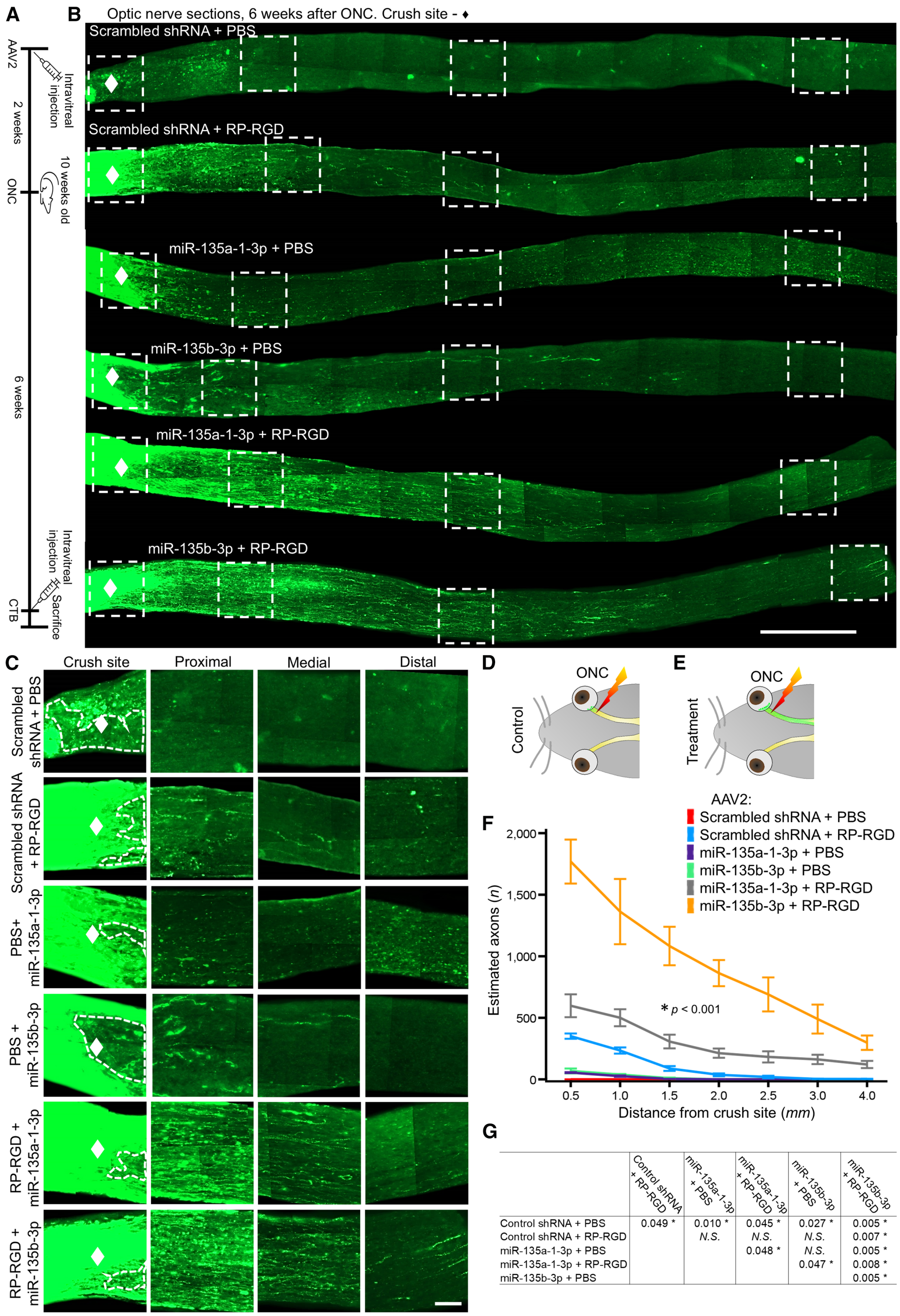

	Control shRNA + RP-RGD	miR-135a-1-3p + PBS	miR-135a-1-3p + RP-RGD	miR-135b-3p + PBS	miR-135b-3p + RP-RGD
Control shRNA + PBS	0.049 *	0.010 *	0.045 *	0.027 *	0.005 *
Control shRNA + RP-RGD		*N.S.*	*N.S.*	*N.S.*	0.007 *
miR-135a-1-3p + PBS			0.048 *	*N.S.*	0.005 *
miR-135a-1-3p + RP-RGD				0.047 *	0.008 *
miR-135b-3p + PBS					0.005 *

(legend on next page)

the retina, which became apparent by 6 weeks (compared to 2 weeks in Figures 2I and 2J) in the control condition[6,54] (Figures 3B and 3C). As compared to control, significant axon regeneration was sustained (relative to regeneration at 2 weeks) in all the treatment conditions, without substantial retrograde degeneration in the pre-ONC area which occurred in control, at 6 weeks post-ONC (Figures 3B–3E). Remarkably, compared to individual treatments, in the experimental co-treatment conditions substantially more long axons regenerated (Figures 3F and 3G), with some axons regenerating through the entire optic nerve and optic chiasm and in half of the cases entering the optic tracts (Figures S1A–S1D). Furthermore, RGC survival was significantly higher in the individual treatments compared to the control condition, and to an even greater extent in the co-treatments, at 6 weeks post-ONC (Figures S1A–S1C). As most untreated RGCs die progressively after ONC injury,[6,54] sustainment of RGC survival over a long term is important. Thus, co-treatment with RP-RGD and miR-135a-1-3p or miR-135b-3p promotes survival and long-distance axon regeneration of RGCs after ONC injury.

miR-135a/b-5p and miR-135a/b-1-3p predicted to target different genes

To explore whether the 3p arms' miR-135a-1-3p or miR-135b-3p targets overlap those of 5p arms' miR-135a/b-5p, we used the bioinformatic tool miRanda, which predicts miRNAs' gene targets[55,56] (Figure 1A). For genes expressed in injured adult RGCs based on scRNA-seq, we identified mRNA transcript isoforms by bulk-mRNA-seq profiling of injured RGCs (see "methods"). We then analyzed for potential miRNA substrates within the 3′ UTRs of these transcripts, because 3′ UTRs are established to be preferentially targeted by miRNAs in general[41] (the 3′ UTR sequences are in Table S1; see "methods"). The 5p and 3p arms' miRNAs were predicted to target 3′ UTRs of different subsets of mRNA transcripts expressed in injured adult RGCs (shown rank-ordered by miRanda in Figures S2A–S2G). Because Klf4 peaks only transiently in postnatal RGCs to switch-off embryonic intrinsic axon growth capacity and then downregulates by adult age,[22] targeting Klf4 in adult RGCs may promote axon growth[39] only if Klf4 is re-upregulated after injury. Although scRNA-seq[57] detected only modest Klf4 levels in the ONC-injured RGC subtypes (annotated as described previously[14,57]; Figure S2H), this is consistent with the prior report,[39] as Klf4 was among the top predicted 5p arm miRNAs' targets (Figures S2A, S2B, and S2E). However, Klf4 is not a predicted target of 3p arms' miR-135b-3p or miR-135a-1-3p (Figures S2C–S2D, S2F and S2G). Thus, 5p and 3p arms miR-135 miRNAs are predicted to target different genes in the RGCs.

miR-135a-1-3p and miR-135b-3p mimics affect gene networks in injured RGCs

miRNAs could co-target multiple mRNAs to regulate cellular processes through indirectly affected downstream gene networks.[40,41] Thus, to build hypotheses regarding the underlying mechanisms, we utilized the gene annotation/ontology (GO) database for exploring the gene networks affected by the treatments.[58,59] We used a pipeline we previously described[37] to explore the differentially expressed genes (DEGs) by bulk-mRNA-seq of miR-135a-1-3p- and miR-135b-3p-mimic-treated RGCs, relative to scrambled-control shRNA-treated RGCs (experimental timeline in Figure S2I; the DEG list is in Table S2; see "methods"). Although the enriched GO terms (see "methods") were generic, several genes previously associated with neurodevelopment and/or neuroprotection were in the gene networks: Dpysl2,[24,60,61] Sox11,[62–64] and Gal[65,66] were upregulated in both treatments, whereas Kif5b[67] was upregulated in the miR-135a-1-3p treatment while Anxa2[68] and Myc[69] were upregulated and Pten[53] downregulated in miR-135b-3p treatment (Figures S2J and S2K). Thus, the affected gene networks embed downstream regulators of axon growth.

DISCUSSION

The roles of miRNAs in neuronal development and repair after CNS injury continue to emerge.[26–39] Studying miRNAs in an arm-specific and neuronal type-specific manner *in vivo* is necessary for advancing the neuronal miRNAs research field. Here, we show that the miR-135 family 3p arms, miR-135a-1-3p and miR-135b-3p, are (1) developmentally downregulated in RGC and motor cortex neurons during maturation-associated loss of intrinsic axon growth capacity,[13,14,17,18] (2) further downregulated in the RGCs after injury, and (3) targeting injured RGCs via AAV2 with their mimics promotes RGC survival and axon regeneration. Remarkably, axon regeneration promoted by miR-135a-1-3p and miR-135b-3p co-treated with a fibronectin-based small recombinant protein we recently developed[49] enabled a subset of axons to regenerate through the full-length of the optic nerve, with some axons in half of the cases even traversing the optic chiasm and entering the optic tract by the 6-week post-injury endpoint we tested. Future studies would need to

Figure 3. Co-treating with RP-RGD and AAV2 miR-135a-1-3p or miR-135b-3p mimics augments axon regeneration sustained over longer period
(A) Experimental timeline: 8-week-old mice pre-treated with AAV2 expressing miR-135a-1-3p-mimic, miR-135b-3p-mimic, or scrambled-control shRNA, and co-treated with RP-RGD or vehicle. ONC injury performed 2 weeks later. Animals sacrificed for histological analysis 6 weeks post-ONC. Axonal tracer CTB injected intravitreally 1 day before sacrifice. (B–E) Representative images of the optic nerve longitudinal cryosections with CTB-labeled axons at 6 weeks post-ONC from the animals treated as marked (B). Insets: representative images of the optic nerve regions (outlined by dashed boxes in B) proximal and distal to the injury site magnified for better visualization of the axons or their absence, and of the ONC site with a larger retrogradely degenerating pre-crush area (indicated by the diminished CTB signal outlined by dashed line) in the control compared to treatment conditions (C). Schematic of the injured central visual pathway segment containing the optic nerve in the control condition (D), and a representation of axon regeneration through the full-length of the optic nerve achieved by the co-treatments, compared to control, at 6 weeks post-ONC (E). The edges of the tissue were optically trimmed (i.e., cropped-out) due to artifactual autofluorescence that is common at tissue edges (see "methods"). Scale bars, 500 μm (main images), 50 μm (insets). (F and G) Quantitation of CTB-labeled regenerating axons at 6 weeks after ONC, at increasing distances from the injury site along the optic nerve, from the animals treated as marked (F). Data analyzed using repeated measures ANOVA, sphericity assumed, overall $F = 108.5$, $p < 0.001$, with p values of pairwise comparisons determined by posthoc LSD shown in the table, and significant differences ($p < 0.05$) indicated by an asterisk * (G). Mean (SEM) shown; $n = 4$ cases per group.

explore the full potential of these treatments by leveraging emerging improved AAV2-based vectors with higher transduction efficiency and using longer-term endpoints which, per prior reports, need to be 10–12-week post-ONC in order to allow for the regenerating axons to reach post-synaptic targets in the brain.[70,71] Following determination of anatomical reinnervation of post-synaptic targets in the brain, restoration of at least simple visual functions could be tested through electrophysiological and behavioral assays.

miRNAs are a promising class of biologics for therapeutic applications, because of their small size and the ability to concurrently target multiple mRNAs, in the same cell,[40–42] that may need to be co-regulated for coordinating complex biological processes such as axonal tissue regeneration. This is consistent with miR-135a-1-3p and miR-135b-3p mimics targeted to injured RGCs affecting GO-linked gene networks that embedded several downstream genes previously associated with neurodevelopment and/or neuroprotection. For example, Dpysl2,[24,60,61] Sox11,[62–64] Gal,[65,66] Kif5b,[67] Anxa2,[68] and Myc[69] were indirectly upregulated downstream of targets such as Pten,[53] which were knocked-down by the miRNA mimics. It would be important for future studies to discern between the direct and indirect downstream mRNA targets, by screening the predicted DEGs for direct miRNA-mRNA interaction using reporter assays. However, it may not be possible to recapitulate the full phenotype by targeting a single mRNA, and most alternative approaches also co-target a combination of factors to promote long-distance axon regeneration. Therefore, future directions toward a deeper understanding of the miR-135a-1-3p and miR-135b-3p downstream mechanisms may need to study crosstalk between the cellular pathways regulated by a miRNA in order to determine their relative contributions to neuroprotection and axon regeneration. Future research will also need to determine which RGC subtypes respond better to the treatments, and whether these miRNA mimics are also neurotherapeutic in traumatic spinal cord and brain injury models, particularly if the miR-135 3p and 5p arms' mimics are co-administered to achieve potentially stronger efficacy.

MATERIALS AND METHODS

Materials and methods are provided in the supplemental material along with the supplemental data legends.

DATA AND CODE AVAILABILITY

- The plasmid for miR-135a-1-3p-mimic and miR-135b-3p-mimic AAV2 vectors will be shared for academic purposes. This study did not generate any other new unique reagents.
- RNA sequencing data generated for this study have been deposited in publicly accessible NCBI repository under Gene Expression Omnibus (GEO) accession numbers GSE337069 and under NCBI Sequence Read Archive (SRA) BioProject accession number PRJNA1483696. GEO accession numbers for the previously generated RNA sequencing data used in this study are provided in the supplemental information.
- This paper does not report original code.
- The supplemental data legends are provided in the supplemental information. Any additional information required to reanalyze the data reported in this paper is available from the lead contact upon request.

ACKNOWLEDGMENTS

This work was supported by grants from the National Institutes of Health (NIH) NEI-R01-EY029739 and NEI-R01-EY038322, to E.F.T. We are grateful to the High Performance Computing Facility, University of Connecticut for bioinformatics support. We thank Ashiti Damania, William Theune, and Jian Xing (students, University of Connecticut School of Medicine) for technical assistance.

AUTHOR CONTRIBUTIONS

J.H.B., A.L., A.J.B., and C.D. performed the experiments; E.F.T. designed the study and wrote the manuscript.

DECLARATION OF INTERESTS

The authors declare no competing interests.

SUPPLEMENTAL INFORMATION

Supplemental information can be found online at https://doi.org/10.1016/j.omta.2026.201826.

REFERENCES

1. Hug, A., and Weidner, N. (2012). From bench to beside to cure spinal cord injury: lost in translation? Int. Rev. Neurobiol. *106*, 173–196. https://doi.org/10.1016/B978-0-12-407178-0.00008-9.
2. Benowitz, L.I., and Carmichael, S.T. (2010). Promoting axonal rewiring to improve outcome after stroke. Neurobiol. Dis. *37*, 259–266. https://doi.org/10.1016/j.nbd.2009.11.009.
3. Sozmen, E.G., Hinman, J.D., and Carmichael, S.T. (2012). Models that matter: white matter stroke models. Neurotherapeutics *9*, 349–358. https://doi.org/10.1007/s13311-012-0106-0.
4. Armstrong, R.C., Mierzwa, A.J., Marion, C.M., and Sullivan, G.M. (2016). White matter involvement after TBI: Clues to axon and myelin repair capacity. Exp. Neurol. *275*, 328–333. https://doi.org/10.1016/j.expneurol.2015.02.011.
5. Hill, C.S., Coleman, M.P., and Menon, D.K. (2016). Traumatic Axonal Injury: Mechanisms and Translational Opportunities. Trends Neurosci. *39*, 311–324. https://doi.org/10.1016/j.tins.2016.03.002.
6. Ghaffarieh, A., and Levin, L.A. (2012). Optic nerve disease and axon pathophysiology. Int. Rev. Neurobiol. *105*, 1–17. https://doi.org/10.1016/B978-0-12-398309-1.00002-0.
7. You, Y., Gupta, V.K., Li, J.C., Klistorner, A., and Graham, S.L. (2013). Optic neuropathies: characteristic features and mechanisms of retinal ganglion cell loss. Rev. Neurosci. *24*, 301–321. https://doi.org/10.1515/revneuro-2013-0003.
8. Benowitz, L.I., He, Z., and Goldberg, J.L. (2017). Reaching the brain: Advances in optic nerve regeneration. Exp. Neurol. *287*, 365–373. https://doi.org/10.1016/j.expneurol.2015.12.015.
9. Wang, Y., Brown, D.P., Duan, Y., Kong, W., Watson, B.D., and Goldberg, J.L. (2013). A novel rodent model of posterior ischemic optic neuropathy. JAMA Ophthalmol. *131*, 194–204. https://doi.org/10.1001/2013.jamaophthalmol.271.
10. Levkovitch-Verbin, H. (2004). Animal models of optic nerve diseases. Eye (Lond) *18*, 1066–1074. https://doi.org/10.1038/sj.eye.6701576.
11. Yin, Y., and Benowitz, L.I. (2018). In Vitro and In Vivo Methods for Studying Retinal Ganglion Cell Survival and Optic Nerve Regeneration. Methods Mol. Biol. *1695*, 187–205. https://doi.org/10.1007/978-1-4939-7407-8_16.
12. Kim, J., Sajid, M.S., and Trakhtenberg, E.F. (2018). The extent of extra-axonal tissue damage determines the levels of CSPG upregulation and the success of experimental axon regeneration in the CNS. Sci. Rep. *8*, 9839. https://doi.org/10.1038/s41598-018-28209-z.
13. Poplawski, G.H.D., Kawaguchi, R., Van Niekerk, E., Lu, P., Mehta, N., Canete, P., Lie, R., Dragatsis, I., Meves, J.M., Zheng, B., et al. (2020). Injured adult neurons regress to an embryonic transcriptional growth state. Nature *581*, 77–82. https://doi.org/10.1038/s41586-020-2200-5.
14. Rheaume, B.A., Xing, J., Lukomska, A., Theune, W.C., Damania, A., Sjogren, G., and Trakhtenberg, E.F. (2023). Pten inhibition dedifferentiates long-distance axon-regenerating intrinsically photosensitive retinal ganglion cells and upregulates

mitochondria-associated Dynlt1a and Lars2. Development *150*, dev201644. https://doi.org/10.1242/dev.201644.

15. Trakhtenberg, E.F. (2025). Single-cell transcriptomics-enabled advances in experimental optic nerve axon regeneration research. In Proteomics, Multi-Omics and Systems Biology in Optic Nerve Regeneration, S.K. Bhattacharya, ed. (Academic Press), pp. 207–223. https://doi.org/10.1016/B978-0-443-15580-2.00014-X.
16. Sellés-Navarro, I., Ellezam, B., Fajardo, R., Latour, M., and McKerracher, L. (2001). Retinal ganglion cell and nonneuronal cell responses to a microcrush lesion of adult rat optic nerve. Exp. Neurol. *167*, 282–289. https://doi.org/10.1006/exnr.2000.7573.
17. Chen, D.F., Jhaveri, S., and Schneider, G.E. (1995). Intrinsic changes in developing retinal neurons result in regenerative failure of their axons. Proc. Natl. Acad. Sci. USA *92*, 7287–7291. https://doi.org/10.1073/pnas.92.16.7287.
18. Goldberg, J.L., Klassen, M.P., Hua, Y., and Barres, B.A. (2002). Amacrine-signaled loss of intrinsic axon growth ability by retinal ganglion cells. Science *296*, 1860–1864. https://doi.org/10.1126/science.1068428.
19. Apara, A., Galvao, J., Wang, Y., Blackmore, M., Trillo, A., Iwao, K., Brown, D.P., Fernandes, K.A., Huang, A., Nguyen, T., et al. (2017). KLF9 and JNK3 Interact to Suppress Axon Regeneration in the Adult CNS. J. Neurosci. *37*, 9632–9644. https://doi.org/10.1523/JNEUROSCI.0643-16.2017.
20. Blackmore, M.G., Wang, Z., Lerch, J.K., Motti, D., Zhang, Y.P., Shields, C.B., Lee, J.K., Goldberg, J.L., Lemmon, V.P., and Bixby, J.L. (2012). Krüppel-like Factor 7 engineered for transcriptional activation promotes axon regeneration in the adult corticospinal tract. Proc. Natl. Acad. Sci. USA *109*, 7517–7522. https://doi.org/10.1073/pnas.1120684109.
21. Trakhtenberg, E.F., Wang, Y., Morkin, M.I., Fernandez, S.G., Mlacker, G.M., Shechter, J.M., Liu, X., Patel, K.H., Lapins, A., Yang, S., et al. (2014). Regulating Set-β's Subcellular Localization Toggles Its Function between Inhibiting and Promoting Axon Growth and Regeneration. J. Neurosci. *34*, 7361–7374. https://doi.org/10.1523/JNEUROSCI.3658-13.2014.
22. Moore, D.L., Blackmore, M.G., Hu, Y., Kaestner, K.H., Bixby, J.L., Lemmon, V.P., and Goldberg, J.L. (2009). KLF family members regulate intrinsic axon regeneration ability. Science *326*, 298–301. https://doi.org/10.1126/science.1175737.
23. Liu, K., Lu, Y., Lee, J.K., Samara, R., Willenberg, R., Sears-Kraxberger, I., Tedeschi, A., Park, K.K., Jin, D., Cai, B., et al. (2010). PTEN deletion enhances the regenerative ability of adult corticospinal neurons. Nat. Neurosci. *13*, 1075–1081. https://doi.org/10.1038/nn.2603.
24. Lukomska, A., Theune, W.C., Xing, J., Frost, M.P., Damania, A., Gupta, M., and Trakhtenberg, E.F. (2023). Experimental gene expression of developmentally downregulated Crmp1, Crmp4, and Crmp5 promotes axon regeneration and retinal ganglion cell survival after optic nerve injury. Brain Res. *1809*, 148368. https://doi.org/10.1016/j.brainres.2023.148368.
25. Lukomska, A., Kim, J., Rheaume, B.A., Xing, J., Hoyt, A., Lecky, E., Steidl, T., and Trakhtenberg, E.F. (2021). Developmentally upregulated Transcriptional Elongation Factor A like 3 suppresses axon regeneration after optic nerve injury. Neurosci. Lett. *765*, 136260. https://doi.org/10.1016/j.neulet.2021.136260.
26. Liu, C.M., Wang, R.Y., Zhou, F.Q., Saijilafu, Jiao, Z.X., Jiao, Z.X., and Zhang, B.Y. (2013). MicroRNA-138 and SIRT1 form a mutual negative feedback loop to regulate mammalian axon regeneration. Genes Dev. *27*, 1473–1483. https://doi.org/10.1101/gad.209619.112.
27. Wu, D., and Murashov, A.K. (2013). MicroRNA-431 regulates axon regeneration in mature sensory neurons by targeting the Wnt antagonist Kremen1. Front. Mol. Neurosci. *6*, 35. https://doi.org/10.3389/fnmol.2013.00035.
28. Li, S., Wang, X., Gu, Y., Chen, C., Wang, Y., Liu, J., Hu, W., Yu, B., Wang, Y., Ding, F., et al. (2015). Let-7 microRNAs regenerate peripheral nerve regeneration by targeting nerve growth factor. Mol. Ther. *23*, 423–433. https://doi.org/10.1038/mt.2014.220.
29. Mead, B., Cullather, E., Nakaya, N., Niu, Y., Kole, C., Ahmed, Z., and Tomarev, S. (2020). Viral delivery of multiple miRNAs promotes retinal ganglion cell survival and functional preservation after optic nerve crush injury. Exp. Eye Res. *197*, 108071. https://doi.org/10.1016/j.exer.2020.108071.
30. Mead, B., and Tomarev, S. (2022). The role of miRNA in retinal ganglion cell health and disease. Neural Regen. Res. *17*, 516–522. https://doi.org/10.4103/1673-5374.320974.
31. Gaudet, A.D., Mandrekar-Colucci, S., Hall, J.C.E., Sweet, D.R., Schmitt, P.J., Xu, X., Guan, Z., Mo, X., Guerau-de-Arellano, M., and Popovich, P.G. (2016). miR-155 Deletion in Mice Overcomes Neuron-Intrinsic and Neuron-Extrinsic Barriers to Spinal Cord Repair. J. Neurosci. *36*, 8516–8532. https://doi.org/10.1523/JNEUROSCI.0735-16.2016.
32. Zhang, Y., Ueno, Y., Liu, X.S., Buller, B., Wang, X., Chopp, M., and Zhang, Z.G. (2013). The MicroRNA-17-92 cluster enhances axonal outgrowth in embryonic cortical neurons. J. Neurosci. *33*, 6885–6894. https://doi.org/10.1523/JNEUROSCI.5180-12.2013.
33. Baudet, M.L., Zivraj, K.H., Abreu-Goodger, C., Muldal, A., Armisen, J., Blenkiron, C., Goldstein, L.D., Miska, E.A., and Holt, C.E. (2011). miR-124 acts through CoREST to control onset of Sema3A sensitivity in navigating retinal growth cones. Nat. Neurosci. *15*, 29–38. https://doi.org/10.1038/nn.2979.
34. Hancock, M.L., Preitner, N., Quan, J., and Flanagan, J.G. (2014). MicroRNA-132 is enriched in developing axons, locally regulates Rasa1 mRNA, and promotes axon extension. J. Neurosci. *34*, 66–78. https://doi.org/10.1523/JNEUROSCI.3371-13.2014.
35. Dajas-Bailador, F., Bonev, B., Garcez, P., Stanley, P., Guillemot, F., and Papalopulu, N. (2012). microRNA-9 regulates axon extension and branching by targeting Map1b in mouse cortical neurons. Nat. Neurosci. *15*, 697–699. https://doi.org/10.1038/nn.3082.
36. Hintermayer, M.A., Juźwik, C.A., Morquette, B., Hua, E., Zhang, J., Drake, S., Shi, S.S., Rambaldi, I., Vangoor, V., Pasterkamp, J., et al. (2024). A miR-383-5p Signaling Hub Coordinates the Axon Regeneration Response to Inflammation. J. Neurosci. *44*, e1822232024. https://doi.org/10.1523/JNEUROSCI.1822-23.2024.
37. Lukomska, A., Theune, W.C., Frost, M.P., Xing, J., Kearney, A., and Trakhtenberg, E.F. (2024). Upregulation of developmentally-downregulated miR-1247-5p promotes neuroprotection and axon regeneration in vivo. Neurosci. Lett. *823*, 137662. https://doi.org/10.1016/j.neulet.2024.137662.
38. Hintermayer, M.A., Hua, E.M.L., Noor, M., Rambaldi, I., and Fournier, A.E. (2026). A synthetic circular RNA targeting miR-340-5p promotes optic nerve regeneration and retinal ganglion cell survival following axotomy. Exp. Neurol. *396*, 115527. https://doi.org/10.1016/j.expneurol.2025.115527.
39. van Battum, E.Y., Verhagen, M.G., Vangoor, V.R., Fujita, Y., Derijck, A.A.H.A., O'Duibhir, E., Giuliani, G., de Gunst, T., Adolfs, Y., Lelieveld, D., et al. (2018). An Image-Based miRNA Screen Identifies miRNA-135s As Regulators of CNS Axon Growth and Regeneration by Targeting Krüppel-like Factor 4. J. Neurosci. *38*, 613–630. https://doi.org/10.1523/JNEUROSCI.0662-17.2017.
40. Brown, M., Suryawanshi, H., Hafner, M., Farazi, T.A., and Tuschl, T. (2013). Mammalian miRNA curation through next-generation sequencing. Front. Genet. *4*, 145. https://doi.org/10.3389/fgene.2013.00145.
41. Gebert, L.F.R., and MacRae, I.J. (2019). Regulation of microRNA function in animals. Nat. Rev. Mol. Cell Biol. *20*, 21–37. https://doi.org/10.1038/s41580-018-0045-7.
42. Tomasello, L., Distefano, R., Nigita, G., and Croce, C.M. (2021). The MicroRNA Family Gets Wider: The IsomiRs Classification and Role. Front. Cell Dev. Biol. *9*, 668648. https://doi.org/10.3389/fcell.2021.668648.
43. Jiang, D., Yin, C., Yu, A., Zhou, X., Liang, W., Yuan, Z., Xu, Y., Yu, Q., Wen, T., and Zhang, D. (2006). Duplication and expression analysis of multicopy miRNA gene family members in Arabidopsis and rice. Cell Res. *16*, 507–518. https://doi.org/10.1038/sj.cr.7310062.
44. Guo, L., and Lu, Z. (2010). Global expression analysis of miRNA gene cluster and family based on isomiRs from deep sequencing data. Comput. Biol. Chem. *34*, 165–171. https://doi.org/10.1016/j.compbiolchem.2010.06.001.
45. Benowitz, L.I., and Yin, Y. (2010). Optic nerve regeneration. Arch. Ophthalmol. *128*, 1059–1064. https://doi.org/10.1001/archophthalmol.2010.152.
46. Sahni, V., Shnider, S.J., Jabaudon, D., Song, J.H.T., Itoh, Y., Greig, L.C., and Macklis, J.D. (2021). Corticospinal neuron subpopulation-specific developmental genes prospectively indicate mature segmentally specific axon projection targeting. Cell Rep. *37*, 109843. https://doi.org/10.1016/j.celrep.2021.109843.
47. Canty, A.J., and Murphy, M. (2008). Molecular mechanisms of axon guidance in the developing corticospinal tract. Prog. Neurobiol. *85*, 214–235. https://doi.org/10.1016/j.pneurobio.2008.02.001.

48. Porrero, C., Rubio-Garrido, P., Avendaño, C., and Clascá, F. (2010). Mapping of fluorescent protein-expressing neurons and axon pathways in adult and developing Thy1-eYFP-H transgenic mice. Brain Res. *1345*, 59–72. https://doi.org/10.1016/j.brainres.2010.05.061.

49. Lukomska, A., Rheaume, B.A., Frost, M.P., Theune, W.C., Xing, J., Damania, A., and Trakhtenberg, E.F. (2024). Augmenting fibronectin levels in injured adult CNS promotes axon regeneration in vivo. Exp. Neurol. *379*, 114877. https://doi.org/10.1016/j.expneurol.2024.114877.

50. Yungher, B.J., Luo, X., Salgueiro, Y., Blackmore, M.G., and Park, K.K. (2015). Viral vector-based improvement of optic nerve regeneration: characterization of individual axons' growth patterns and synaptogenesis in a visual target. Gene Ther. *22*, 811–821. https://doi.org/10.1038/gt.2015.51.

51. Trakhtenberg, E.F., Li, Y., Feng, Q., Tso, J., Rosenberg, P.A., Goldberg, J.L., and Benowitz, L.I. (2018). Zinc chelation and Klf9 knockdown cooperatively promote axon regeneration after optic nerve injury. Exp. Neurol. *300*, 22–29. https://doi.org/10.1016/j.expneurol.2017.10.025.

52. Lukomska, A., Frost, M.P., Theune, W.C., Xing, J., Gupta, M., and Trakhtenberg, E.F. (2024). Nfe2l3 promotes neuroprotection and long-distance axon regeneration after injury in vivo. Exp. Neurol. *375*, 114741. https://doi.org/10.1016/j.expneurol.2024.114741.

53. Park, K.K., Liu, K., Hu, Y., Smith, P.D., Wang, C., Cai, B., Xu, B., Connolly, L., Kramvis, I., Sahin, M., and He, Z. (2008). Promoting axon regeneration in the adult CNS by modulation of the PTEN/mTOR pathway. Science *322*, 963–966. https://doi.org/10.1126/science.1161566.

54. Levkovitch-Verbin, H., Harris-Cerruti, C., Groner, Y., Wheeler, L.A., Schwartz, M., and Yoles, E. (2000). RGC death in mice after optic nerve crush injury: oxidative stress and neuroprotection. Investig. Ophthalmol. Vis. Sci. *41*, 4169–4174.

55. Enright, A.J., John, B., Gaul, U., Tuschl, T., Sander, C., and Marks, D.S. (2003). MicroRNA targets in Drosophila. Genome Biol. *5*, R1. https://doi.org/10.1186/gb-2003-5-1-r1.

56. John, B., Enright, A.J., Aravin, A., Tuschl, T., Sander, C., and Marks, D.S. (2004). Human MicroRNA targets. PLoS Biol. *2*, e363. https://doi.org/10.1371/journal.pbio.0020363.

57. Tran, N.M., Shekhar, K., Whitney, I.E., Jacobi, A., Benhar, I., Hong, G., Yan, W., Adiconis, X., Arnold, M.E., Lee, J.M., et al. (2019). Single-Cell Profiles of Retinal Ganglion Cells Differing in Resilience to Injury Reveal Neuroprotective Genes. Neuron *104*, 1039–1055.e12. https://doi.org/10.1016/j.neuron.2019.11.006.

58. Ashburner, M., Ball, C.A., Blake, J.A., Botstein, D., Butler, H., Cherry, J.M., Davis, A.P., Dolinski, K., Dwight, S.S., Eppig, J.T., et al. (2000). Gene ontology: tool for the unification of biology. The Gene Ontology Consortium. Nat. Genet. *25*, 25–29. https://doi.org/10.1038/75556.

59. Gene Ontology Consortium, Aleksander, S.A., Balhoff, J., Carbon, S., Cherry, J.M., Drabkin, H.J., Ebert, D., Feuermann, M., Gaudet, P., Harris, N.L., Hill, D.P., et al. (2023). The Gene Ontology knowledgebase in 2023. Genetics *224*, iyad031. https://doi.org/10.1093/genetics/iyad031.

60. Inagaki, N., Chihara, K., Arimura, N., Ménager, C., Kawano, Y., Matsuo, N., Nishimura, T., Amano, M., and Kaibuchi, K. (2001). CRMP-2 induces axons in cultured hippocampal neurons. Nat. Neurosci. *4*, 781–782. https://doi.org/10.1038/90476.

61. Leibinger, M., Andreadaki, A., Golla, R., Levin, E., Hilla, A.M., Diekmann, H., and Fischer, D. (2017). Boosting CNS axon regeneration by harnessing antagonistic effects of GSK3 activity. Proc. Natl. Acad. Sci. USA *114*, E5454–E5463. https://doi.org/10.1073/pnas.1621225114.

62. Norsworthy, M.W., Bei, F., Kawaguchi, R., Wang, Q., Tran, N.M., Li, Y., Brommer, B., Zhang, Y., Wang, C., Sanes, J.R., et al. (2017). Sox11 Expression Promotes Regeneration of Some Retinal Ganglion Cell Types but Kills Others. Neuron *94*, 1112–1120.e4. https://doi.org/10.1016/j.neuron.2017.05.035.

63. Wang, Z., Reynolds, A., Kirry, A., Nienhaus, C., and Blackmore, M.G. (2015). Overexpression of Sox11 promotes corticospinal tract regeneration after spinal injury while interfering with functional recovery. J. Neurosci. *35*, 3139–3145. https://doi.org/10.1523/JNEUROSCI.2832-14.2015.

64. Chang, K.C., Bian, M., Xia, X., Madaan, A., Sun, C., Wang, Q., Li, L., Nahmou, M., Noro, T., Yokota, S., et al. (2021). Posttranslational Modification of Sox11 Regulates RGC Survival and Axon Regeneration. eNeuro *8*, ENEURO.0358-20.2020. https://doi.org/10.1523/ENEURO.0358-20.2020.

65. Holmes, F.E., Mahoney, S., King, V.R., Bacon, A., Kerr, N.C., Pachnis, V., Curtis, R., Priestley, J.V., and Wynick, D. (2000). Targeted disruption of the galanin gene reduces the number of sensory neurons and their regenerative capacity. Proc. Natl. Acad. Sci. USA *97*, 11563–11568. https://doi.org/10.1073/pnas.210221897.

66. Jacobi, A., Tran, N.M., Yan, W., Benhar, I., Tian, F., Schaffer, R., He, Z., and Sanes, J.R. (2022). Overlapping transcriptional programs promote survival and axonal regeneration of injured retinal ganglion cells. Neuron *110*, 2625–2645.e7. https://doi.org/10.1016/j.neuron.2022.06.002.

67. Liu, D., Webber, H.C., Bian, F., Xu, Y., Prakash, M., Feng, X., Yang, M., Yang, H., You, I.J., Li, L., et al. (2024). Optineurin-facilitated axonal mitochondria delivery promotes neuroprotection and axon regeneration. Preprint at bioRxiv. https://doi.org/10.1101/2024.04.02.587832.

68. Li, L., Fang, F., Feng, X., Zhuang, P., Huang, H., Liu, P., Liu, L., Xu, A.Z., Qi, L.S., Cong, L., and Hu, Y. (2022). Single-cell transcriptome analysis of regenerating RGCs reveals potent glaucoma neural repair genes. Neuron *110*, 2646–2663.e6. https://doi.org/10.1016/j.neuron.2022.06.022.

69. Belin, S., Nawabi, H., Wang, C., Tang, S., Latremoliere, A., Warren, P., Schorle, H., Uncu, C., Woolf, C.J., He, Z., and Steen, J.A. (2015). Injury-induced decline of intrinsic regenerative ability revealed by quantitative proteomics. Neuron *86*, 1000–1014. https://doi.org/10.1016/j.neuron.2015.03.060.

70. de Lima, S., Koriyama, Y., Kurimoto, T., Oliveira, J.T., Yin, Y., Li, Y., Gilbert, H.Y., Fagiolini, M., Martinez, A.M.B., and Benowitz, L. (2012). Full-length axon regeneration in the adult mouse optic nerve and partial recovery of simple visual behaviors. Proc. Natl. Acad. Sci. USA *109*, 9149–9154. https://doi.org/10.1073/pnas.1119449109.

71. Lim, J.H.A., Stafford, B.K., Nguyen, P.L., Lien, B.V., Wang, C., Zukor, K., He, Z., and Huberman, A.D. (2016). Neural activity promotes long-distance, target-specific regeneration of adult retinal axons. Nat. Neurosci. *19*, 1073–1084. https://doi.org/10.1038/nn.4340.

OMTA, Volume *34*

Supplemental information

Neurodevelopmental miR-135a-1-3p and miR-135b-3p promote long-distance axon regeneration after injury in the central visual pathway

Jacob H. Brady, Agnieszka Lukomska, Arnav J. Balaji, Clyde D'Souza, and Ephraim F. Trakhtenberg

MATERIALS AND METHODS

Animal use, surgeries, intraocular injections. All animal studies were performed at the University of Connecticut Health Center with approval of the Institutional Animal Care and Use Committee and of the Institutional Biosafety Committee, and performed in accordance with the ARVO Statement for the Use of Animals in Ophthalmic and Visual Research. Mice were housed in the animal facility with a 12-h light/12-h dark cycle (lights on from 7:00 AM to 7:00 PM) and a maximum of five adult mice per cage. Food and water were available *ad libitum*. The study used wild-type 129S1/SvImJ mice (The Jackson Laboratory). Optic nerve surgeries and intravitreal injections were carried out on mice of both sexes, 8-12 weeks of age (average body weight 20-26 g) under general anesthesia, as described previously[1-5]. The viruses included AAV2 vectors expressing miR-135a-1-3p-mimic (sequence: 5′-TATAGGGATTGGAGCCGTGGCG-3′), miR-135b-3p-mimic (sequence: 5′-ATGTAGGGCTAAAAGCCATGGG-3′), and scrambled control (sequence: 5’-TCGAGGGCGACTTAACCTTAGG-3’) shRNAs (titers ~1 × 10^{13} GC/mL; VectorBuilder, Inc.). All AAV2 vectors co-expressed an mCherry reporter of transduction. Viruses (2 µl per eye) were injected intravitreally, avoiding injury to the lens, in 8-week-old mice, which were randomly assigned to experimental or control conditions, 2 weeks prior to ONC surgery. It is standard protocol to inject AAV2 two weeks prior to ONC, because this lead time allows sufficient time for transduction and expression of the shRNAs in RGCs at the time of ONC[6] (the timings of treatment and injury are shown in the experimental timeline figures). Transduction efficiency was approximately 30%, which is within a range of prior reports that also used intravitreal AAV2 to target the RGCs[2-4,6-8]. The RP-RGD was manufactured as a custom service by GenScript (Piscataway, NJ), as we previously described[9,10]. Immediately after ONC surgery, silicon oil-based emulsion containing RP-RGD (0.2 mg/ml in PBS) or control PBS were injected intravitreally (while allowing equivalent volume to leak-out upon needle withdrawal after 3 µl contents was slowly released into the eye), avoiding injury to the lens. Emulsion using silicon oil (Silikon 1000, 8065601187, Alcon) was prepared following the methods we previously described[9,10]. Cholera toxin subunit B (CTB) conjugated to Alexa Fluor 488 dye (C34775, ThermoFisher Scientific) was injected (1% in 3 µl PBS) intravitreally one day prior to sacrifice, at 2 or 6 weeks after ONC, in order to visualize the regenerating axons or their absence (although 2 µl of AlexaFluor-conjugated CTB solution is sufficient for labeling axons in the optic nerve, we added 1 µl extra in order to retain strong fluorescent signal intensity even at long distances through the optic chiasm and tract). Investigators performing the surgeries and injections were masked to the group identity until the end of the experiment.

Tissue processing and immunostaining. Standard histological procedures were used, as described previously[1-4,11]. Briefly, anesthetized mice were transcardially perfused with isotonic saline followed by 4% paraformaldehyde (PFA) at 2 or 6 weeks after ONC, the eyes, optic nerves, and optic chiasms were dissected and postfixed 2 hrs. The retinas were dissected-out from the eyes into PBS in 24-well plate at 4 °C. The optic nerves and chiasms were transferred to 30% sucrose overnight at 4 °C. Then, optic nerves and chiasms were embedded in OCT Tissue Tek Medium (Sakura Finetek) and frozen. Optic nerves and chiasms were cryosectioned longitudinally or horizontally, respectively, at 14 µm, and then mounted for imaging on coated glass slides. For analyzing RGC survival, resected (into PBS at 4 °C) free-floating retinas were immunostained in 24-well plate wells and, after making 4 symmetrical slits, flat-mounted on coated glass slides for imaging. For analyzing RGC transduction, the flattened retinas were embedded in OCT Tissue Tek Medium (Sakura Finetek), frozen, and cryosectioned at 14 µm horizontally (capturing the ganglion cell layer of the whole retina), and then immunostained and mounted on coated glass slides for imaging. For immunostaining, the tissues were blocked with appropriate sera, incubated overnight at 4 °C with primary antibody against βIII-Tubulin (1:500; rabbit polyclonal, Abcam, Ab18207), washed 3 times, incubated with appropriate fluorescent dye-conjugated secondary antibody (1:500; IgG H+L; Alexa Fluor, Thermo Fisher Scientific) overnight at 4 °C, washed 3 times again, and mounted for imaging.

Imaging and quantifications of RGC survival/axon regeneration. To visualize the regenerating axons or their absence after treating with the AAV2 vectors expressing miR-135a-1-3p-mimic, miR-135b-3p-mimic, or scrambled control, axonal tracer (Alexa Fluor 488-conjugated CTB 1% in 3 µl PBS) was intravitreally injected one day before animals were euthanized 2 or 6 weeks following ONC. Longitudinal sections of the optic nerve were examined for possible axon sparing[3]. No spared axons were found in control, and no evidence of axon sparing was found in experimental conditions (e.g., at 2 weeks after injury, no axons were found at the most distal from the injury region of the optic nerve). Regenerated axons (defined as continuous fibers, which are absent in controls and are discernible from background puncta and artefactual structures) were counted

manually using a fluorescent microscope (40x/1.2 C-Apochromat W; AxioObserver.Z1, Zeiss) in 4 longitudinal sections per optic nerve at 0.5 mm intervals at increasing distances from the injury site (identified by the abrupt disruption of the densely packed axons near the optic nerve head, as marked by a rhombus in Figures), and these values were used to estimate the total number of regenerating axons per nerve, as described[1-4]. For representative images, serial fields of view along the longitudinal optic nerve or through horizontal optic chiasm tissue cryosections were imaged as above; *z*-stacks with 5 planes at 0.5 µm intervals were deconvoluted, merged, and stitched (ZEN software, Zeiss). The edges of the tissue were optically trimmed (i.e., cropped-out) due to artefactual autofluorescence that is common at tissue edges. Then, processed images of 2 tissue sections from the same optic nerve were superimposed over each other and merged using Photoshop CS6 (Adobe), shown as representative images. Only 1 tissue section was used for horizontal optic chiasm representative images, because differences in size of optic chiasm's horizontal sections make it difficult to superimpose them in Adobe Photoshop CS6. RGC survival was quantified in retinal flatmounts' GCL as described[1-4,11], by immunostaining with an antibody to βIII-Tubulin (neuronal marker), taking advantage of the βIII-tubulin's selective expression in RGCs within the retina's GCL. ImageJ software Cell Counter Plugin was used to count βIII-Tubulin positive cells from images acquired (using a fluorescent microscope, 20x LD; Zeiss, AxioObserver.Z1, Zeiss) at 1-2 mm from the optic nerve head in four directions of the GCL, then averaged to estimate overall RGC survival per mm^2. For visualization of transduction, retinal regions randomly sampled from the horizontally cryosectioned flattened retinas' GCL immunostained for βIII-Tubulin were imaged, along with the mCherry reporter, using a confocal microscope (63x Oil; LSM800, Zeiss). Investigators performing the surgeries and quantifications were masked to the group identity by another researcher until the end of the experiment.

Primary neurons isolation, semi-quantitative PCR (sqPCR), and Sanger sequencing. Retinas were resected from both sexes of 5 mice of the respective ages: Embryonic day 18 (E18), postnatal day 5 (P5), or 10-12-week adult. RGCs were isolated from retinal single cell suspension by Thy1 immunopanning (CD90, MCA02R, Serotec) after depletion of macrophages (anti-mouse macrophage antibody, AIA31240, Accurate Chemical) and washing off the nonadherent cells, as previously described[7,8,12-14], with the following modifications for adult tissue: digestion in papain decreased to 10 minutes, amount of papain increased by 25%, only one macrophage depletion plate used for 20 minutes, amacrine cell depletion plate (VC1.1, C6680-100TST, Sigma) was used for 20 minutes, and Thy1 panning dish shaken more stringently, also as we previously described[8,15,16]. Motor cortex projection neurons were isolated from single cell suspension of resected motor cortex gray matter of P5 or 10-week-old mice of both sexes by Thy1 immunopanning after depletion of macrophages, as above. RNA was isolated from pelleted cells using the Quick-RNA Microprep Kit (ZR1050, Zymo Research). RNA concentration was measured with NanoPhotometer NP80 (Implen), and 100 ng of total RNA from each sample was used as input for library preparations according to the manufacturer's instructions, as follows. Equivalent concentrations and quantities of total RNA were used as input for mRNA cDNA library using iScript cDNA synthesis kit (Bio-Rad) or for small-RNA cDNA library using NEBNext Multiplex Small RNA Sample prep kit (E7330S, NEB). Spike-in control was used in preparation of small-RNA cDNA library for miRNA sqPCR following methods we described previously[8], as follows: 5.5 µl of total RNA (100 ng total RNA) was mixed with 0.5 µl of *ath-miR775* miRNA mimic (5'-UUCGAUGUCUAGCAGUGCCA-3'; at 0.625 ng/µl) concentration after 1:800 dilution from stock solution (10620310, custom RNA with phosphate 5' modification, ThermoFisher Scientific). Spike-in with *ath-miR775* miRNA mimic was used for normalization of expression across library replicates and timepoints/conditions for comparative analysis. All subsequent steps of small-RNA library prep were performed according to the manufacturer protocol (E7330S, NEB), through purification of the cDNA library using the Monarch PCR and DNA Cleanup Kit (T1030S, NEB). Libraries were eluted in 30 µl of nuclease-free water. For sqPCR, 2 µl of library was used as input in a 50 µl PCR reaction using Q5 Polymerase (M0491S, NEB). All samples were prepared using a single PCR master mix (buffer, water, universal reverse primer, dNTPs and polymerase). The universal reverse primer (5'-CAAGCAGAAGACGGCATACGAGA-3') is specific to the 3' adaptor from the NEB small-RNA library kit (E7330S), as we previously described and validated[8]. Then, 2 µl of template along with 2.5 µl of miRNA-specific primer (either for a target miR-135a-1-3p 5'-TATAGGGATTGGAGCCGTGG-3', or miR-135b-3p 5'-ATGTAGGGCTAAAAGCCATGGG-3') or spike-in control (*ath*-miR-775) were added to each sample, and PCR was run for 34 cycles on a BioRad T100 Thermal Cycler. After PCR, samples were electrophoresed on a 1.2% agarose TAE gel, along with DNA ladder. SYBR Safe DNA Gel Stain (S33102, Thermo Fisher Scientific) was used for visualization of bands in the gels, which were imaged using the BioRad Universal Hood II, and images were exported in TIFF format. Gel band intensities in the images were quantified using ImageJ software. Three biological replicates per condition were used. Intensity of the gel

band, matching the expected size of the amplified target miRNA with adaptors, was normalized to spike-in control band intensity, and then normalized by sum across replicates. Gel bands matching the expected size of the amplified target miRNA with adaptor were excised, sqPCR products were purified using the Zymoclean Gel DNA Recovery Kit (D4001, Zymo Research), and the purified products were Sanger-sequenced (QuintaraBio, Farmington, CT). The identified sequences were aligned to the genome (mm39) and blasted against the mouse miRbase database. For PCR of mRNA cDNA library, 1 µl of each sample was used as a template for 32-cycles PCR reaction using iQ SYBR Green Supermix with the iTaq DNA polymerase (Bio-Rad), performed on the iCycler iQ5 (Bio-Rad) with primers for the following genes: Tubb3 (forward: 5'-TAGACCCCAGCGGCAACTAT-3'; reverse: 5'-GTTCCAGGTTCCAAGTCCACC-3')[17]; Thy1 (forward: 5'-CTTGCAGGTGTCCCGAGGGC-3'; reverse: 5'-ATGGGATTCGCGCCCGAGAC-3')[18]; and Actb housekeeping control for which cDNA template was diluted 100x (forward: 5'-TGACGTTGACATCCGTAAAG-3'; reverse: 5'-GAGGAGCAATGATCTTGATCT-3')[19]. Standard no-template and no-RT controls were used. Samples (10 µl of RT-PCR products) were electrophoresed on a 1.5% agarose gel with TAE, along with 50 bp DNA ladder (NE3236S, NEB). SYBR Safe DNA Gel Stain (S33102, Thermo Fisher Scientific) was used for visualization of bands in the gels. Gel images were acquired using the BioRad Universal Hood II and exported in tiff format.

Primary neurons culture. P5 and P70 motor cortex neurons isolated by Thy1 immunopanning (as described in the previous section) were cultured in 48-well tissue culture plates (Falcon), coated with poly-D-lysine (10 mg/mL) and fibronectin (20 µg/mL; 1030FN01M, R&D Systems), in a defined medium which we described previously for RGC culture[9], with the exception that FGF2 (0.1 µg/mL; BT-FGFB, R&D Systems) was added, as adult neurons survive poorly in culture. At 5 DIC, cells were fixed for immunostaining with an antibody to βIII-Tubulin (1:500; mouse IgG2a, MMS-435P, BioLegend) and appropriate fluorescent dye-conjugated secondary antibodies (1:500; AlexaFluor, Thermo Fisher Scientific), and then imaged with a fluorescent microscope (20x LD; Zeiss, AxioObserver.Z1, Zeiss), using the methods we described previously[9].

RGC bulk-RNA-seq and scRNA-seq. All bulk small-RNA-seq and mRNA-seq samples from different age RGCs were purified by immunopanning for Thy1 (after immunopanning depletion of macrophages and amacrine cells, as described above). For bulk-mRNA-seq of adult RGCs transduced with AAV2 (~30% transduction efficiency) expressing miR-135a-1-3p-mimic, miR-135b-3p-mimic, or scramble-control shRNA, RGCs were Thy1-immunopanned from single cell retinal suspension (from 10 post-ONC retinas per condition) and FACS'ed for mCherry+ cells, as we described previously[8,16]. Approximately, 5,000 RGCs (per condition) were collected by FACS and RNA was isolated immediately using the Quick-RNA Microprep Kit (ZR1050, Zymo Research). Total RNA with RNA Integrity Number (RIN) ≥ 9 (by Bioanalyzer 2100 using the Nano 6000 kit, Agilent) was extracted using Direct-zol RNA MiniPrep kit (R2050, Zymo Research). For mRNA-seq, cDNA libraries were prepared using polyA-selected RNA (TruSeq RNA Library Prep Kit, Illumina), paired reads sequenced 100 bp from each end on HiSeq 2000 Sequencer (Illumina), passed QC filters, mapped to the mm39 genome and transcriptome by Hisat2, and analyzed by the Cufflinks package as we previously described[8,14,20], with the following modifications: Transcript abundance was determined by Cuffquant based on the mm39 genome and differential expression analyzed by CuffDiff using default parameters. For small-RNA-seq, approximately 200,000 of Thy1-immunopanned RGCs were collected (from each condition) and RNA was isolated immediately for preparing cDNA libraries using adaptors that ligate to the endogenously modified 5' and 3' ends of processed small-RNAs, thereby enriching for small-RNAs such as miRNAs, while excluding degraded RNA fragments, precursors, and other RNA species (TruSeq Small RNA Kit, Illumina). Small-RNA cDNA library products in the range of 140-160 base pairs (including 5' and 3' adapters and an insert corresponding to a small-RNA) were sequenced as paired reads 100 bp from each end on HiSeq 2000 Sequencer (Illumina), passed QC filters, and mapped to the mm39 genome and transcriptome, using Bowtie with default parameters. The resulting binary alignment map (BAM) files were filtered to retain only the full-length (i.e., limited by an miRNA size) reads with 100% alignment and then randomly subsampled, using Samtools, to a similar number of total reads in each sample for comparative analysis. The filtered reads' FASTQ were then realigned (with 1 mismatch allowed, multimapping disabled, and end-to-end Bowtie parameters) to a miRbase miRNA transcripts reference customized to remove the miR-135a-5p (in order for its respective reads to align to the miR-135b-5p with 1 mismatch allowed for the combined miR-135a/b-5p net expression) and quantified/analyzed using salmon packages[21] (mapping based mode) and DESeq2[22]. For alignments visualizations using IGV viewer (Broad Institute)[23] in Fig. 1C, the filtered reads were also realigned (with 1 mismatch allowed, multimapping enabled, and end-to-end Bowtie parameters) to a mm39 genome reference. For miR-135a/b-5p net reads alignment visualization, the miR-135a-1-5p locus shown in Fig.

1C also includes miR-135a-2-5p and miR-135b-5p reads that aligned to it. MiRNAs' TPM values (from the salmon quant.sf) for replicates were averaged in each age/condition, and shown on *x*-axis of the DESeq2 differential abundance plot in Fig. 1D. Previously generated by us and others RGC scRNA-seq datasets used in this study are available through the NCBI Gene Expression Omnibus (GEO) accession numbers provided in the Data Availability section below. Datasets were merged and normalized using Seurat v5.2.0[24,25]. RGC clusters assignments (in Figure S2H) were as described previously[16,26], and bubble plot (in Figure S2H) indicating RGC subtypes' normalized gene expression and proportion of RGCs per subtype expressing the gene was generated using the R package ggplot2[27].

miRNA targets prediction. miRNA target prediction analysis was performed on genes expressed in adult injured RGCs scRNA-seq dataset[26] at normalized expression (*NE*) of at least 0.5 *NE* (this minimal *NE* threshold was set in order to filter-out genes expressed at near-noise levels as they are unlikely to present axon regeneration-suppressing targets). The selected injured RGC genes were then cross-referenced to the dataset of transcript-specific bulk-mRNA-seq-profiled ONC-injured RGCs (which we generated as described below, GEO accession # GSE329865) to identify respective transcript isoforms that are expressed at least 0.5 *FPKM* in each of the 2 replicates. miRNA target prediction analysis was performed on 3' UTR sequences of these transcripts. The 3' UTRs of the selected annotated RGC transcripts were extracted using a custom Bash script along with the modules BEDtools 2.29.0, Bedops 2.4.35, and Gffread 0.12.7. Then, extracted 3' UTRs (**Table S1**) and fasta files of the miRNA sequences were used as inputs for miRanda 3.3a with default parameters[28,29]. The output from miRanda was parsed using Perl to obtain a table of predicted targets and their associated miRanda target prediction scores (Figure S2A-G).

Gene annotation/ontology enrichment analysis was performed on genes differentially expressed (Cuffdiff *q*-value < 0.05) in bulk-mRNA-seq-profiled injured RGCs after either miR-135a-1-3p-mimic or miR-135b-3p-mimic treatment relative to scrambled-control shRNA treatment, that were further filtered based on at least 2 log2FC regulation relative to scrambled-control shRNA-treated uninjured RGCs and the higher expression condition expressing the gene at least 5 FPKM. Due to potential contamination with non-RGC retinal cell-types, only genes established to be expressed (at least 0.01 *NE*) in any RGC state (e.g., embryonic, postnatal, adult, injured, or regenerative, based on our and others' prior scRNA-seq studies[14,30-32]) were retained for analysis. Gene annotation/ontology enrichment analysis was performed using the GO:BP terms database and the R packages *gprofiler2*, *enrichplot*, and *clusterProfiler*[33-37]. All genes expressed in adult uninjured RGCs (FPKM ≥ 0.1) based on the dataset we generated previously[8] were used as background. DEG-associated GO:BP terms were identified using *gprofiler2*'s gost function with the g:SCS method for multiple testing correction and a significance threshold of $p < 0.05$. For each GO term, fold-enrichment was calculated as the proportion of query genes associated with the GO term divided by the proportion of background genes associated with the GO term. GO terms with log2 fold-enrichment > 0.8 were retained, and semantically redundant terms were removed using the simplify function ('cutoff = 0.8'). Genes comprising significantly enriched annotation/ontology terms were analyzed by a gene network plot using the *enrichplot* R package cnetplot function[35,37].

Experimental design and statistical analyses. All tissue processing, quantification, and data analysis were done masked throughout the study. Sample sizes were based on accepted standards in the literature and our prior experiences. Sample size (*n*) represents total number of biological replicates in each condition. All experiments included appropriate controls. No cases were excluded in our data analysis, although a few animals that developed a cataract in the injured eye were excluded from the study, and their tissues were not processed. The data are presented as means (SEM) and was analyzed (as specified in the respective Figure legends), where appropriate, by independent samples *t*-test 2-tailed, or one-way ANOVA with or without Repeated Measures, and a posthoc LSD test (SPSS), as indicated. All differences were considered significant at $p < 0.05$.

Data availability. RGC bulk small-RNA-seq processed raw reads and data for miRNA 135 family miRNA loci analyzed in this study are available through the NCBI Sequence Read Archive (SRA) under accession # PRJNA1483696. Bulk-mRNA-seq processed raw reads and data for injured adult RGCs treated with miR-135a-1-3p-mimic or miR-135b-3p-mimic are available through the NCBI GEO under accession # GSE337069 and for treated with scramble-control shRNA under accession # GSE329865[38]. The adult uninjured RGC bulk-mRNA-seq dataset used in this study, which we previously generated, is available through the NCBI GEO under accession # GSE252517[8]. RGC scRNA-seq datasets used in this study, which were previously generated by us and others, are available through the NCBI GEO accession # GSE122466[30], GSE325128[32], GSE115404[14], GSE137400[26], and GSE202155[31].

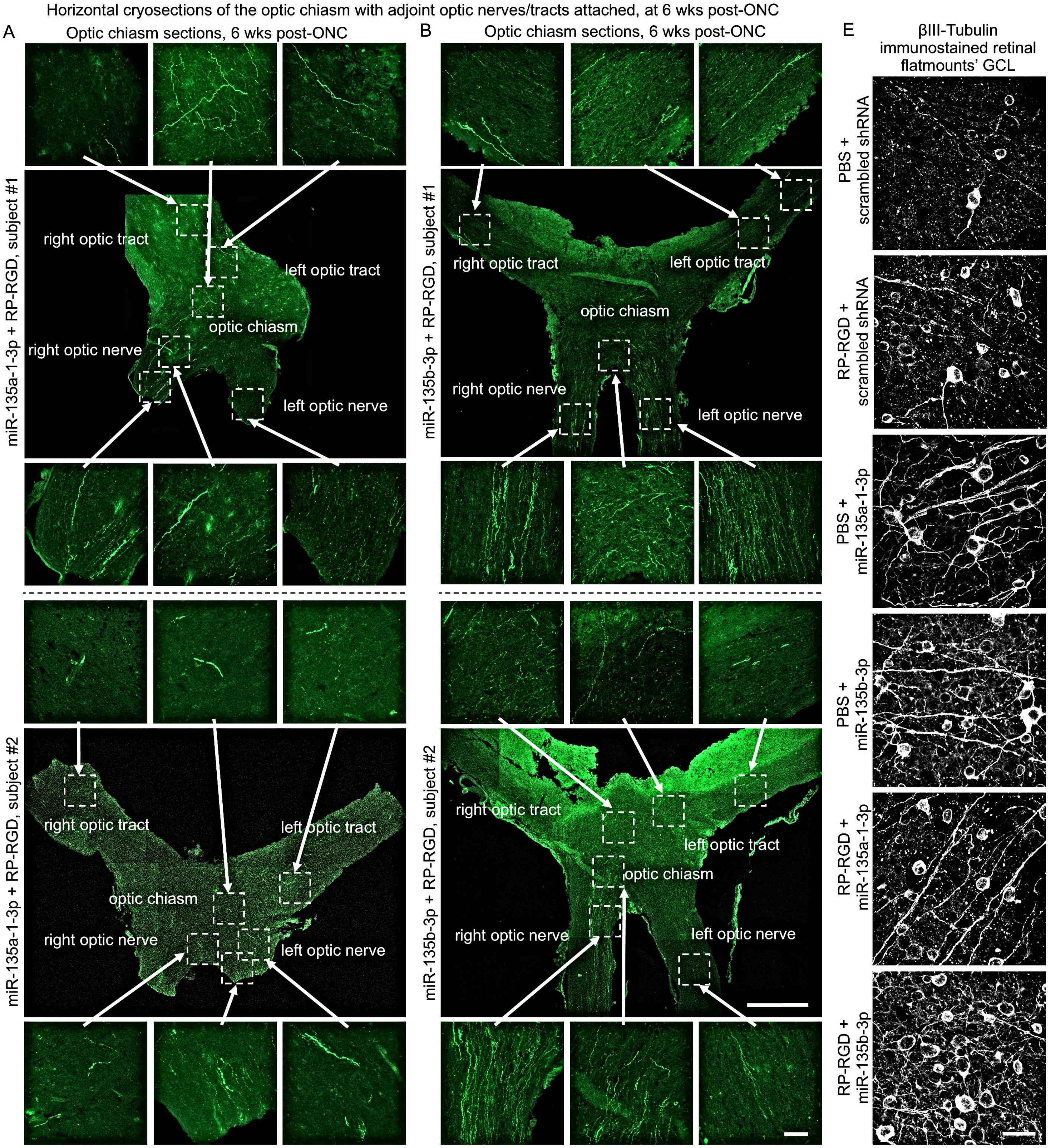

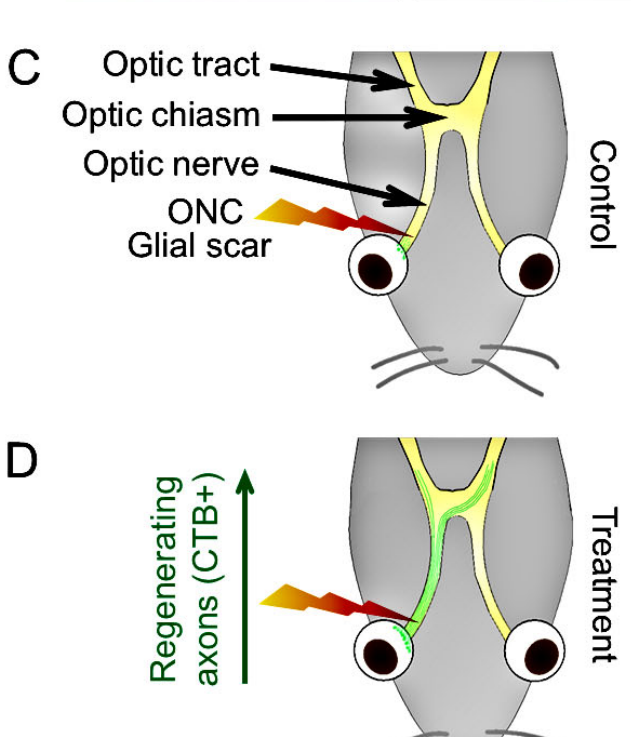

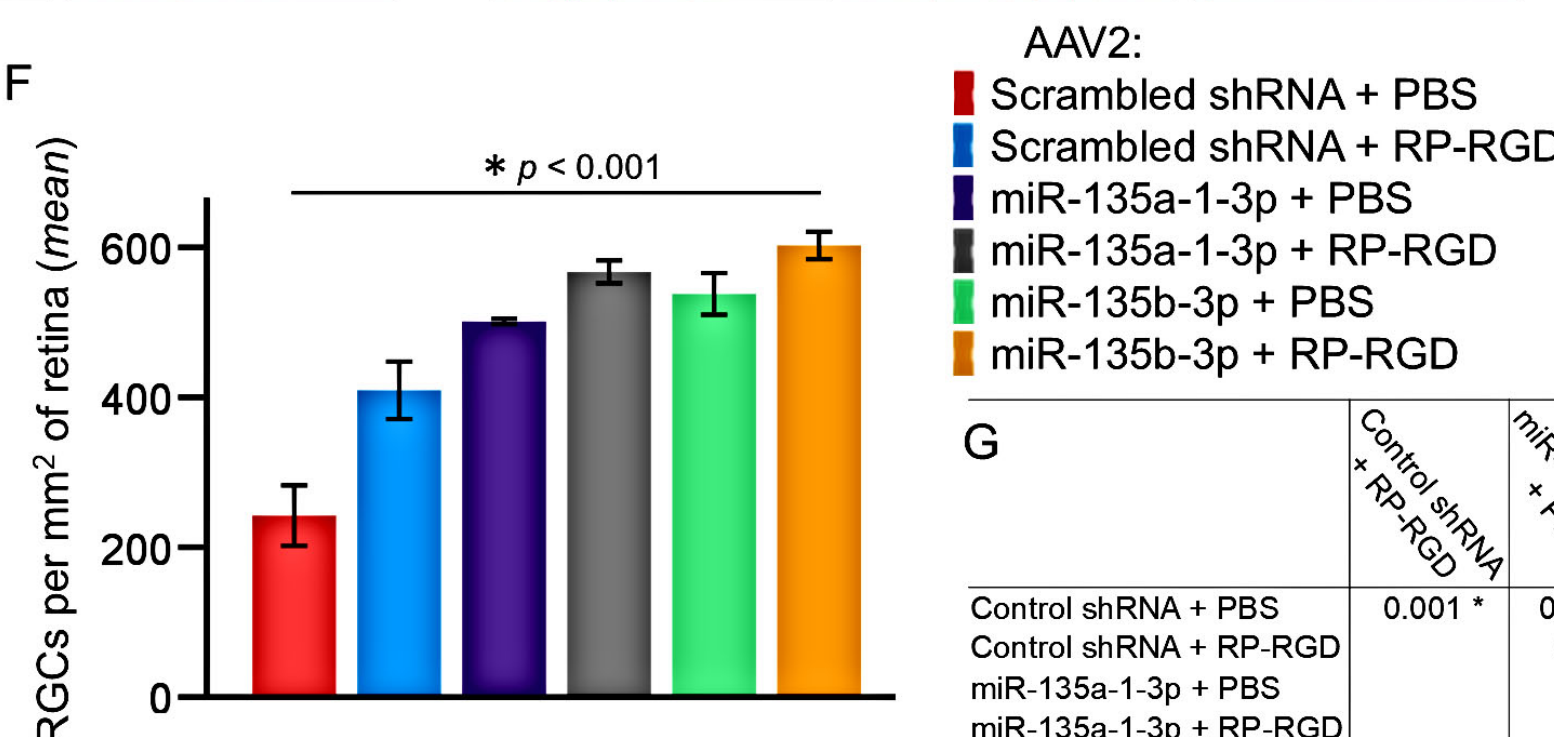

G

	Control shRNA + RP-RGD	miR-135a-1-3p + PBS	miR-135a-1-3p + RP-RGD	miR-135b-3p + PBS	miR-135b-3p + RP-RGD
Control shRNA + PBS	0.001 *	0.001 *	0.001 *	0.001 *	0.001 *
Control shRNA + RP-RGD		*N.S.*	0.002 *	0.010 *	0.001 *
miR-135a-1-3p + PBS			*N.S.*	*N.S.*	0.054 *
miR-135a-1-3p + RP-RGD				*N.S.*	*N.S.*
miR-135b-3p + PBS					*N.S.*

FIGURE S1

Figure S1. Axons regenerating in response to AAV2 miR-135a-1-3p or miR-135b-3p mimics co-treated with RP-RGD can reach the optic tract by 6 weeks post-injury.

(**A-B**) Experimental timeline shown in Fig. 3A. Representative images of horizontal cryosections of the optic chiasm with adjoint optic nerves/tracts segments attached, as marked, from the animals bilaterally injured and treated as marked. Images from two different animals with most regenerating axons detected by 6 weeks post-ONC are shown for co-treatments with RP-RGD and AAV2 miR-135a-1-3p (*A*) or miR-135b-3p (*B*). Insets: Representative CTB-labeled axons detected in segments of the central visual pathway (outlined by dashed boxes in *A-B*), as marked. There were no axons detected in the control condition (not shown), consistent with that no regenerating axons were detected even in the preceding segments of the central visual pathway, as absence of axons is apparent in the optic nerves from control conditions shown in Fig. 3. Scale bars, 500 µm (main panels), 20 µm (insets).

(**C-D**) To assist in the interpretation of the histological images, a schematic of failed axon regeneration in control shows the central visual pathway segments through the optic tract, along with ONC site, as marked (*C*), and a representation of axon regeneration reaching the optic tract achieved by the co-treatments at 6 weeks post-ONC is shown for comparison to control (*D*).

(**E-G**) Representative images of the retinal flatmounts' GCL immunostained for an RGC marker βIII-Tubulin at 6 weeks post-ONC, from the conditions pre-treated with AAV2 expressing miR-135a-1-3p-mimic, miR-135b-3p-mimic, or scrambled-control shRNA, and co-treated with RP-RGD or vehicle, as marked. Scale bar, 20 µm (*E*). Quantitation of RGC (βIII-Tubulin+ cells) survival in retinal flatmounts at 6 weeks post-ONC, from the treatment conditions as marked (*F*). Data analyzed using ANOVA, overall $F = 19.95$, $p < 0.001$, with p-values of pairwise comparisons determined by posthoc LSD shown in the table, and significant differences ($p < 0.05$) indicated by an asterisk * (*G*). Mean (SEM) shown; $n = 4$ cases per group.

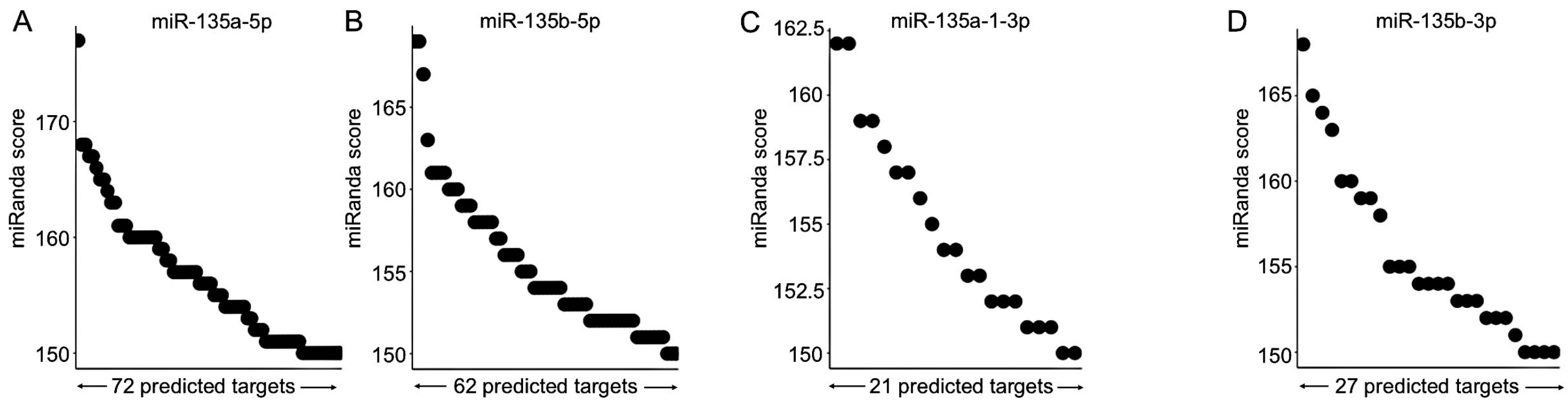

E

Gene	Transcript ID	miR-135a-5p Score	miR-135b-5p Score
Pbx1	ENSMUST00000176540	177	169
Zfp385b	ENSMUST00000111830	168	160
Eif4g2	ENSMUST00000162415	167	159
Kitl	ENSMUST00000105283	167	167
Hdac9	ENSMUST00000209902	166	158
Anks1b	ENSMUST00000182356	165	169
Robo2	ENSMUST00000117785	165	157
Klf4	ENSMUST00000107619	164	156
Rora	ENSMUST00000034766	163	155
Zwint	ENSMUST00000105431	163	155
Cpne8	ENSMUST00000088649	161	161
Rps20	ENSMUST00000138502	161	161
Trpm3	ENSMUST00000237357	161	161
Akap6	ENSMUST00000219786	160	152
Ank3	ENSMUST00000182665	160	152
Hcn1	ENSMUST00000006991	160	152
Itm2b	ENSMUST00000022704	160	160
Pde10a	ENSMUST00000115717	160	152
App	ENSMUST00000227723	159	163
Srgap1	ENSMUST00000020322	159	151
Snrpn	ENSMUST00000059305	158	158
Epha5	ENSMUST00000053733	157	150
Tnik	ENSMUST00000159236	157	161
Ywhae	ENSMUST00000067664	157	157
Gpr158	ENSMUST00000055946	156	152
Rpl5	ENSMUST00000082223	156	156
Strbp	ENSMUST00000028279	156	152
Fam171b	ENSMUST00000051454	155	151
Nrxn3	ENSMUST00000110130	155	159
Ext1	ENSMUST00000077273	154	154
H3f3a	ENSMUST00000161308	154	154
Mmp16	ENSMUST00000029881	154	158
Pde4b	ENSMUST00000106907	154	154
Nrg1	ENSMUST00000207470	152	156
Ppfia2	ENSMUST00000217854	152	156
B3galt1	ENSMUST00000112346	151	151
Zfand5	ENSMUST00000237651	151	155
Tcf25	ENSMUST00000211932	150	154

F

Gene	Transcript ID	miR-135a-1-3p Score
Kcnma1	ENSMUST00000145596	162
Zbtb20	ENSMUST00000114694	162
Dcc	ENSMUST00000114943	159
Pbx1	ENSMUST00000176540	159
Msi2	ENSMUST00000107909	158
Map2	ENSMUST00000024639	157
Eif4g2	ENSMUST00000162415	156
Pcdh11x	ENSMUST00000113358	155
Gpr158	ENSMUST00000055946	154
Inpp4b	ENSMUST00000172031	154
Tmtc2	ENSMUST00000061506	153
Il1rapl1	ENSMUST00000113966	153
Ywhaq	ENSMUST00000135088	152
Pcdh15	ENSMUST00000191854	152
Tafa1	ENSMUST00000075080	151
Kcnh7	ENSMUST00000075052	151
Robo2	ENSMUST00000117785	150
Dscam	ENSMUST00000056102	150

G

Gene	Transcript ID	miR-135b-3p Score
Fgf14	ENSMUST00000095529	168
Gabrg3	ENSMUST00000068911	165
Pten	ENSMUST00000249247	164
Zbtb20	ENSMUST00000114694	163
Celf2	ENSMUST00000170438	160
Eif4g2	ENSMUST00000162415	159
Kcnma1	ENSMUST00000145596	159
Tenm4	ENSMUST00000107166	158
Anks1b	ENSMUST00000182907	155
Gpx4	ENSMUST00000105372	155
Cdh12	ENSMUST00000075132	154
Camk2d	ENSMUST00000200171	154
Khdrbs2	ENSMUST00000188257	153
Neto1	ENSMUST00000058829	153
Atp5g1	ENSMUST00000090541	153
Magi1	ENSMUST00000089317	152
Map1b	ENSMUST00000064762	151
Zfand5	ENSMUST00000237651	150
Nnat	ENSMUST00000153739	150

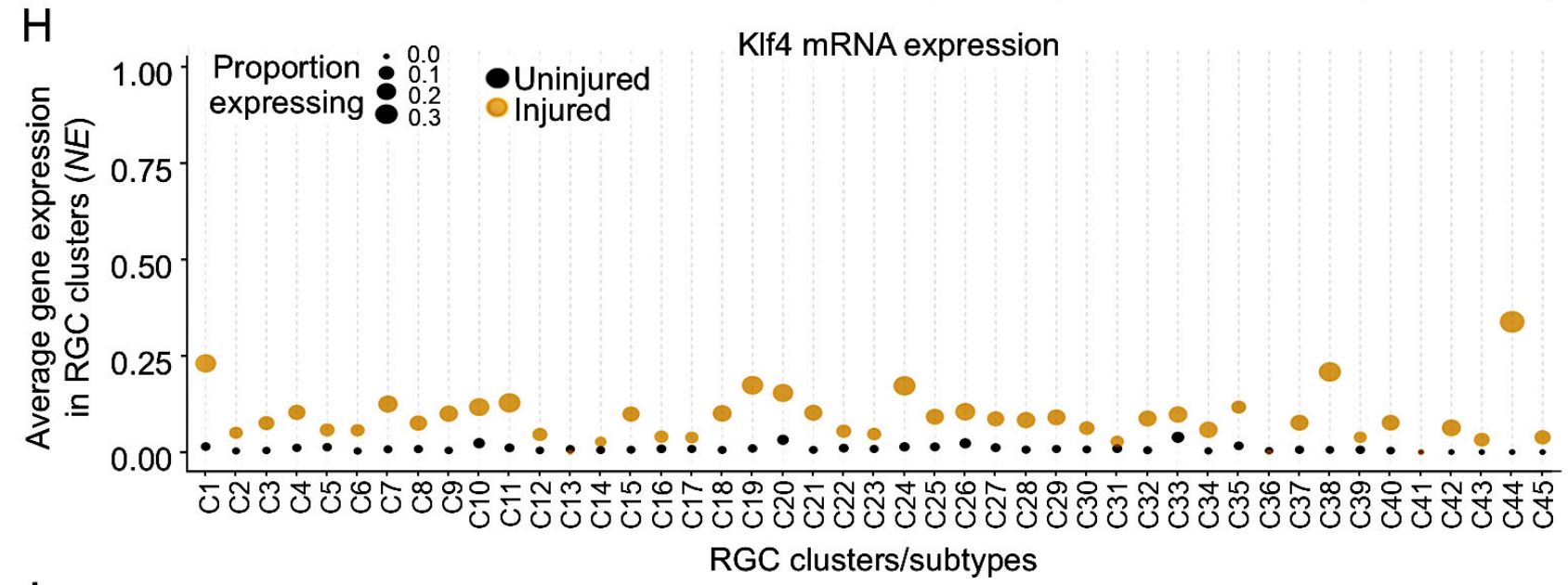

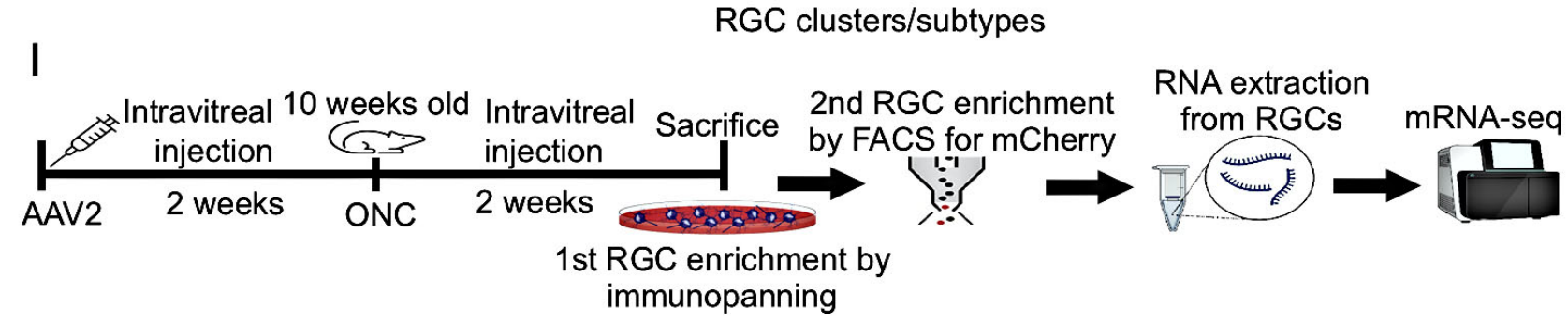

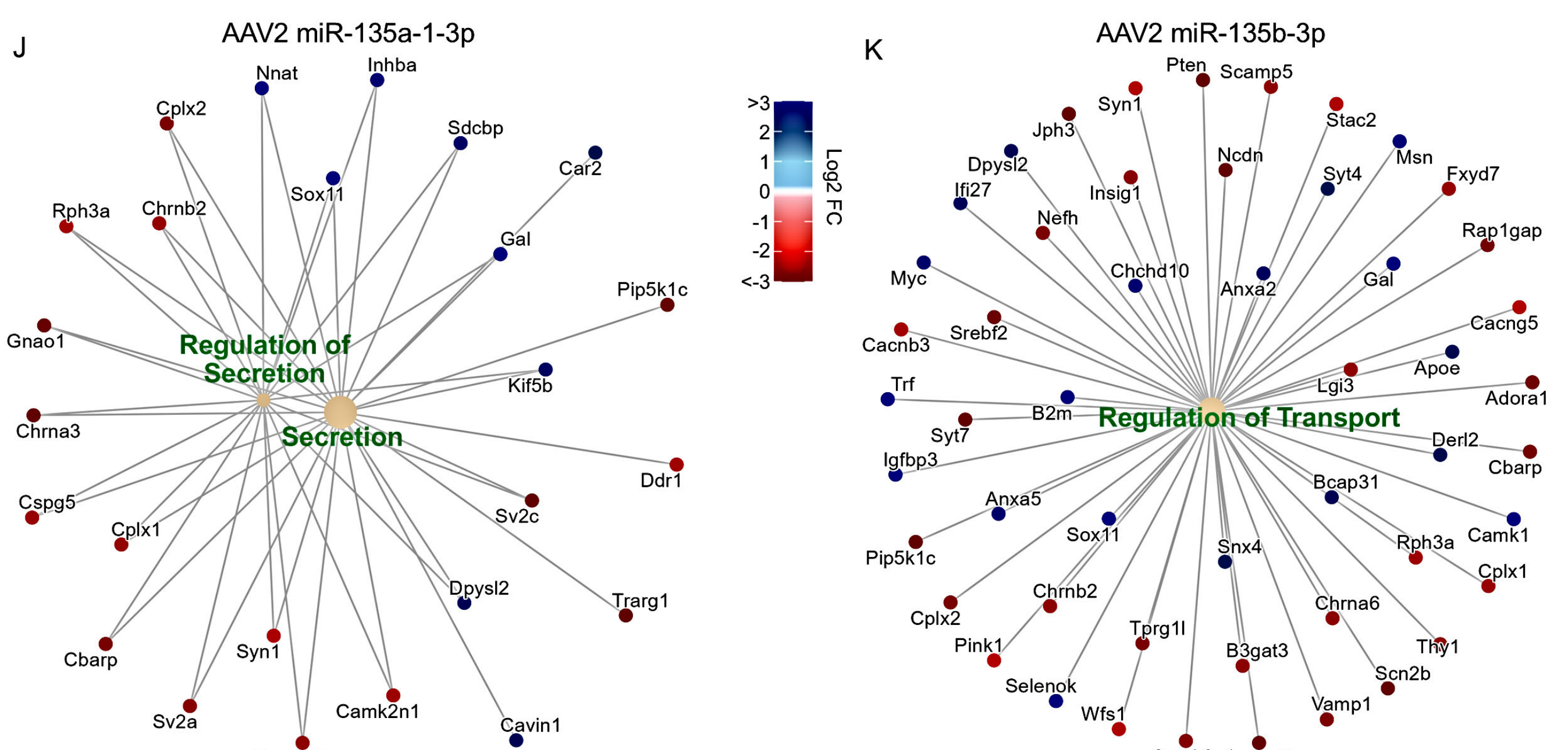

FIGURE S2

Figure S2. miR-135 family miRNAs' predicted targets and genes networks affected by miR-135a-1-3p and miR-135b-3p mimics in injured RGCs.

(**A-D**) Plot of mRNAs (*n*) expressed in injured RGCs vs. respective miRNA's predicted miRanda targeting score, shown for miR-135a-5p (*A*), miR-135b-5p (*B*), miR-135a-1-3p (*C*), and miR-135b-3p (*D*), ordered by prediction score (with higher score indicating higher targeting probability); see Methods.

(**E-G**) The top predicted (score ≥ 150) target genes, along with transcript IDs (one per gene with the highest score shown) and miRanda scores, are shown for the miR-135 family members, as marked. Majority of miR-135a-5p and the differing by 1 bp miR-135b-5p targets are shared, with miR-135a-5p targeting 81% of genes targeted by miR-135b-5p and miR-135b-5p targeting 70% of genes targeted by miR-135a-5p (*E*). Targets shared between the 3p miRNAs are highlighted in blue (*F-G*), with a minor overlapping subset also highlighted for 5p miRNAs. Klf4, highlighted in red, is a predicted target of miR-135a/b-5p, but not of miR-135b-3p or miR-135a-1-3p (see Methods).

(**H**) Bubble plot of Klf4 expression in scRNA-seq RGC datasets[26] suggest only modest (bordering noise) Klf4 levels in the injured (2-wks post-ONC) RGC subtypes. Bubble size scalebar indicates the cluster proportion of RGCs with Klf4 detected at *NE* > 0. *NE* = normalized expression.

(**I**) Experimental timeline: 8-week-old mice were pre-treated with AAV2 expressing miR-135a-1-3p-mimic, miR-135b-3p-mimic, or scrambled-control shRNA, and co-expressing mCherry reporter. ONC injury was performed 2 weeks later, and mCherry+ RGCs were isolated 2 weeks post-ONC for bulk-mRNA-seq analysis. A different, uninjured group was processed similarly (see Methods).

(**J-K**) GO-based gene-concept network plots of the biological processes' terms significantly enriched in the differentially expressed genes in miR-135a-1-3p (*J*) and miR-135b-3p (*K*) treated injured RGCs relative to scrambled-control shRNA-treated injured and uninjured RGCs (see Methods). GO terms are shown as brown circles (i.e., nodes), with circle size (per scale on the side) indicating the number of genes within that node. The color-coded scale bar indicates each gene's log2 fold-change (FC) in expression between the miR-135a-1-3p or miR-135b-3p and scrambled-control shRNA treated injured RGCs.

SUPPLEMENTAL DATA LEGENDS

Table S1. 3' UTR sequences in FASTA format for transcripts isoforms determined, based on RNA-seq analysis, as expressed in ONC-injured RGCs. Refer to the main text and methods for technical details.

Table S2. List of differentially expressed genes (DEGs) with expression and fold-change (FC) values. Log2 FC (log2FC) between the average expression in miR-135a-1-3p or miR-135b-3p mimic-treated injured RGCs and either scrambled-control shRNA-treated injured or uninjured RGCs is shown. Significantly (CuffDiff *q*-value < 0.05) differentially expressed genes (DEGs) between scrambled-control shRNA-treated injured RGCs, that are also at least 2 log2FC relative to uninjured RGCs and with ≥ 5 FPKM in the higher-expressing condition, are shown with the columns annotated as indicated. Refer to the main text and methods for technical details.

SUPPLEMENTAL REFERENCES

1. de Lima, S., Koriyama, Y., Kurimoto, T., Oliveira, J.T., Yin, Y., Li, Y., Gilbert, H.Y., Fagiolini, M., Martinez, A.M., and Benowitz, L. (2012). Full-length axon regeneration in the adult mouse optic nerve and partial recovery of simple visual behaviors. Proc Natl Acad Sci U S A *109*, 9149-9154. 10.1073/pnas.1119449109.
2. Trakhtenberg, E.F., Li, Y., Feng, Q., Tso, J., Rosenberg, P.A., Goldberg, J.L., and Benowitz, L.I. (2018). Zinc chelation and Klf9 knockdown cooperatively promote axon regeneration after optic nerve injury. Exp Neurol *300*, 22-29. 10.1016/j.expneurol.2017.10.025.
3. Kim, J., Sajid, M.S., and Trakhtenberg, E.F. (2018). The extent of extra-axonal tissue damage determines the levels of CSPG upregulation and the success of experimental axon regeneration in the CNS. Sci Rep *8*, 9839. 10.1038/s41598-018-28209-z.
4. Lukomska, A., Kim, J., Rheaume, B.A., Xing, J., Hoyt, A., Lecky, E., Steidl, T., and Trakhtenberg, E.F. (2021). Developmentally upregulated Transcriptional Elongation Factor A like 3 suppresses axon regeneration after optic nerve injury. Neurosci Lett, 136260. 10.1016/j.neulet.2021.136260.
5. Lukomska, A., Frost, M.P., Theune, W.C., Xing, J., Gupta, M., and Trakhtenberg, E.F. (2024). Nfe2l3 promotes neuroprotection and long-distance axon regeneration after injury in vivo. Exp Neurol, 114741. 10.1016/j.expneurol.2024.114741.
6. Yungher, B.J., Luo, X., Salgueiro, Y., Blackmore, M.G., and Park, K.K. (2015). Viral vector-based improvement of optic nerve regeneration: characterization of individual axons' growth patterns and synaptogenesis in a visual target. Gene Ther *22*, 811-821. 10.1038/gt.2015.51.
7. Trakhtenberg, E.F., Wang, Y., Morkin, M.I., Fernandez, S.G., Mlacker, G.M., Shechter, J.M., Liu, X., Patel, K.H., Lapins, A., Yang, S., Dombrowski, S.M., et al. (2014). Regulating Set-β's Subcellular Localization Toggles Its Function between Inhibiting and Promoting Axon Growth and Regeneration. J Neurosci *34*, 7361-7374. 10.1523/JNEUROSCI.3658-13.2014.
8. Lukomska, A., Theune, W.C., Frost, M.P., Xing, J., Kearney, A., and Trakhtenberg, E.F. (2024). Upregulation of developmentally-downregulated miR-1247-5p promotes neuroprotection and axon regeneration in vivo. Neurosci Lett *823*, 137662. 10.1016/j.neulet.2024.137662.
9. Lukomska, A., Rheaume, B.A., Frost, M.P., Theune, W.C., Xing, J., Damania, A., and Trakhtenberg, E.F. (2024). Augmenting fibronectin levels in injured adult CNS promotes axon regeneration in vivo. Exp Neurol *379*, 114877. 10.1016/j.expneurol.2024.114877.
10. Lukomska, A., Frost, M.P., Brady, J., Kearney, A., Balaji, A.J., D'Souza, C., and Trakhtenberg, E.F. (2026). Experimental upregulation of Lancl1 promotes axon regeneration after optic nerve injury in vivo. Exp Neurol *402*, 115789. 10.1016/j.expneurol.2026.115789.
11. Kurimoto, T., Yin, Y., Omura, K., Gilbert, H.Y., Kim, D., Cen, L.P., Moko, L., Kügler, S., and Benowitz, L.I. (2010). Long-distance axon regeneration in the mature optic nerve: contributions of oncomodulin, cAMP, and pten gene deletion. J Neurosci *30*, 15654-15663. 10.1523/JNEUROSCI.4340-10.2010.
12. Meyer-Franke, A., Kaplan, M.R., Pfrieger, F.W., and Barres, B.A. (1995). Characterization of the signaling interactions that promote the survival and growth of developing retinal ganglion cells in culture. Neuron *15*, 805-819.
13. Trakhtenberg, E.F., Pita-Thomas, W., Fernandez, S.G., Patel, K.H., Venugopalan, P., Shechter, J.M., Morkin, M.I., Galvao, J., Liu, X., Dombrowski, S.M., and Goldberg, J.L. (2016). Serotonin receptor 2C regulates neurite growth and is necessary for normal retinal processing of visual information. Dev Neurobiol. 10.1002/dneu.22391.
14. Rheaume, B.A., Jereen, A., Bolisetty, M., Sajid, M.S., Yang, Y., Renna, K., Sun, L., Robson, P., and Trakhtenberg, E.F. (2018). Single cell transcriptome profiling of retinal ganglion cells identifies cellular subtypes. Nat Commun *9*, 2759. 10.1038/s41467-018-05134-3.

15. Xing, J., Lukomska, A., Rheaume, B.A., Kim, J., Sajid, M.S., Damania, A., and Trakhtenberg, E.F. (2023). Post-injury born oligodendrocytes incorporate into the glial scar and contribute to the inhibition of axon regeneration. Development. 10.1242/dev.201311.

16. Rheaume, B.A., Xing, J., Lukomska, A., Theune, W.C., Damania, A., Sjogren, G., and Trakhtenberg, E.F. (2023). Pten inhibition dedifferentiates long-distance axon-regenerating intrinsically photosensitive retinal ganglion cells and upregulates mitochondria-associated Dynlt1a and Lars2. Development *150*. 10.1242/dev.201644.

17. Matsuda, T., Irie, T., Katsurabayashi, S., Hayashi, Y., Nagai, T., Hamazaki, N., Adefuin, A.M.D., Miura, F., Ito, T., Kimura, H., Shirahige, K., et al. (2019). Pioneer Factor NeuroD1 Rearranges Transcriptional and Epigenetic Profiles to Execute Microglia-Neuron Conversion. Neuron *101*, 472-485.e477. 10.1016/j.neuron.2018.12.010.

18. Li, Y., Schlamp, C.L., and Nickells, R.W. (1999). Experimental induction of retinal ganglion cell death in adult mice. Invest Ophthalmol Vis Sci *40*, 1004-1008.

19. Ho, K.H., and Patrizi, A. (2021). Assessment of common housekeeping genes as reference for gene expression studies using RT-qPCR in mouse choroid plexus. Sci Rep *11*, 3278. 10.1038/s41598-021-82800-5.

20. Trakhtenberg, E.F., Pho, N., Holton, K.M., Chittenden, T.W., Goldberg, J.L., and Dong, L. (2016). Cell types differ in global coordination of splicing and proportion of highly expressed genes. Sci Rep *6*, 32249. 10.1038/srep32249.

21. Patro, R., Duggal, G., Love, M.I., Irizarry, R.A., and Kingsford, C. (2017). Salmon provides fast and bias-aware quantification of transcript expression. Nat Methods *14*, 417-419. 10.1038/nmeth.4197.

22. Love, M.I., Huber, W., and Anders, S. (2014). Moderated estimation of fold change and dispersion for RNA-seq data with DESeq2. Genome Biol *15*, 550. 10.1186/s13059-014-0550-8.

23. Robinson, J.T., Thorvaldsdóttir, H., Winckler, W., Guttman, M., Lander, E.S., Getz, G., and Mesirov, J.P. (2011). Integrative genomics viewer. Nat Biotechnol *29*, 24-26. 10.1038/nbt.1754.

24. Stuart, T., Butler, A., Hoffman, P., Hafemeister, C., Papalexi, E., Mauck, W.M., Hao, Y., Stoeckius, M., Smibert, P., and Satija, R. (2019). Comprehensive Integration of Single-Cell Data. Cell *177*, 1888-1902.e1821. 10.1016/j.cell.2019.05.031.

25. Hao, Y., Hao, S., Andersen-Nissen, E., Mauck, W.M., Zheng, S., Butler, A., Lee, M.J., Wilk, A.J., Darby, C., Zager, M., Hoffman, P., et al. (2021). Integrated analysis of multimodal single-cell data. Cell *184*, 3573-3587.e3529. 10.1016/j.cell.2021.04.048.

26. Tran, N.M., Shekhar, K., Whitney, I.E., Jacobi, A., Benhar, I., Hong, G., Yan, W., Adiconis, X., Arnold, M.E., Lee, J.M., Levin, J.Z., et al. (2019). Single-Cell Profiles of Retinal Ganglion Cells Differing in Resilience to Injury Reveal Neuroprotective Genes. Neuron *104*, 1039-1055.e1012. 10.1016/j.neuron.2019.11.006.

27. Wickham, H. (2009). ggplot2: Elegant Graphics for Data Analysis (New York, NY: Springer).

28. Enright, A.J., John, B., Gaul, U., Tuschl, T., Sander, C., and Marks, D.S. (2003). MicroRNA targets in Drosophila. Genome Biol *5*, R1. 10.1186/gb-2003-5-1-r1.

29. John, B., Enright, A.J., Aravin, A., Tuschl, T., Sander, C., and Marks, D.S. (2004). Human MicroRNA targets. PLoS Biol *2*, e363. 10.1371/journal.pbio.0020363.

30. Lo Giudice, Q., Leleu, M., La Manno, G., and Fabre, P.J. (2019). Single-cell transcriptional logic of cell-fate specification and axon guidance in early-born retinal neurons. Development *146*. 10.1242/dev.178103.

31. Jacobi, A., Tran, N.M., Yan, W., Benhar, I., Tian, F., Schaffer, R., He, Z., and Sanes, J.R. (2022). Overlapping transcriptional programs promote survival and axonal regeneration of injured retinal ganglion cells. Neuron *110*, 2625-2645.e2627. 10.1016/j.neuron.2022.06.002.

32. Kearney, A., Lukomska, A., Brady, J., Damania, A., Gupta, M., and Trakhtenberg, E.F. (2026). Vacuolar ATPase subunit Atp6v0c transgene promotes neuroprotection and long-distance axon regeneration in injured retinal ganglion neurons. Mol Ther Nucleic Acids *37*, 102922. 10.1016/j.omtn.2026.102922.

33. Ashburner, M., Ball, C.A., Blake, J.A., Botstein, D., Butler, H., Cherry, J.M., Davis, A.P., Dolinski, K., Dwight, S.S., Eppig, J.T., Harris, M.A., et al. (2000). Gene ontology: tool for the unification of biology. The Gene Ontology Consortium. Nat Genet *25*, 25-29. 10.1038/75556.

34. Aleksander, S.A., Balhoff, J., Carbon, S., Cherry, J.M., Drabkin, H.J., Ebert, D., Feuermann, M., Gaudet, P., Harris, N.L., Hill, D.P., Lee, R., et al. (2023). The Gene Ontology knowledgebase in 2023. Genetics *224*. 10.1093/genetics/iyad031.

35. Yu, G. (2025). enrichplot: Visualization of Functional Enrichment Result. Bioconductor *v.1.28.4*. 10.18129/B9.bioc.enrichplot.

36. Kolberg, L., Raudvere, U., Kuzmin, I., Vilo, J., and Peterson, H. (2020). gprofiler2 -- an R package for gene list functional enrichment analysis and namespace conversion toolset g:Profiler. F1000Res *9*. 10.12688/f1000research.24956.2.

37. Wu, T., Hu, E., Xu, S., Chen, M., Guo, P., Dai, Z., Feng, T., Zhou, L., Tang, W., Zhan, L., Fu, X., et al. (2021). clusterProfiler 4.0: A universal enrichment tool for interpreting omics data. Innovation (Camb) *2*, 100141. 10.1016/j.xinn.2021.100141.

38. D'souza, C., Lukomska, A., Balaji, A.J., Brady, J., and Trakhtenberg, E.F. (2026). Targeting neurodevelopmental miR132-3p promotes neuroprotection and axon regeneration after optic nerve injury in mice. Brain Res *1889*, 150450. 10.1016/j.brainres.2026.150450.
